# A sperm–oocyte protein partnership required for egg activation in *Caenorhabditis elegans*

**DOI:** 10.1242/dev.204674

**Published:** 2025-06-25

**Authors:** Tatsuya Tsukamoto, Ji Kent Kwah, Mark E. Zweifel, Naomi Courtemanche, Micah D. Gearhart, Katherine M. Walstrom, Aimee Jaramillo-Lambert, David Greenstein

**Affiliations:** ^1^ Department of Genetics, Cell Biology and Development, University of Minnesota, Minneapolis, MN 55455, USA; ^2^ Department of Biological Sciences, University of Delaware, Newark, DE 19716, USA; ^3^ Department of Obstetrics, Gynecology, and Women's Health, University of Minnesota Medical School, Minneapolis, MN 55455, USA

**Keywords:** Fertilization, Egg activation, Oocyte meiosis, Meiotic cytokinesis, Actin regulation

## Abstract

Fertilization triggers the completion of female meiosis and launches the oocyte-to-embryo transition. *Caenorhabditis elegans spe-11* is one of the few known paternal-effect embryonic lethal genes. We report that the sperm protein SPE-11 forms a complex with an oocyte protein, OOPS-1 (Oocyte Partner of SPE-11) at fertilization, and that the protein complex is required for the completion of meiosis, the block to polyspermy, and eggshell formation. Consistent with the molecular interaction of their encoded proteins, *oops-1* and *spe-11* exhibit indistinguishable null phenotypes in which fertilized oocytes arrest in meiosis I or meiosis II or fail to complete the actin-based process of meiotic cytokinesis. Biochemical analysis shows that the complex binds F-actin in the absence of other proteins and inhibits the nucleation of actin filaments at substoichiometric concentrations. Both OOPS-1 and SPE-11 are intrinsically disordered proteins that are highly phosphorylated, and biochemical and genetic experiments define interactions with the sperm-specific protein phosphatase 1 homologs GSP-3/4. Genetic results suggest that the cortical EGG complex recruits the OOPS-1–SPE-11 complex at fertilization, which promotes meiotic cytokinesis and in turn activates synthesis of the eggshell.

## Teaser

**Summary:** This study reports the discovery of a sperm–oocyte protein partnership that is required for early post-fertilization development in the nematode *Caenorhabditis elegans*.

## INTRODUCTION

In most sexually reproducing animals, oocytes arrest in meiotic prophase for a prolonged period ­– up to 50 years in humans. Meiosis resumes in response to hormonal signaling in the process of meiotic maturation. During meiotic maturation, the nuclear envelope of the oocyte breaks down (NEBD) in response to the activation of CDK1/cyclin B, the maturation-promoting factor. At NEBD, microtubules gain access to the bivalents and the acentriolar meiotic spindle assembles. Fertilization triggers the process of egg activation, which results in the completion of oocyte meiosis, although the molecular mechanisms by which sperm activate embryonic development are not fully understood. Defects in oocyte meiosis and early post-fertilization development are a major cause of infertility, miscarriage, and human birth defects, and basic studies of early post-fertilization development in multiple model systems have proved informative (Gruhn and Hoffman, 2022).

The timing of the meiotic divisions with respect to fertilization varies with the species. In humans, the first meiotic division is completed before fertilization, with the second division occurring after fertilization. By contrast, in the nematode *Caenorhabditis elegans*, both meiotic divisions happen after fertilization (Albertson and Thomson, 1993; McNally and McNally, 2005). In *C. elegans*, sperm promote the completion of oocyte meiosis at several levels. The major sperm protein (MSP), which functions as the chief cytoskeletal element underlying amoeboid locomotion of nematode sperm (Italiano et al., 1996), functions as a hormone that triggers oocyte meiotic maturation and ovulation (Miller et al., 2001; Kosinski et al., 2005). During the first meiotic division, half of the homologous chromosomes are extruded in the first polar body, and half of the remaining sister chromatids are deposited in the second polar body during meiosis II. A key aspect of oocyte meiosis is that the asymmetric cell divisions that form small polar bodies and a large embryo depend on the assembly of the meiotic contractile actin ring at the cell cortex immediately adjacent to one pole of the meiotic spindle. The sperm-supplied GSP-3/4 protein phosphatase 1 homologs and the GSKL-1/2 glycogen synthase kinase homologs function with the oocyte MEMI-1–3 proteins to promote meiosis II after fertilization (Ataeian et al., 2016; Banerjee and Srayko, 2022). However, the role of sperm-supplied factors in promoting meiosis I has been less clear.

In *C. elegans*, strict paternal-effect embryonic lethal mutations in *spe-11* interfere with meiotic cytokinesis, polar body formation, synthesis of the eggshell, and the block to polyspermy (L'Hernault et al., 1988; Hill et al., 1989; McNally and McNally, 2005; Johnston et al., 2010; Sato and Sato, 2011). SPE-11 encodes a hydrophilic protein that appears to lack homologs outside of Caenorhabditid nematodes (Browning and Strome, 1996). In sperm, SPE-11 localizes to the perinuclear RNA halo (Browning and Strome, 1996), which is abnormal in *spe-11* mutants (Ward et al., 1981). RNA induces SPE-11 to undergo phase separation *in vitro*, and it has been proposed that SPE-11 associates with sperm RNAs that are delivered at fertilization (Li et al., 2023). Yet the finding that SPE-11 can promote embryonic development when provided to the embryo through the maternal germline (Browning and Strome, 1996) suggests that its perinuclear localization in sperm may not be required. Despite the status of SPE-11 as one of the few known paternal-effect mutations, its molecular interactions and activities were unknown.

Here, we identify Oocyte Partner of SPE-11 (OOPS-1), which forms a complex with SPE-11 at fertilization. We show that the complex is required for multiple aspects of the egg activation process, including the completion of meiosis, eggshell formation, and the block to polyspermy. We present biochemical evidence that the OOPS-1–SPE-11 complex can function as an actin regulator. The biochemical and genetic results presented here identify molecular associations and activities of the OOPS-1–SPE-11 complex that begin to explain its essential roles.

## RESULTS

### Identification of the oocyte partner of *spe-11*

**Fig. 1.**
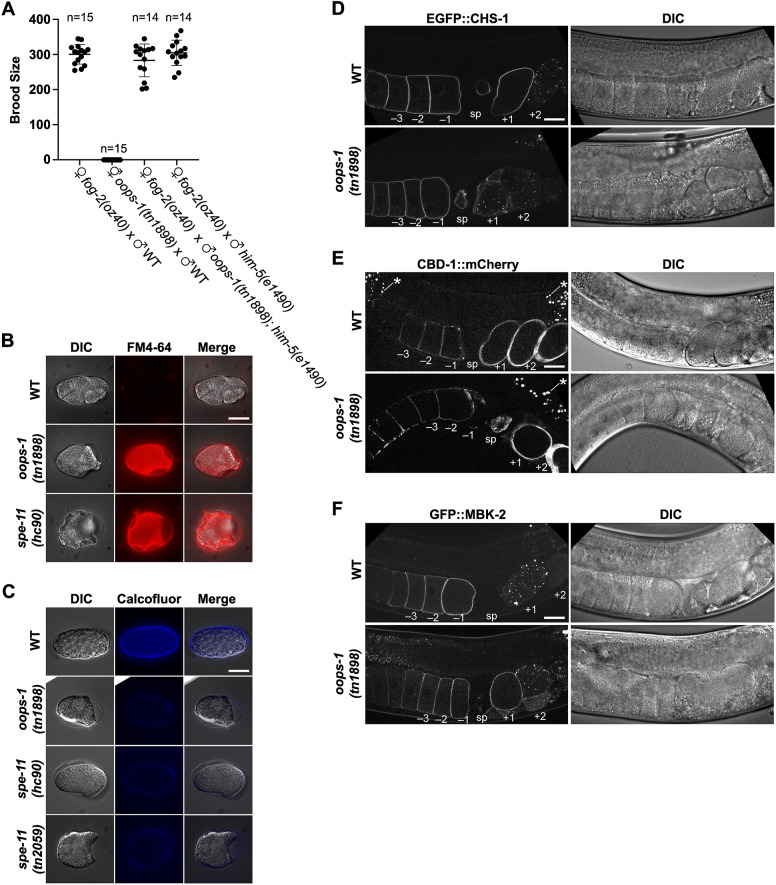
***oops-1* and *spe-11* mutant embryos exhibit a defect in chitin deposition.** (A) Brood size of mating assays between *oops-1(tn1898)* null mutants and the wild type. (B) FM4-64 staining showing the permeability layer is disrupted in mutant embryos. (C) Calcofluor staining to detect the chitin layer of the eggshell. (D-F) Expression of EGFP::CHS-1, CBD-1::mCherry and GFP::MBK-2 in the wild type and *oops-1(tn1898)* mutants. All three egg activation proteins exhibit cortical staining in oocytes and newly fertilized embryos and are subsequently internalized and degraded. Proximal oocytes (–1 to −3), the spermatheca (sp) and newly fertilized embryos (+1 and +2) are indicated. Asterisks indicate gut granules in the intestine (E). DIC, differential interference contrast; WT, wild type. Images are representative of at least 20 replicates. Scale bars: 20 µm.

The RNA-binding proteins OMA-1 and LIN-41 control the translation of genes that play important roles during oocyte development or the oocyte-to-embryo transition (Spike et al., 2014a,b; Tsukamoto et al., 2017). The set of mRNAs that associate with OMA-1 and/or LIN-41 (*n*=2668; fourfold enrichment, *P*<0.05) also contains many uncharacterized genes (∼50%) (Spike et al., 2014b; Tsukamoto et al., 2017). We screened gene knockout databases for OMA-1 and LIN-41 target genes with deletion alleles annotated as being sterile or lethal (*n*=90; *C. elegans* Deletion Mutant Consortium, 2012). We found one gene, *C31H1.8*, defined by the *tm6141* deletion allele, that is required in the female germline for early post-fertilization development – the completion of meiosis, eggshell formation, and the block to polyspermy – hereafter referred to as *oops-1* (oocyte partner of *spe-11*), as described below. To confirm our initial observations with *oops-1(tm6141)*, we used clustered regularly interspaced short palindromic repeats (CRISPR)-Cas9 genome editing to delete the entire open reading frame to generate the *oops-1(tn1898)* null allele (Fig. S1A). *oops-1(tn1898)* mutant hermaphrodites or females produced all inviable embryos even when mated to wild-type males (Fig. 1A). Since *oops-1* mutant males efficiently sire progeny (Fig. 1A), we conclude that the defect is on the oocyte side.

Oocytes in *oops-1* mutant hermaphrodites stacked up in the gonad arm on the second day of adulthood (Fig. S2), a phenotype associated with depletion of the sperm-provided meiotic maturation signal (McCarter et al., 1999; Miller et al., 2001). Although there are several ways this phenotype can arise, hermaphrodites with mutations that affect fertilization or sperm activation exhibit nearly normal rates of meiotic maturation on the second day of adulthood and do not display the oocyte-stacking phenotype (Kosinski et al., 2005). Thus, we investigated whether sperm might be consumed at a higher rate in *oops-1(tn1898)* mutants owing to polyspermy (in which more than one sperm fertilizes an oocyte).

To assess polyspermy, *fog-2(oz40); his-72(uge30[gfp::his-72])* females, with or without the *oops-1(tn1898)* null mutation, were first treated with *mat-1(RNAi)* prior to mating. *mat-1* encodes a subunit of the anaphase-promoting complex. This treatment blocks the embryos at metaphase of meiosis I so that the sperm chromatin can be scored after mating. GFP::HIS-72 marks the oocyte-contributed chromatin (Delaney et al., 2018), enabling scoring of the unmarked paternal chromatin, which remains highly condensed, typically in the periphery distinct from the maternally contributed chromatin. We observed that one-third of the embryos of *oops-1(tn1898)* mated females exhibited polyspermy, which was not observed in wild-type controls (Table 1). Consistent with the observation that the synthesis of the chitinous layer of the eggshell is required to prevent polyspermy (Johnston et al., 2010), *oops-1(tn1898)* mutant embryos were osmotically sensitive, failed to establish a permeability barrier, and exhibited a defect in chitin deposition (Fig. 1B,C).

**Table 1.** *oops-1* mutants exhibit polyspermy

Relevant oocyte genotype^‡^	Number of sperm observed in the embryo*			
	0	1	2	3
Wild type (*n*=203)	3% (*n*=6)	97% (*n*=197)	0% (*n*=0)	0% (*n*=0)
*oops-1(tn1898)* (*n*=141)	13.5% (*n*=19)	53.2% (*n*=75)	31.2% (*n*=44)	2.1% (*n*=3)

*Mating was to *plg-1(e2001); him-5(e1490)* males. The *plg-1* mutation was used to confirm mating via the deposition of a mating plug.

^‡^The genotype of the females also included *fog-2(oz40); his-72(uge30[gfp::his-72])*. The females were first treated with *mat-1(RNAi)* to arrest the cell cycle post-fertilization; otherwise, the maternal and paternal chromatin will replicate, which would confound the scoring of polyspermy.

The chitin layer of the eggshell is synthesized by chitin synthase (CHS-1), which is a component of the EGG complex that contains proteins needed for fertilization and egg activation, including EGG-1–5 and the MBK-2 kinase (Maruyama et al., 2007; Parry et al., 2009). Similarly, the PERM complex is required for the proper formation of the outer layer of the eggshell (González et al., 2018). The chitin-binding protein CBD-1 serves to anchor and organize the EGG and PERM complexes (Johnston et al., 2010; González et al., 2018). Therefore, we tested the possibility that the *oops-1(tn1898)* mutation interferes with eggshell formation by causing mislocalization of EGG or PERM complex proteins in oocytes or early embryos. We observed that CHS-1, and the EGG complex components EGG-1–3, and MBK-2, as well as the PERM complex components CBD-1, PERM-2 and PERM-4 (González et al., 2018), localize normally in *oops-1(tn1898)* null mutants (Fig. 1D-F, Fig. S3). These results, taken together with genetic results described below, suggest that OOPS-1 promotes EGG complex activity independently of localization.

### *oops-1* is required for the completion of oocyte meiosis

**Fig. 2.**
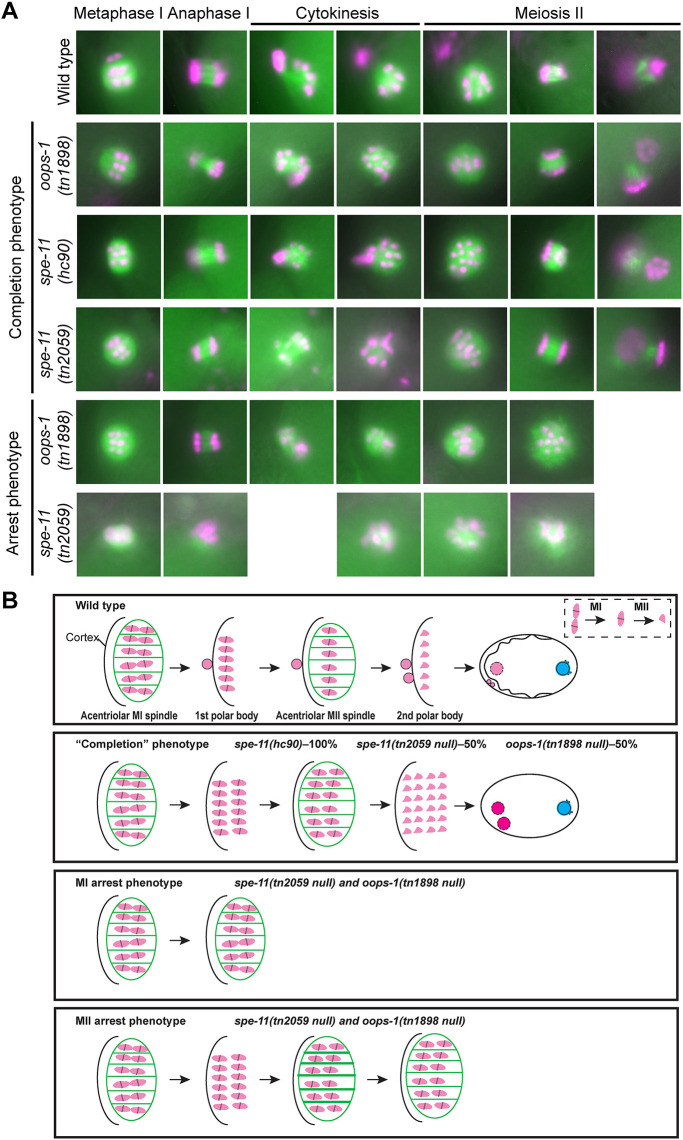
***oops-1* and *spe-11* null mutant embryos exhibit defects in the completion of oocyte meiosis.** (A) Analysis of *oops-1* and *spe-11* mutants by live imaging. In the wild type, the MI and MII spindles form sequentially, chromosomes segregate and two polar bodies form. In the *spe-11(hc90)* reduction-of-function mutant, the MI and MII spindles form and chromosomes segregate but polar bodies do not form; this is referred to as the ‘completion phenotype’. The completion phenotype is observed in approximately 50% of *oops-1(tn1898)* and *spe-11(tn2059)* null mutants. The null mutants also exhibit arrest in MI [as in the *spe-11(tn2059)* example shown] or MII [as in the *oops-1(tn1898)* example shown]. Chromatin is labeled with mCherry (red) and microtubules are labeled with GFP (green). Images are representative of 19-41 replicates. (B) Summary of oocyte meiosis in the wild type and mutants. The inset in the top panel shows that homologs segregate in MI and sister chromatids segregate in MII. In the ‘completion phenotype’, the internalized sets of chromosomes often form multiple nuclei. Meiotic cytokinesis (polar body formation) does not occur.

Because *oops-1* mutant embryos arrest at the one-cell stage without the formation of polar bodies, we examined oocyte meiosis and early embryogenesis at high resolution using *in utero* time-lapse recordings. In these experiments, the meiotic spindles and chromatin were visualized using β-tubulin::GFP and mCherry::histone, respectively (Table 2, Movies 1-3). To ensure phototoxicity is not an issue, we analyzed wild-type embryos, all of which completed meiosis I and II and formed polar bodies (Table 2, Fig. 2). We observed that approximately half of the *oops-1(tn1898)* mutant embryos exhibited a phenotype in which oocyte meiotic chromosomes segregate at anaphase I and II but fail to form polar bodies (Table 2, Fig. 2, Movie 2; referred to as the ‘completion phenotype’). By contrast, the other half displayed meiotic arrest (Movie 3). The meiotic arrest phenotypes were striking. In these embryos, we observed that the meiotic spindle fails to properly shorten and maintain its association with the cortex. Instead, the spindle drifts away from the cortex and fails to maintain its structure, suggesting that the cytoskeletal forces that maintain its cortical association and integrity are deficient (Movie 3). Using time-lapse microscopy, we also recorded an instance of polyspermy (Movie 4).

**Table 2.** Quantification of meiotic defects in *oops-1* and *spe-11* mutants

Genotype	Pre-anaphase I arrest	Anaphase I arrest	Meiosis II arrest	Meiotic exit*	Polar body extrusion
Wild type	0% (0/20)	0% (0/20)	0% (0/20)	100% (20/20)	100% (20/20)
*oops-1(tn1898)*	31.7% (13/41)^‡^	21.9% (9/41)^‡^	7.4% (3/41)^‡^	39.0% (16/41)^‡^	0% (0/41)
*spe-11(hc90)*	0% (0/22)	0% (0/22)	0% (0/22)	100% (22/22)	0% (0/22)
*spe-11(tn2059)*	31.6% (6/19)^‡^	10.5% (2/19)^‡^	10.5% (2/19)^‡^	47.4% (9/19)^‡^	0% (0/19)
*spe-11(tn2059); oops-1(tn1898)*	21.1% (4/19)^‡^	36.8% (7/19)^‡^	0% (0/19)^‡^	42.1% (8/19)^‡^	0% (0/19)

*The mutant embryos that exit meiosis without producing polar bodies correspond to those exhibiting the ‘completion’ phenotype (Fig. 2).

^‡^*P*>0.1 when all pairwise comparisons were made between the corresponding values for *oops-1(tn1898)*, *spe-11(tn2059*), and *spe-11(tn2059); oops-1(tn1898)* double mutants using Fisher's exact test. See Table S3 for the contingency tables and the exact *P*-values.

### OOPS-1 interacts with SPE-11

OOPS-1 is predicted to be an intrinsically disordered protein (IDP) using IUPRED2 (Mészáros et al., 2018) and AlphaFold (Jumper et al., 2021) with apparent homologs restricted to Caenorhabditid nematodes. To understand how OOPS-1 functions, we conducted OOPS-1 tandem-affinity purification (TAP) and mass spectrometry (Fig. S4A,C). Three biological replicates identified SPE-11 (Table 3, Table S1). *spe-11* is one of the few known strict paternal-effect lethal mutations (L'Hernault et al., 1988). We also conducted SPE-11 TAP (Fig. S4B,D), which recovered OOPS-1 with high efficiency (Table 3, Table S2). In six of seven of these TAPs, we isolated the nearly identical sperm-specific GSP-3/4 serine/threonine protein phosphatases, which are homologous to human protein phosphatase 1 catalytic subunits (Chu et al., 2006; Wu et al., 2012).

**Table 3.** Tandem affinity purification of OOPS-1 and SPE-11

	OOPS-1 TAP* protein coverage^§^ (%)			SPE-11 TAP^‡^ protein coverage^§^ (%)			
Protein^¶^	Exp. I	Exp. II	Exp. III	Exp. IV	Exp. V	Exp. VI	Exp. VII
OOPS-1	30.8	45.5	33.1	65.5	59.9	62.7	72.7
SPE-11	32.4	54.8	37.1	78.6	68.9	85.3	87.6
GSP-3**	14.8	11.8	0.0	48.5	38.7	37.4	63.6
GSP-4**	14.8	11.8	0.0	48.5	38.7	37.4	63.6

*Experiments I-III were conducted using DG4800 *oops-1(tn1908[gfp::tev::3xflag::oops-1])* adult hermaphrodites (Fig. S1).

^‡^Experiments IV-VII were conducted using DG5430 *spe-11(tn2094[gfp::tev::3xflag::spe-11])* animals (Fig. S6), which were adult hermaphrodites (Experiments IV and V) or a mixture of adult males and adult hermaphrodites using the DG5462 *spe-11(tn2094[gfp::tev::3xflag::spe-11])*; *him-5(e1490)* strain (Experiments VI and VII).

^§^Instrumentation was upgraded between experiments III and IV and thus the OOPS-1 and SPE-11 TAPs are not directly comparable in terms of the absolute values of the percentage protein coverage.

^¶^Proteins shared between OOPS-1 and SPE-11 TAPs. A full list of all proteins detected can be found in Tables S1 and S2.

**Many of the peptides assigned to GSP-3 and GSP-4 are indistinguishable.

**Fig. 3.**
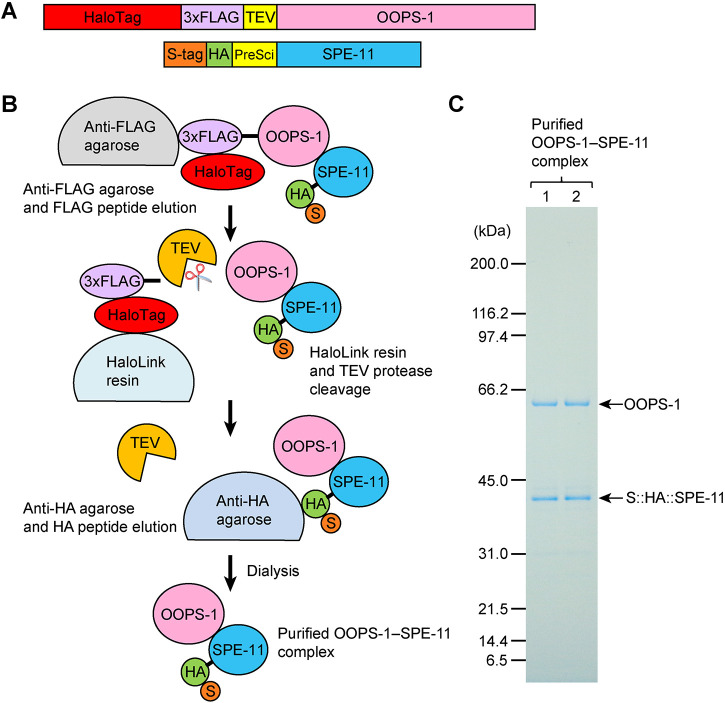
**Purification of the OOPS-1–SPE-11 complex.** (A) Affinity-tagged versions of OOPS-1 and SPE-11 were co-expressed in *E. coli.* (B) The scheme used to purify the complex using affinity chromatography. (C) A colloidal Coomassie-stained protein gel with two lanes of the purified complex (see Fig. S5C for gel analysis of intermediate steps of the purification).

To determine whether OOPS-1 and SPE-11 can interact in the absence of other *C. elegans* proteins, we co-expressed tagged proteins in *Escherichia coli*. Affinity purification established that OOPS-1 and SPE-11 form a stable complex (Fig. 3, Fig. S5).

### Null mutations in *spe-11* phenocopy null mutations in *oops-1*

The meiotic phenotypes observed in *oops-1(tn1898)* null mutants are slightly different from what was reported previously for the *spe-11(hc90)* mutation (McNally and McNally, 2005), which contains a premature termination codon at position W191 (Browning and Strome, 1996; SPE-11 has 299 aa). Specifically, all *spe-11(hc90)* mutant embryos observed by McNally and McNally (2005; *n*=5) exhibited a phenotype in which oocyte meiotic chromosomes segregate at anaphase I and II but fail to form polar bodies, similar to the completion phenotype described above that we observed in half of the *oops-1(tn1898)* null mutants. We replicated this finding, exclusively observing the completion phenotype in *spe-11(hc90)* mutants (*n*=22; Table 2, Fig. 2, Movie 5). However, prior genetic results could not exclude the possibility that *spe-11(hc90)* reduces but does not eliminate *spe-11* function (L'Hernault et al., 1988). Thus, we used CRISPR-Cas9 genome editing to delete the entire *spe-11* open reading frame to generate the *spe-11(tn2059)* null mutation (Fig. S6A). Imaging revealed that *spe-11(tn2059)* null mutants phenocopy the *oops-1(tn1898)* mutant phenotype in displaying either the completion phenotype or meiotic arrest (Table 2, Fig. 2, Movies 6, 7). A comparison of the distribution of phenotypes observed in *oops-1(tn1898)* and *spe-11(tn2059)* null mutants shows that they are not statistically different (Fisher's exact test; Table 2, Table S3). Similarly, statistical analysis of the distribution of phenotypes in *spe-11(tn2059); oops-1(tn1898)* double-null mutants indicates they are no different from those displayed by the individual single mutants (Table 2, Table S3). However, instances of meiosis I arrest, but not meiosis II arrest, were observed in *spe-11(tn2059); oops-1(tn1898)* double mutants (Table 2). Thus, we cannot exclude the possibility that OOPS-1 and SPE-11 can each exhibit residual activity in the absence of one another.

### OOPS-1 and SPE-11 exhibit complementary expression patterns

**Fig. 4.**
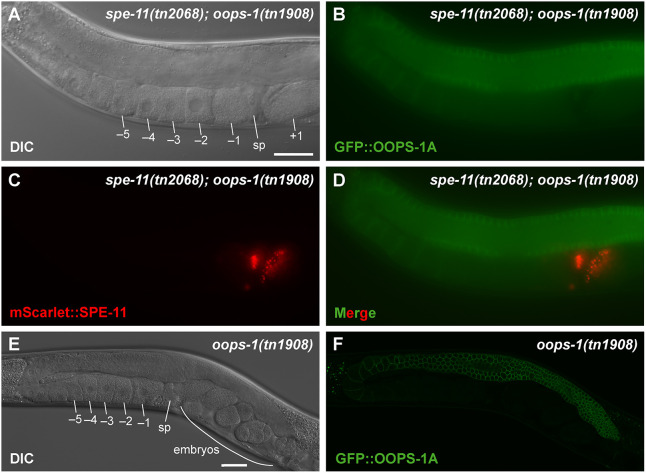
**OOPS-1 and SPE-11 exhibit complementary expression patterns.** (A-D) Differential interference contrast (DIC) (A) and wide-field fluorescence (B-D) micrographs of a *spe-11(tn2068[mScarlet::tev::3xflag::spe-11]); oops-1(tn1908[gfp::tev::3xflag::oops-1a])* adult hermaphrodite expressing GFP::OOPS-1A (green) and mScarlet::SPE-11 (red). OOPS-1A is expressed throughout the female germline and becomes downregulated in oocytes. SPE-11 expression is restricted to sperm. The proteins encounter each other upon fertilization but are at low levels. (E,F) DIC (E) and a single optical section of a confocal fluorescence micrograph (F) of an *oops-1(tn1908[gfp::tev::3xflag::oops-1a]* adult hermaphrodite high-lighting the cortical localization of GFP::OOPS-1A in the distal germline and its downregulation in proximal oocytes. Proximal oocytes (–1 to −5), the spermatheca (sp) and a newly fertilized embryo (+1) and older embryos in the uterus are indicated. Images are representative of at least 100 replicates. Scale bars: 30 µm.

Because OOPS-1 and SPE-11 are required by gametes of opposite sex, we compared their expression patterns. We generated viable and fertile N- and C-terminal fusions of OOPS-1 to GFP using CRISPR-Cas9 genome editing (Fig. S1B). GFP::OOPS-1 expression was observed throughout the adult hermaphrodite germline and was enriched at the cell cortex of distal germ cells and developing oocytes; however, cortical localization became less apparent, and expression levels declined in the most fully grown oocytes (Fig. 4A-D). The decline in OOPS-1 levels during late oogenesis was most apparent from the analysis of single confocal sections (Fig. 4E,F).

WormBase (Sternberg et al., 2024) predicts four potential OOPS-1 isoforms (A-D), which fall into two classes: isoforms OOPS-1A/C include exons 1-3; and OOPS-1B/D start within exon 3 (Fig. S1B; isoforms C and D are 2-aa-shorter versions of isoforms A and B, respectively). Because the N-terminally tagged allele yielded an expression pattern similar to that produced by tagging at the C terminus (Fig. 4A-D, Fig. S7A-D), which would label all isoforms, OOPS-1A/C are likely the functional forms. Consistent with this, the *oops-1(gk503838)* allele, which alters the initiator methionine of OOPS-1B/D to an isoleucine, was viable and fertile (Fig. S1A). To examine whether OOPS-1B/D might be functional if made, we generated a 407-bp deletion (corresponding to 103 aa) in the N-terminally tagged *oops-1(tn1908)* strain to produce a new allele, *oops-1(tn1908 tn2000)*, which cannot produce isoforms OOPS-1A/C, and which tags OOPS-1B/D (Fig. S1B). GFP::OOPS-1B/D localized to the cytoplasm and was abundant in oocytes but lacked the cortical enrichment observed in the germline in OOPS-1A/C (Fig. S7E-H). Thus, an N-terminal domain within OOPS-1A/C is responsible for cortical tethering in the germline. This cortical tethering domain was dispensable for post-fertilization development, with the caveat that GFP::OOPS-1B/D is expressed at high levels (Fig. S7E-H). Thus, the N-terminal domain limits OOPS-1 levels.

Likewise, we generated viable and fertile alleles of SPE-11 tagged at the N terminus with mScarlet or GFP (Fig. S6B). In the adult hermaphrodite gonad, the only cells observed to express mScarlet::SPE-11 were sperm (Fig. 4A-D). Because OOPS-1 and SPE-11 are expressed and required in oocytes and sperm, respectively, the OOPS-1–SPE-11 complex likely forms and functions upon fertilization. We were unable to visualize the predicted formation of the complex after fertilization, consistent with the results of Browning and Strome (1996), who used immunofluorescence to examine SPE-11 localization after fertilization and concluded it was below the level of detection. Immunofluorescence is more sensitive than fluorescent protein detection because of signal amplification and quantum yield effects. Nevertheless, our genetic analysis demonstrates that OOPS-1 and SPE-11 are required and expressed in female and male gametes, respectively. The biochemical analysis shows the two proteins form a complex that can be isolated from *C. elegans* and after co-expression in *E. coli* (Table 3, Fig. 3). Given that the *oops-1* and *spe-11* null mutant phenotypes are indistinguishable in their impact on early post-fertilization development, these data suggest that OOPS-1 and SPE-11 interact and function after fertilization.

### SPE-11 can function when expressed in the oocyte, but OOPS-1 cannot function efficiently when expressed in sperm

**Fig. 5.**
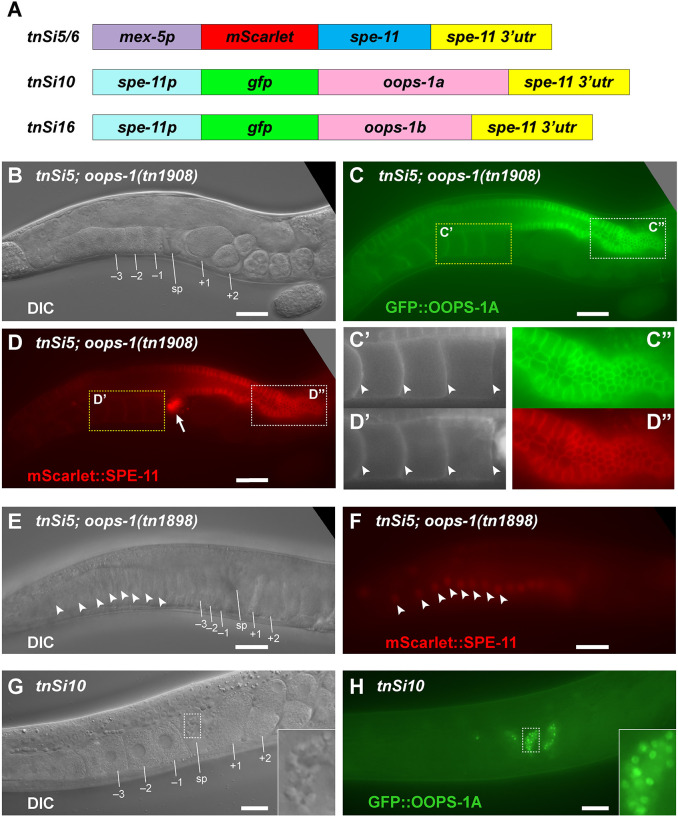
**Ectopic expression of SPE-11 in the female germline and OOPS-1 in the male germline.** (A) Single-copy insertion constructs to express SPE-11 in the female germline and OOPS-1A and OOPS-1B in the male germline. (B-D″) DIC (B) and fluorescence micrographs of a *tnSi5; oops-1(tn1908[gfp::tev::3xflag::oops-1a])* adult hermaphrodite expressing GFP::OOPS-1A (C) and mScarlet::SPE-11 (D). In addition to the female germline, *tnSi*5 expresses mScarlet::SPE-11 in sperm (arrow). Insets focus on proximal oocytes (C′,D′) and the distal region of the germline (C″,D″). Note: mScarlet::SPE-11 and GFP::OOPS-1A are prominent at the cortex of germ cells and oocytes (arrowheads in C′,D′). (E,F) DIC (E) and fluorescence (F) micrographs of a *tnSi5; oops-1(tn1898* null*)* adult hermaphrodite. Note: mScarlet::SPE-11 exhibits nuclear localization in the absence of OOPS-1 (several oocyte nuclei are indicated by arrowheads in E,F). Thus, the cortical localization of mScarlet::SPE-11 observed when SPE-11 is ectopically expressed in the female germline is dependent on OOPS-1. (G,H) DIC (G) and fluorescence (H) micrographs of a *tnSi10* adult hermaphrodite expressing GFP::OOPS-1A in sperm. Insets highlight localization to sperm. Proximal oocytes (–1 to −3), the spermatheca (sp) and newly fertilized embryos (+1 and +2) are indicated. Images are representative of at least 100 replicates. Scale bars: 30 µm (B-F); 20 µm (G,H).

Browning and Strome (1996) used a heat shock promoter to express SPE-11 during oogenesis and observed transient rescue of fertility in *spe-11(hc90)* mutants. Using the tools available at the time, it was not possible to evaluate quantitatively the efficiency of the rescue. We generated two independent single-copy insertions (*tnSi5* and *tnSi6*) in which mScarlet::SPE-11 is expressed under control of the *mex-5* promoter and the *spe-11* 3′UTR (Fig. 5A). When we expressed mScarlet::SPE-11 in the female germline, we observed localization to the cortex of germ cells and oocytes in a pattern similar to GFP::OOPS-1 (Fig. 5B-D″). We did not observe this cortical localization in *oops-1(tn1898)* null mutants, and instead mScarlet::SPE-11 exhibited nuclear localization (Fig. 5E,F). This observation is consistent with our biochemical analyses and suggests that OOPS-1 and SPE-11 can interact *in vivo.*

We observed that mScarlet::SPE-11 expression using the *mex-5* promoter and the *spe-11* 3′UTR (*tnSi5* and *tnSi6*) was able to rescue *spe-11(tn2059)* null mutants to fertility; however, these constructs were expressed in both oocytes and sperm (Fig. 5D). To test whether mScarlet::SPE-11 expression exclusively in the female germline rescues the *spe-11* mutant phenotype, we used the *fog-2(oz40)* mutation to feminize the germline and mated the *tnSi5; fog-2(oz40)* and *tnSi6; fog-2(oz40)* females with *spe-11(tn2059)* null mutant males. We observed 96-98% embryonic viability, indicating that SPE-11 can efficiently mediate post-fertilization development when delivered through the oocyte (Table 4). By contrast, viable cross progeny were never observed when *fog-2(oz40)* females were crossed with *spe-11(tn2059)* null mutant males (Table 4).

**Table 4.** SPE-11 can function via the oocyte, but provision of OOPS-1 by sperm is unable to fully support development

Female	Male	Viability (%)
*fog-2(oz40)*	*spe-11(tn2059); him-5(e1490)*	0 (*n*=9473)
tnSi5*; fog-2(oz40)	*spe-11(tn2059); him-5(e1490)*	96.3 (*n*=28,750)
*tnSi6* ^‡^ *; fog-2(oz40)*	*spe-11(tn2059); him-5(e1490)*	98.0 (*n*=31,126)
tnSi5*; fog-2(oz40)	WT	97.5 (*n*=28,985)
*tnSi6* ^‡^ *; fog-2(oz40)*	WT	97.9 (*n*=27,149)
*fog-2(oz40)*	WT	99.4 (*n*=32,760)
*fog-2(oz40)*	*tnSi10* ^§^ *; him-5(e1490)*	100 (*n*>5000)
*oops-1(tn1898); fog-2(oz40)*	*tnSi10* ^§^ *; him-5(e1490)*	0 (*n*>5000)
*fog-2(oz40)*	*tnSi16* ^¶^ *; him-5(e1490)*	100 (*n*>5000)
*oops-1(tn1898); fog-2(oz40)*	*tnSi16* ^¶^ *; him-5(e1490)*	0.5 (*n*=∼28,180)

*tnSi5[mex-5p::mScarlet::spe-11::spe-11 3′UTR].

^‡^*tnSi6[mex-5p::mScarlet::spe-11::spe-11 3′UTR]*.

^§^*tnSi10[spe-11p::GFP::oops-1a::spe-11 3′UTR]*.

^¶^*tnSi16[spe-11p::GFP::oops-1b::spe-11 3′UTR]*.

We were also able to rescue *spe-11(tn2059)* hermaphrodites to fertility through the expression of GFP::SPE-11 in oocytes using the *oma-1*, *oma-2*, *rme-2* and *puf-5* promoters and their respective 3′UTRs (single-copy insertions: *tnSi21*, *tnSi23*, *tnSi25* and *tnSi27*, respectively), further providing evidence that provision of SPE-11 by the oocyte is sufficient (Fig. S8). In each of these cases (e.g. *oma-1*, *oma-2*, *rme-2* and *puf-5*), we observed cortical localization of GFP::SPE-11 in oocytes, but this cortical localization was less apparent in the distal germline, where the endogenous proteins (e.g. OMA-1, OMA-2, RME-2 and PUF-5) are not abundantly expressed (Fig. S8C-F). When GFP::SPE-11 was expressed under control of the *oma-1* and *oma-2* promoters, and their respective 3′UTRs (in *tnSi21* and *tnSi23*, respectively), GFP::SPE-11 was expressed at high levels in oocytes and, in addition to cortical localization, we observed nuclear localization (Fig. S8C,D). In these instances, binding to OOPS-1 may be saturated allowing unbound GFP::SPE-11 to localize to the nucleus, as we observed for the expression of mScarlet::SPE-11 in *oops-1(tn1898)* null mutants (Fig. 5E,F). When GFP::SPE-11 was expressed under control of the *oma-1*, *rme-2* and *puf-5* promoters and respective 3′UTRs, we observed weak expression in sperm, which might contribute to the rescue of fertility in the *spe-11(tn2059)* null mutant background (Fig. S8C,E,F). Because this weak sperm expression was not observed when GFP::SPE-11 was expressed under control of the *oma-2* promoter and 3′UTR (*tnSi23*), we analyzed function in a *tnSi23; fog-2(oz40)* female background by mating with *spe-11(tn2059)* males, which resulted in fertility (Fig. S8G-I), further showing that SPE-11 can be provided by the oocyte.

To determine whether OOPS-1 can function when delivered by the sperm, we generated single-copy insertions to express GFP::OOPS-1A (*tnSi10*) or GFP::OOPS-1B (*tnSi16*) in the male germline under control of the *spe-11* promoter and 3′UTR (Fig. 5A). We tested the ability of *tnSi10* and *tnSi16* males to rescue *oops-1(tn1898); fog-2(oz40)* females upon mating and observed no rescuing activity for *tnSi10* and only very weak activity for *tnSi16* (∼0.5% embryonic viability; Table 4). In addition to using the *spe-11* promoter to express OOPS-1A in sperm (Fig. 5G,H), we used the sperm-specific *peel-1* and *trp-3* promoters and their respective 3′UTR elements to express GFP::OOPS-1A in sperm (*tnSi12* and *tnSi14*, respectively) but were unable to rescue *oops-1(tn1898)* mutants to fertility (data not shown).

### Protein domains required for OOPS-1 and SPE-11 function

**Fig. 6.**
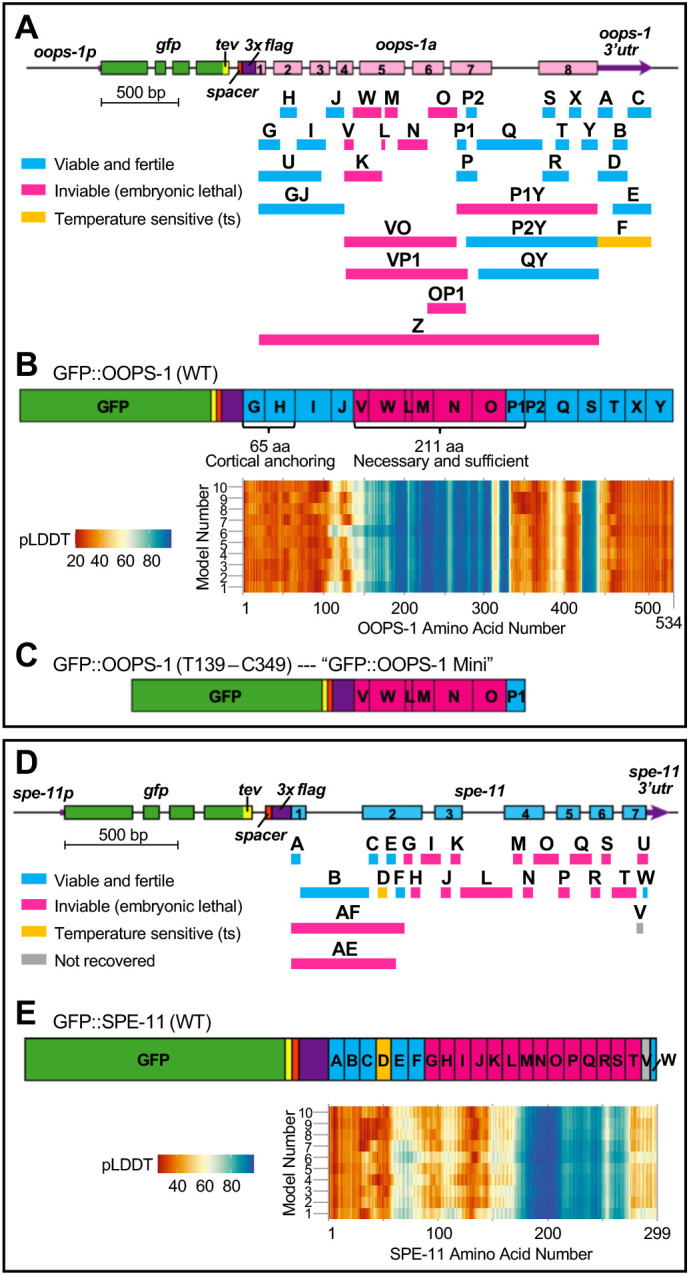
**Protein domains required for OOPS-1 and SPE-11 function.** (A,D) Deletions made by CRISPR-Cas9 genome editing in the context of *oops-1(tn1908[gfp::tev::3xflag::oops-1a])* (A) and *spe-11(tn2094[gfp::tev::3xflag::spe-11])* (D)*.* (B,E) Essential and non-essential regions of OOPS-1 (B) and SPE-11 (E) mapped onto a diagram of each protein. Binary structures for the OOPS-1 and SPE-11 complex were modeled using the AlphaFold 3 server (Fig. S9). The pLDDT values for the C_α_ for each amino acid were plotted in a heatmap. (C) GFP::OOPS-1 Mini made by combining N-terminal and C-terminal deletions confers fertility and viability.

To determine the protein regions essential for OOPS-1 and SPE-11 function, we used CRISPR-Cas9 genome editing to generate in-frame deletions in the context of N-terminally tagged GFP::OOPS-1 and GFP::SPE-11 (Fig. 6). This analysis identified a 211-aa region from the central portion of OOPS-1 as being required for function (Fig. 6A,B). We combined 5′ and 3′ deletions to generate a strain expressing GFP::OOPS-1(T139-C349) Mini (Fig. 6C), which was viable and fertile, indicating that this central region of OOPS-1 is necessary and sufficient for function; however, GFP::OOPS-1 Mini was expressed at high levels in the germline (Fig. S7I,J). For SPE-11, multiple regions were required for function (Fig. 6D,E).

Individually, OOPS-1 and SPE-11 are predicted by AlphaFold to be largely unstructured. IDPs have frequently been found to undergo a disorder-to-order transition upon binding their partners (Trivedi and Nagarajaram, 2022). We used the AlphaFold 3 server to generate a binary model complex; each protein in the complex is predicted to exhibit structurally ordered regions with high per residue measures of local confidence using the predicted local distance difference test (pLDDT; Fig. S9). Remarkably, the regions of high pLDDT values in the complex, which occur where AlphaFold 3 predicts the two proteins to interact, corresponded well to the essential regions of OOPS-1 and SPE-11 (Fig. 6B,E).

### SPE-11 genetically interacts with components of the EGG complex

For SPE-11, we observed that a 14 amino acid deletion (deletion D in Fig. 6D) confers temperature sensitivity with an average brood size of 2±3 (*n*=43) at 25°C. This mutation, hereafter referred to as *spe-11(*ts*)*, might disrupt interactions with other proteins needed for post-fertilization development. Thus, we conducted a genetic selection for dominant suppressors of *spe-11(*ts*)* and isolated mutations that substantially increase fertility (Table 5 and Fig. S10). Since the *spe-11(*ts*)* allele is marked by *gfp*, we verified that the suppressor mutations do not alter GFP::SPE-11(ts) expression levels (data not shown). Seven of these rare dominant suppressors were backcrossed to remove unlinked mutations to facilitate mutation identification using whole-genome sequencing (WGS; Table S4). These mutations define at least three loci (*chs-1*, *gsp-3* and *egg-3*; Table 5). Four additional mutations in *chs-1* and one *egg-3* mutation were identified by genetic mapping and Sanger sequencing. The isolation of an allele of *gsp-3* as a *spe-11(*ts*)* suppressor is consistent with the identification of GSP-3/4 as a protein found in OOPS-1 and SPE-11 TAPs (Table 3). The P195S mutation in *gsp-3(tn2202)* affects an amino acid that is conserved in the human PP1B (PPP1CB) homolog and is predicted to be in a surface-exposed loop (Fig. S11). CHS-1, chitin synthase, is activated upon fertilization to generate the chitin layer of the eggshell (Zhang et al., 2005; Olson et al., 2012; Stein and Golden, 2018), which fails to form in *oops-1* and *spe-11* mutants. CHS-1 and EGG-3 are required for the formation of polar bodies and share mutant phenotypes with *spe-11* and *oops-1* (Maruyama et al., 2007; Johnston et al., 2006, 2010; González et al., 2018; Kawasaki et al., 2024). The suppressor mutations in CHS-1 are predicted to be intracellular (Fig. S12). The strong suppression of *spe-11(*ts*)* by the *chs-1* mutations (Fig. S10) suggests they might restore interactions with a defective SPE-11. The two *egg-3* suppressor mutations affect adjacent amino acids, predicted to localize to a surface-exposed loop (Fig. S13).

**Table 5.** Dominant *spe-11(*ts*)* suppressor mutations

Dominant suppressor loci*	Function	Amino acid substitutions (alleles)^‡^
*chs-1*	Chitin synthase, required for eggshell formation, the block to polyspermy, and the completion of meiosis	S347F(*tn2248*), R645K(*tn2195*), **L669F(*tn2201*)**, A684V(*tn2242*), **A827T(*tn2191*)**, **A1114V(*tn2189, tn2198, tn2210*)**, R1308C(*tn2193*)
*gsp-3*	Sperm-specific PP1 phosphatase	**P195S(*tn2202*)**
*egg-3*	EGG-3 required for eggshell formation, the block to polyspermy, and the completion of meiosis	**P197S(*tn2205*)**, G198R(*tn2190*)

*26 additional suppressor mutations have not been assigned.

^‡^Mutations shown in bold font were ascertained by WGS and Sanger sequencing (Table S4). Mutations shown in regular font were ascertained by Sanger sequencing.

### OOPS-1 and SPE-11 are phosphoproteins

**Fig. 7.**
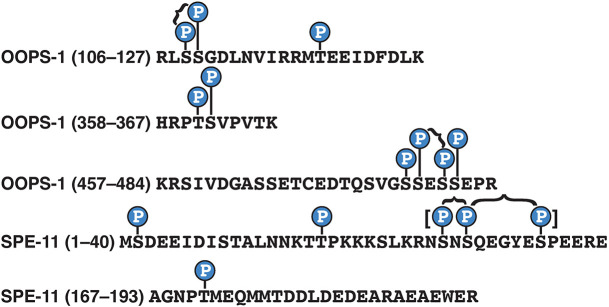
**OOPS-1 and SPE-11 are phosphoproteins.** High-confidence phosphorylation sites identified by mass spectrometry in OOPS-1 and SPE-11, supported by multiple diagnostic b- and y-type fragment ions. Double (braces) and triple (brackets) phosphorylated sites are indicated. Mass spectra supporting these phosphorylations are accessible from the Dryad Digital Repository (Greenstein, 2025; dryad.931zcrjxk).

The suppression of *spe-11(*ts*)* by a dominant *gsp-3* mutation suggests that the GSP-3/4 phosphatases might promote the function of the OOPS-1–SPE-11 complex. Although there are many potential forms such a regulatory mechanism might take, we considered the possibility that the OOPS-1–SPE-11 complex might be controlled by protein phosphorylation. Thus, we examined the mass spectrometry spectra from the TAP experiments for instances of protein phosphorylation. We found multiple examples in which phosphorylation was supported at high-confidence levels by multiple diagnostic b- and y-type fragment ions in the mass spectra (Fig. 7; Greenstein, 2025). These phosphorylation sites are in non-essential regions of both proteins (Fig. 6), suggesting they might define regulatory modifications.

### The OOPS-1–SPE-11 complex functions as an actin regulator *in vitro*

**Fig. 8.**
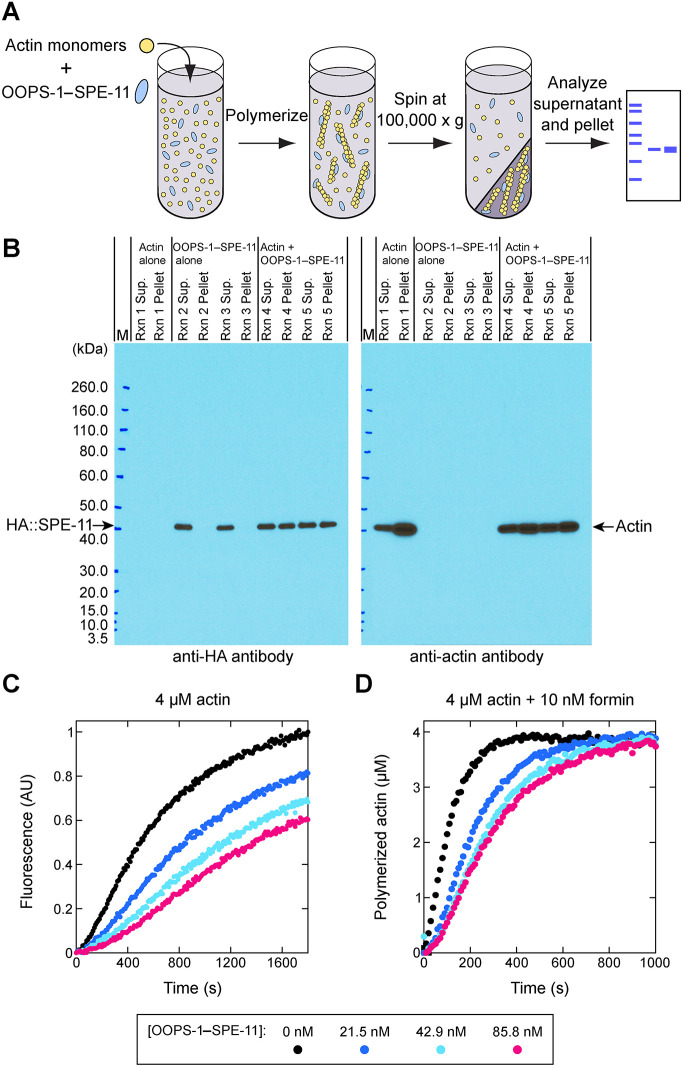
**Biochemical evidence that the OOPS-1–SPE-11 complex is an actin regulator.** (A,B) The OOPS-1–SPE-11 complex interacts with F-actin in the absence of other proteins. (A) Schematic of the co-sedimentation assay used to assess the interaction of the OOPS-1–SPE-11 complex with F-actin. (B) Western blot analyses of the supernatant and pellet fractions. Filaments were assembled from 4 µM actin monomers in the presence or absence of 223 nM OOPS-1–SPE-11 complex. Blots representative of two replicates. M, marker. (C) The OOPS-1–SPE-11 complex slows actin polymerization. Representative time courses of spontaneous polymerization of 4 µM actin (20% pyrene labeled) in the presence of a range of concentrations of OOPS-1–SPE-11. (D) The OOPS-1–SPE-11 complex inhibits formin-mediated actin assembly. Time courses of polymerization of 4 µM actin (20% pyrene labeled) in the presence of 10 nM formin and a range of concentrations of OOPS-1–SPE-11. Fluorescence data were normalized and are represented in units of polymerized actin, which facilitates quantification of polymerization rates. *n*=2.

*spe-11* and *oops-1* mutant embryos are defective in meiotic cytokinesis and exhibit similarities to embryos treated with the actin inhibitor latrunculin A (Yang et al., 2003; McNally and McNally, 2005). Thus, we investigated whether the purified complex interacts with filamentous actin (F-actin; Fig. 8). We initiated actin polymerization in the presence and absence of the OOPS-1–SPE-11 complex and separated F-actin from globular actin (G-actin) by centrifugation at 100,000 ***g*** (Fig. 8A,B). Ordinarily, the OOPS-1–SPE-11 complex localized to the supernatant fraction after centrifugation (Fig. 8B, duplicate reactions 2 and 3); however, when F-actin was present, the complex co-sedimented with F-actin (Fig. 8B, reactions 4 and 5). This result suggests that the OOPS-1–SPE-11 complex binds F-actin in the absence of other proteins. We also observed a reduction in the concentration of pelleted actin in the presence of the complex (Fig. 8B, compare reaction 1 with reactions 4 and 5), consistent with the possibility that the complex decreases the rate of spontaneous actin polymerization, potentially by sequestering actin monomers.

To examine the rate of spontaneous actin polymerization, we monitored the time course of actin assembly in the presence of a range of concentrations of the OOPS-1–SPE-11 complex *in vitro* (Fig. 8C). In these assays, filament nucleation is the primary determinant of the polymerization rate. We observed a concentration-dependent decrease in the rate of actin assembly in the presence of the OOPS-1–SPE-11 complex. Reactions containing OOPS-1–SPE-11 also attained lower final fluorescence values than did the reaction containing actin alone. Collectively, these results suggest that the OOPS-1–SPE-11 complex either slows filament nucleation, decreases the total concentration of F-actin that can be obtained through polymerization, or both.

To dissect the mechanism by which the complex influences polymerization, we examined the effects of the OOPS-1–SPE-11 complex on F-actin assembly in the presence of a constitutively active formin. Formins stimulate polymerization by speeding filament nucleation and regulating filament elongation. In bulk assembly assays, the nucleation activity of formin gives rise to a dramatic increase in the rate of actin polymerization (Pruyne et al., 2002; Pring et al., 2003). Consistent with this activity, formin-mediated actin assembly attained equilibrium within 5 min in the absence of the OOPS-1–SPE-11 complex (Fig. 8D). Inclusion of the complex in the reactions slowed actin assembly in a dose-dependent fashion. In contrast, the fluorescence signal measured at the end of polymerization was unaffected by inclusion of OOPS-1–SPE-11. This supports a mechanism in which the complex inhibits filament nucleation rather than decreasing the total concentration of polymerized actin generated in the reaction. Application of a linear fit to the time courses at the point where half of the actin is polymerized revealed a ∼60% decrease in the rate of filament nucleation at the highest concentration of complex we sampled. Remarkably, inhibition of actin polymerization occurred at sub-stoichiometric concentrations of the OOPS-1–SPE-11 complex: ∼0.6-2% of the concentration of actin monomers. Thus, inhibition cannot involve ‘monomer sequestration’, but might occur through direct binding of actin nuclei, as these species are present at low concentrations throughout the polymerization reaction. These observations indicate that the complex can function as a potent actin regulator, even when present at limiting concentrations.

## DISCUSSION

In many organisms, early development is under maternal control. By contrast, the mechanisms by which the sperm promotes embryonic development, beyond contributing a haploid genome and a centriole pair, are less understood. Here, we address the molecular mechanisms of one of the few strict paternal-effect genes known, *C. elegans spe-11*. We show that SPE-11 functions with an oocyte protein, OOPS-1, in a protein complex that is required for completion of oocyte meiosis, meiotic cytokinesis, synthesis of the eggshell, the block to polyspermy, and the embryonic divisions. The paternal and maternal genetic requirements for *spe-11* and *oops-1*, respectively, indicate that the protein complex likely forms at fertilization. There are relatively few reported examples of sperm–oocyte protein partnerships that play essential intracellular roles in egg activation (Ataeian et al., 2016; this work). The majority of sperm–oocyte protein partnerships defined thus far mediate gamete recognition, binding, and fusion reactions required for fertilization (reviewed by Deneke and Pauli, 2021).

OOPS-1 and SPE-11 are IDPs that appear to be restricted to Caenorhabditid nematodes. This observation is unsurprising because virtually all reproductive proteins are sexually selected, and, as stated by Wilburn and Swanson (2016), ‘the most interesting examples are likely those that directly bind molecules derived from the other sex’. A single sperm, with ∼1/10,000th the volume of an oocyte, deposits a payload of SPE-11, which must rapidly interact with OOPS-1 to execute meiosis at the anterior cortex. Evolution must have selected for a high-affinity interaction because the eggshell begins to form approximately 5 min after fertilization and oocyte meiosis is completed approximately 30 min after fertilization (Ward and Carrel, 1979; McCarter et al., 1999). Indeed, our TAP experiments efficiently recovered the protein complex even though these proteins appear to be found in the same cell only after fertilization. IDPs can exhibit diffusion-limited binding (Borgia et al., 2018), which might be a conserved aspect of fertilization. The purification of the complex from *E. coli* demonstrates that the two proteins can interact in the absence of other *C. elegans* proteins.

**Fig. 9.**
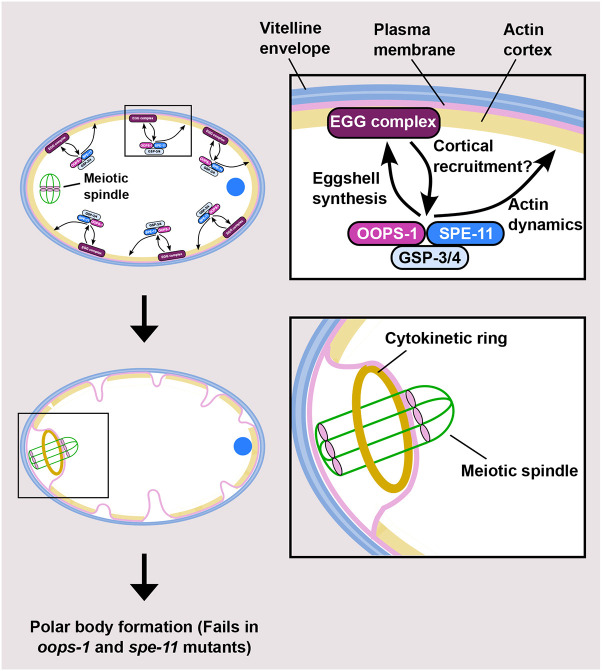
**Model for OOPS-1–SPE-11 complex functions in egg activation.** OOPS-1 and SPE-11 interact upon fertilization. Genetic results suggest the EGG complex might recruit the OOPS-1–SPE-11 to the cortex for the completion of oocyte meiosis. In turn, the OOPS-1–SPE-11 complex is required for synthesis of the chitin layer of the eggshell. Biochemical data indicate that the OOPS-1–SPE-11 complex can function as an actin regulator. Cortical actin dynamics have been proposed to play an important role in meiotic cytokinesis (see Discussion). In this model, OOPS-1–SPE-11 would contribute to actin dynamics along the whole circumference of the embryo, which would contribute to the actin-based process of meiotic cytokinesis. We cannot rule out more spatially localized activities for the complex as SPE-11 localization has not yet been visualized after fertilization.

In the context of the *C. elegans* embryo, interactions with other proteins might facilitate the formation or function of the OOPS-1–SPE-11 complex. Consistent with this possibility, we recovered dominant alleles of *chs-1* chitin synthase and *egg-3* in a screen for suppressors of a *spe-11(*ts*)* mutation. CHS-1 and EGG-3 are required for the formation of polar bodies and share mutant phenotypes with *spe-11* and *oops-1*, including defects in the completion of oocyte meiosis and eggshell synthesis. The fact that disruptions in eggshell synthesis perturb polar body formation was interesting because it was hard to imagine how events happening external to the plasma membrane might affect meiotic cytokinesis on the other side – the chitin layer of the eggshell is deposited external to the plasma membrane just beneath the vitelline layer (Stein and Golden, 2018). Chitin synthases are localized to the plasma membrane and act as channels that export the chitin polymer across the plasma membrane (Ren et al., 2022). Our findings suggest a model in which CHS-1, together with other EGG complex components, cortically recruit the OOPS-1–SPE-11 complex to regulate actin dynamics for the completion of meiosis (Fig. 9). In turn, the OOPS-1–SPE-11 complex might activate CHS-1 for eggshell synthesis (Fig. 9).

Biochemical experiments suggest that the OOPS-1–SPE-11 complex can function as an actin regulator: the OOPS-1–SPE-11 complex interacts with F-actin in the absence of other proteins and appears to inhibit actin nucleation. These data suggest that the OOPS-1–SPE-11 complex possesses an elemental biochemical function at the level of the actin filament. These biochemical experiments were motivated partly by the fact that meiotic cytokinesis is an actin-based process, which enables the asymmetric division that forms small polar bodies and a large embryo.

Although many studies have focused on the role of microtubules and cell-cycle regulation in the control of oocyte meiosis, results in diverse species highlight the importance of dynamic actin networks (Duan and Sun, 2019; Santella et al., 2020). In humans, the meiotic functions of actin prevent aneuploidy, and maternal age-dependent decline in actin function contributes to infertility (Dunkley et al., 2022). In mice, meiotic spindle positioning and cytokinesis require formin 2 and spire actin nucleators to generate a dynamic cytoplasmic actin meshwork (Azoury et al., 2008; Li et al., 2008; Schuh and Ellenberg, 2008; Pfender et al., 2011; Montaville et al., 2014). In starfish, transport of chromosomes to the meiotic spindle depends on actin dynamics (Bun et al., 2018; Burdyniuk et al., 2018).

In *C. elegans*, actomyosin dynamics in the oocyte are triggered at the onset of meiotic maturation (Yan et al., 2022). Following fertilization, actomyosin contractility and cortical destabilization drive membrane ingressions at the cortex during the meiotic divisions (Willis et al., 2006; Fabritius et al., 2011; Schlientz and Bowerman, 2020; Quiogue et al., 2023). This cortical contractility has been proposed to provide the force that pushes the spindle through the actomyosin-free center of the constricting and ingressing contractile ring (Fabritius et al., 2011). Meiotic cytokinesis, which is defective in *spe-11* and *oops-1* mutants, depends on spatially and temporally regulated actin dynamics, with a highly controlled balance of assembly and disassembly. Based on our biochemical results, we suggest that the OOPS-1–SPE-11 complex plays a role in generating cortical actin dynamics through inhibition of actin nucleation (Fig. 9). Consistent with this model, prior work suggests that F-actin destabilization at the cortex plays a role in generating the membrane ingressions because semi-dominant, temperature-sensitive mutations in *act-2*, which affect the actin ATP-binding site, cause abnormal membrane ingressions and incompletely penetrant defects in meiosis (Willis et al., 2006).

Several observations suggest that the activity of the OOPS-1–SPE-11 complex might be highly regulated. Ectopic expression of SPE-11 in the female germline results in colocalization with OOPS-1 to the cortex of germ cells and oocytes without producing detrimental consequences. In fact, the complex retains its function, which is only manifested after oocyte meiotic maturation and fertilization. If our biochemical experiments have predictive value, the expectation might be that ectopic formation of the complex would result in cortical destabilization in the germline, which is not observed. One possibility is that the activity of the complex is confined to the period following oocyte meiotic maturation and fertilization, potentially by protein kinases that are activated during this developmental time window. Indeed, both OOPS-1 and SPE-11 are phosphoproteins with multiple modification sites. These sites localize to non-essential regions of the two proteins, which can be deleted without phenotypic consequences, consistent with the possibility that they might be regulatory. Potentially, some modifications could be inhibitory and others activating.

A surprising finding, first shown by Browning and Strome (1996) and validated here at a quantitative level using the new tools that have become available in the interim, is that SPE-11 can mediate its function when provided by the oocyte. Why then has nature gone to the trouble of separating the complex into gametes of opposite sex? Current data provide few clues and fewer answers, so we can only speculate. It is notable that OOPS-1 is most abundantly expressed in the distal germline where it does not appear to have a required role because *oops-1* null mutants are maternal-effect lethal. Whether *oops-1* has a non-essential or redundant role will require additional analysis. If it does, perhaps SPE-11 might interfere with this function through its high-affinity interaction with OOPS-1. Alternatively, the separation of OOPS-1 and SPE-11 into gametes of opposite sex might relate to the fact that female meiosis in *C. elegans* is under multiple paternal controls: the MSP hormone promotes oocyte meiotic maturation (Miller et al., 2001); SPE-11 promotes meiotic progression and meiotic cytokinesis; and sperm factors, including the GSP-3/4 PP1 phosphatases and the GSKL-1/2 glycogen synthase kinase homologs, promote meiosis II after fertilization (Ataeian et al., 2016; Banerjee and Srayko, 2022). Perhaps this regulation ensures fertilization and prevents parthenogenetic development, which is a derived reproductive strategy in many nematodes.

### Limitations

The sexual reproduction of all animals depends on fertilization. Here, we define a molecular interaction between two intracellular proteins, the oocyte protein OOPS-1 and the sperm protein SPE-11, that is required for multiple aspects of early post-fertilization development, including the completion of meiosis and eggshell formation. Yet several questions remain about the dynamics of protein complex formation and the mechanistic aspects of complex function *in vivo*. Using standard microscopic methods (e.g. widefield and confocal microscopy) and endogenously tagged fluorescent proteins, we were unable to visualize SPE-11 post-fertilization. Examination of the dynamics of the complex after fertilization will require methods with single-molecule sensitivity to image SPE-11 from the single sperm that fertilizes the much larger oocyte. Our biochemical studies show that the OOPS-1–SPE-11 complex binds actin and inhibits actin nucleation *in vitro*. How this biochemical activity contributes at a mechanistic level to the completion of meiosis in the wild type, and its failure in *oops-1* and *spe-11* mutants, will require dynamic studies of the actin cytoskeleton *in utero* during the period in which the eggshell is being synthesized and the newly fertilized embryos are osmotically sensitive.

## MATERIALS AND METHODS

### Strains, genetic analysis, phenotypic analysis and WGS for mutant identification

The genotypes of strains used in this study are reported in Table S5. Genes and mutations are described in WormBase (Sternberg et al., 2024) or in the indicated references. Culture and genetic manipulations were conducted at 20°C as described (Brenner, 1974), except for the analysis of *spe-11(tn2094 tn2145*ts*)* or its suppressors, which were conducted at 15°C or 25°C, and RNA interference (RNAi), which was conducted at 22°C. The balancer chromosomes (Dejima et al., 2018) used for *spe-11* and *oops-1* were *tmC18[dpy-5(tmIs1236)]* I and *tmC25[unc-5(tmIs1241)]* IV, respectively. RNAi was performed by feeding with double-stranded RNA (dsRNA)-expressing *E. coli* (Timmons and Fire, 1998) using the culture media described by Govindan et al. (2006). The RNAi clone (I-2C18) targeting *mat-1*, which encodes the anaphase-promoting complex CDC27/APC3 subunit (Davis et al., 2002), was obtained from Source BioScience (Nottingham, UK), and its identity was validated by Sanger sequencing. Exposure to *mat-1(RNAi)* was initiated during the fourth larval stage.

To isolate dominant mutations able to suppress the *spe-11(tn2094 tn2145*ts*)* mutation, L4-stage hermaphrodites were mutagenized with 50 mM ethyl methanesulfonate at 15°C. One or two mutagenized hermaphrodites were cultured on 100 mm×15 mm or 150 mm×15 mm Petri dishes with nematode growth medium (Brenner, 1974) or peptone-enriched nematode growth medium at 15°C, with bacterial strains OP50-1 or NA22 as the food source. The F1 progeny were transferred to 25°C 3-5 days later and suppressed strains were isolated by their ability to produce progeny efficiently over multiple generations at 25°C. These rare dominant suppressors were sought in the F1 generation by screening approximately ∼1.5×10^6^ ethyl methanesulfonate-mutagenized genomes. Suppressed strains were outcrossed seven times using *tmC18[dpy-5(tmIs1236)]/+* males for WGS.

Genomic DNA was prepared for WGS using the QIAGEN DNeasy Blood and Tissue Kit. Illumina libraries of genomic DNA were prepared and sequenced by Azenta GENEWIZ to approximately 100× coverage. As controls, the N2 strain, the DG5649 parent strain and the FX30168 *tmC18* strain were also sequenced to filter out differences between the reference genome and our laboratory versions, as well as variants introduced during backcrossing. Reads were trimmed using TrimGalore (v.0.6.0) and mapped to the BSgenome.Celegans.UCSC.ce11 (v.1.4.2) genome using BWA mem (v.0.7.17-r1188). Aligned reads were sorted with Samtools (v.1.16.1) and duplicates were identified using MarkDuplicates (Picard; v.2.18.16). The GATK Haplotype caller (v.4.1.2.0) was used to identify sequence variations with respect to the reference ce11 genome. The VariantAnnotation package (v.1.50.0) within R (v.4.4.0) was used to filter variants for homozygous, single-nucleotide polymorphisms with read depth ≥15 that were not present in all samples. These variants were annotated using the TxDb.Celegans.UCSC.ce11.ensGene (v.3.15.0) annotation package to consider all splice donor/acceptor mutations and non-synonymous protein coding mutations.

The following mutations were found to exhibit linkage to chromosome I, as they were balanced by *tmC18*: *chs-1(tn2189, tn2191, tn2193, tn2195, tn2198, tn2201, tn2210, tn2242* and *tn2248)* and *gsp-3(tn2202)*. These mutations have not been separated from *spe-11(tn2094 tn2145*ts*)*. *egg-3(tn2190* and *tn2205)* were found to exhibit linkage to chromosome II, as they were balanced by *mnC1[dpy-10(e128) unc-52(e444) umnIs32]*. The two *egg-3* alleles were separated from *spe-11(tn2094 tn2145*ts*)* and found to be viable and fertile at 25°C. The suppressor mutations were isolated in the F1 generation, suggesting they were dominant. To explicitly test dominance for *chs-1(tn2189, tn2191, tn2198, tn2201* and *tn2210)* and *gsp-3(tn2202)*, *spe-11(tn2094 tn2145*ts*)/tmC18; tnEx265[str-1::gfp]* males were crossed to the suppressed (sup) strains at 25°C [genotypes: *sup spe-11(tn2094 tn2145*ts*)*] and hermaphrodites of genotypes *sup/+ spe-11(tn2094 tn2145* ts*); tnEx265* were examined at 25°C and found to be fertile, indicating dominance. For *egg-3(tn2190* and *tn2205)*, adult hermaphrodites of genotype *egg-3(tn2190* or *tn2205)/mnC1[umnIs32]*; *spe-11(tn2094 tn2145*ts*)* were generated at 25°C and found to exhibit suppression (Fig. S10).

### Genome editing

All alleles of *oops-1* and *spe-11* with a *tn* allele designation described in this work were generated by CRISPR-Cas9 genome editing at the endogenous loci. All edited loci were validated by sequencing the repair junctions using PCR products as templates. sgRNA plasmids and repair templates used to generate deletions in *oops-1* and *spe-11* are described in Table S6. Plasmids expressing single-guide RNAs (sgRNAs) under the control of the U6 promoter were generated as described (Arribere et al., 2014). Repair templates used to tag *oops-1* and *spe-11* with *gfp* were also generated as described (Dickinson et al., 2015). Repair templates used to tag *spe-11* with mSCARLET-I were as described (Spike et al., 2022). Genome editing was performed by injecting wild-type adult gonads with a DNA mix containing a repair template (10 ng/µl), one or more sgRNA plasmids (25 ng/µl each), Cas9-expressing plasmid (pDD162; 50 ng/µl) and injection marker (pMyo2::tdTomato; 4 ng/µl) and selecting for repairs and self-excising cassette (SEC) excisions using standard methods (Dickinson et al., 2015). Each repair was balanced and the SEC was removed from heterozygotes. Fluorescently tagged *oops-1 and spe-11* alleles were homozygous fertile after SEC excision. Deletions were constructed using the *dpy-10* co-conversion method (Arribere et al., 2014). The injection mix contained pJA58 (7.5 ng/μl), AF-ZF-827 (500 nM), the appropriate sgRNA plasmid (25 ng/μl), the appropriate repair template (500 nM) and pDD162 (50 ng/μl).

To express mScarlet::SPE-11 or GFP::SPE-11 in the female germline and GFP::OOPS-1 in the male germline, single-copy insertions (designated as *tnSi* alleles) were generated by combining Mos1-mediatred single-copy insertion (MosSCI; Frøkjær-Jensen et al., 2008, 2014) and CRISPR/Cas9 approaches. Promoters, fluorescently tagged *oops-1* or *spe-11*, and 3′UTR regions were amplified from genomic DNA by PCR using the oligonucleotides listed in Table S6. Amplified fragments were inserted into pKL129 (kind gift from the Caenorhabditis Genetics Center) by Gibson assembly using NEBuilder HiFi DNA Assembly Master Mix (New England Biolabs) to generate the MosSCI plasmids, which also contained a rescuing copy of *unc-119* (Table S6). To generate single-copy insertion strains, *ttTi5605* II; *unc-119(ed3)* III adults from EG6699 (Frøkjær-Jensen et al., 2012) were injected with an injection mix containing MosSCI plasmids (50 ng/μl), a co-injection marker (pMyo2::tdTomato; 4 ng/µl), and two sgRNA plasmids pXW7.01 and pXW7.02 (50 ng/μl each; kind gift from Ekaterina Voronina, University of Montana, MT, USA), which were used to excise the *ttTi5605 Mos1* transposon on chromosome II. Injected animals were cultured at 22°C for approximately 7 days, after which *unc-119*-rescued F2 progeny not containing the pMyo2::tdTomato co-injection marker were individually isolated and their progeny screened for stable transmission. These progeny were crossed with males heterozygous for the *mnC1[dpy-10(e128) unc-52(e444) umnIs32]* balancer chromosome, and offspring that did not segregate *unc-119(ed3)* were identified by PCR using a cleaved amplified polymorphic sequence marker (Table S6), designed using dCAPS Finder 2.0 (Neff et al., 2002). Correct targeting was verified by conducting PCR and by Sanger sequencing. Oligonucleotides used as PCR primers or for Sanger sequencing are listed in Table S6.

Endogenous fluorescent reporter tag insertions for EGFP::CHS-1 were conducted as described (Vicencio et al., 2019). The injection mix contained Cas9 protein (250 ng/µl), universal tracrRNA (141.97 ng/µl), *dpy-10* crRNA (14.39 ng/µl), allele-specific crRNA (59 ng/µl), *dpy-10* repair oligonucleotide (28 ng/µl) and allele-specific oligonucleotide (116 ng/µl). All reagents were obtained from Integrated DNA technologies, Inc. Guide and repair templates used to generate insertions are described in Table S6. Each injection mix was injected into the germline of wild-type young adult hermaphrodites and the F1 generation screened with a combination of single-worm propagation and lysis followed by detection of gene deletions or tag insertions by PCR. Each new edited strain was verified by Sanger sequencing. Oligonucleotides used as PCR primers or for Sanger sequencing are listed in Table S6.

### Microscopy

#### Widefield microscopy

For the images in Fig. 1B,C, FM4-64 and Calcofluor were utilized to examine eggshell permeability and chitin formation, respectively. L4-stage hermaphrodites were placed onto fresh MYOB media and cultured for 24 h. Embryos were dissected in egg buffer [4 mM HEPES (pH 7.4), 94 mM NaCl, 3.2 mM KCl, 2.7 mM CaCl_2_ and 2.7 mM MgCl_2_] supplemented with 16 mM of FM4-64 (Bai et al., 2020) or 1:5 ratio of Calcofluor:egg buffer, respectively. Embryos were dissected in 5 µl of supplemented egg buffer on a cover slip using a scalpel. To prevent pressure on the embryos, a depression microscope slide was fixed to the coverslip using four droplets of Vaseline on the edges. Images were acquired on a Zeiss AxioObserver inverted widefield microscope using a 40× Plan-Neofluar (numerical aperture 1.3) objective lens and Axiocam 503 camera (Carl Zeiss Inc.). Image processing and analysis were conducted using Zen Microscopy (Carl Zeiss Inc.) and ImageJ/Fiji software (Schindelin et al., 2012). All images were obtained using identical parameters, with brightness and contrast adjusted for better visualization.

For the images in Fig. 2 and the movies, live imaging of oocyte meiosis was conducted on a Zeiss AxioObserver inverted widefield microscope using a 100× Plan-Apochromat (numerical aperture 1.4) objective lens and an Axiocam 503 camera (Carl Zeiss Inc.). A microscope slide with a 2% agarose pad was prepared with 40 µl of 2 mM tetramisole. Adult hermaphrodites were placed and immobilized in the tetramisole and covered with a coverslip. For each sample, meiotic events were imaged in a single focal plane with a time lapse of 30 s intervals.

The microscopy images in Figs 4A-D, 5B-H and Figs S2B, S3, S7 and S8 were acquired on a Nikon Ni-E microscope with either a Plan Apo λ 60× (numerical aperture 1.4) objective or a Plan Fluor 40× Oil (numerical aperture 1.3) objective using an ORCA FLASH sCMOS camera (Hamamatsu) and NIS elements software (Nikon Inc.). The images in Fig. S2A,C,D were acquired on a Zeiss motorized Axioplan 2 microscope with a 40× Plan-Neofluar (numerical aperture 1.3) objective lens using an AxioCam MRm camera and Zeiss AxioVision software.

#### Confocal microscopy

Localization and image analysis for Fig. 1D-F were conducted on an Andor Dragonfly spinning disk confocal microscope (Oxford Instruments) using a Plan Apo 63× objective lens (numerical aperture 1.47) and a Zyla sCMOS camera (Oxford Instruments). A microscope slide with a 2% agarose pad was prepared with 40 µl of 20 mM tetramisole. Young adult worms were placed and immobilized in the tetramisole and covered with a coverslip. Germlines were imaged using *z*-stack projections of a constant 0.5 µm per slice. Image processing and analysis were conducted using Imaris image analysis software (Oxford Instruments) and ImageJ (Fiji). All images were obtained using identical parameters, with brightness and contrast adjusted for better visualization.

The microscopy images in Fig. 4E,F were acquired on a Nikon Ti2 inverted confocal microscope with a Plan ApoIR 60× objective (numerical aperture 1.27), motorized stage and Galvano scanner using a DUG hybrid four-channel detector system that combines gallium arsenide phosphide and multi-alkali photomultiplier tubes and NIS elements software (Nikon Inc.).

### Proteomics

Tandem affinity purification of OOPS-1 and SPE-11 was conducted using strains DG4800, DG5430 and DG5462 using modifications of a previously described protocol (Tsukamoto et al., 2017, 2020). The first immunopurification used a mixture of anti-GFP monoclonal antibodies 12A6 and 4C9 (Developmental Studies Hybridoma Bank, University of Iowa) and the second immunopurification used anti-FLAG monoclonal antibody M2 (Sigma-Aldrich). Immunopurified proteins were precipitated with 16.7% trichloroacetic acid, washed with acetone at −20°C, and separated on a 12% NuPAGE Bis-Tris gel, stained with Colloidal Blue Staining Kit (Invitrogen). Lanes were subdivided into eight gel slices. The excised gel slices were subjected to in-gel trypsin proteolytic digestion as described previously (Thu et al., 2016) with the following change. During the alkylation step, 55 mM iodoacetamide was used instead of 55 mM methyl methanethiosulfonate. Post-digestion, the peptides in each gel band were purified with a C18 Stage tip (Rappsilber et al., 2003). Eluates were vacuum-dried*.* Mass spectrometry for Experiment I was performed at the Taplin Biological Mass Spectrometry Facility (Harvard Medical School, MA, USA) using an LTQ Orbitrap Velos Pro ion-trap mass spectrometer (Thermo Fisher Scientific, Inc.). Experiments II-VII were conducted at the Center for Metabolomics and Proteomics at the University of Minnesota. Experiments II and III utilized an Orbitrap Fusion liquid chromatography mass spectrometer (Thermo Fisher Scientific, Inc.). Experiments IV-VII used an Orbitrap Eclipse liquid chromatography mass spectrometer. The tandem mass spectrometry data were processed using Sequest (Eng et al., 1994). The *Caenorhabditis elegans* Universal Proteome UP000001940 protein sequence database was downloaded from UniProt and merged with a common laboratory contaminant protein database (Frankenfield et al., 2022). We applied a 1% protein and peptide false discovery rate using the Percolator algorithm (Käll et al., 2007). For phosphoprotein analysis, Experiments II-VII were reanalyzed using Proteome Discoverer 3.0 using phosphoserine, phosphothreonine and phosphotyrosine in the database search parameters. Tables S1 and S2 report additional technical details of the proteomic analyses, and the peptides identified in the OOPS-1 and SPE-11 immunopurifications, respectively. High-confidence phosphorylation sites identified in the OOPS-1 and SPE-11 immunopurifications, based on multiple diagnostic b- and y-type fragment ions, were identified by inspection of the mass spectra. The mass spectral data are accessible from the Dryad Digital Repository (Greenstein, 2025; dryad.931zcrjxk).

### Expression and purification of the OOPS-1–SPE-11 complex

#### Expression plasmid construction

The expression plasmid for production of tagged versions of OOPS-1 and SPE-11 in *E. coli* was constructed as shown in Fig. S14, and the oligonucleotide primers used are listed in Table S6*. oops-1* cDNA (TT767) and *spe-11* cDNA (TT768) codon optimized for *E. coli* were commercially synthesized using gBlock Hi-Fi (IDT) and were used as templates for PCR to amplify cDNA fragments encoding tagged version of OOPS-1 and SPE-11. Q5 DNA polymerase (New England Biolabs) and the primer pair of TT769/TT770 and the pair of TT771/TT772 were used to amplify *3xFLAG::TEV::oops-1a* and *S-tag::HA::PreScission::spe-11*, respectively. These PCR products were digested with appropriate restriction enzymes and were gel-purified using QIAprep 2.0 spin columns (QIAGEN). Purified PCR fragments were ligated into the pRSFDuet-1 vector (Novagen) between EcoRI and HindIII sites of the first multiple cloning site, and between NdeI and AvrII sites of the second multiple cloning site, respectively, (pTT197 and pTT204 plasmids) using T4 DNA ligase (New England Biolabs). *S-tag::HA::PreScission::spe-11* fragment was excised from pTT204 by NdeI and AvrII digestion and was inserted into pTT197 to generate pTT207, which contains both *6xHis::3xFLAG::TEV::oops-1* and *S-tag::HA::PreScission::spe-11*. To replace the 6xHis tag of OOPS-1 with a HaloTag, HaloTag cDNA fragment was amplified by PCR using the primer pair of TT909 and TT910 and was inserted between NcoI and HindIII digestion sites of pTT219 plasmid. The plasmids were transformed into DH5α *E. coli* cells and their DNA sequences were confirmed by Sanger sequencing.

#### Protein induction and purification

Expression of proteins was carried out in BL21-AI *E. coli* cells (Promega). The transformed cells were cultured at 30°C in LB medium containing 50 µg/ml of kanamycin until an OD_600_ of 0.4-0.6. OOPS-1 and SPE-11 proteins were induced at 30°C for 4 h by addition of 1 mM IPTG and 0.2% L-arabinose. Cells were harvested by centrifugation at 3500* **g*** for 20 min at 4°C, and proteins were extracted using Bacterial Protein Extraction Reagent (B-PER; Thermo Fisher Scientific) containing EDTA-free Halt protease inhibitors (Thermo Fisher Scientific), 5 units/ml of DNase I (Thermo Fisher Scientific) and 100 µg/ml of lysozyme (Thermo Fisher Scientific). The cell lysate was centrifuged at 20,000 ***g*** for 15 min at 4°C, and supernatant was used for protein purification.

For the primary purification, the supernatant was diluted with an equal volume of TBSN buffer (50 mM Tris-HCl, pH 7.4, 150 mM NaCl, 0.05% NP-40) and applied to an ANTI-FLAG M2 affinity gel (Sigma-Aldrich) in batches for 3 h at 4°C. After the binding, the affinity gel was loaded onto a column and was washed with 20 column volumes of TBSN buffer. The bound proteins were eluted from the column by competitive elution with three column volumes of 150 μg/ml 3x FLAG peptide (Sigma-Aldrich) in TBSN buffer.

For the secondary purification, the eluate from the ANTI-FLAG M2 affinity gel was diluted with an equal volume of HaloTag protein purification buffer (50 mM HEPES, pH 7.5, 150 mM NaCl, 0.05% NP-40) and applied to HaloLink resin (Promega) in batch overnight at 4°C plus 1 h at room temperature. After the binding, the unbound proteins were removed by centrifugation at 2500 ***g*** for 5 min at 4°C. The resin was washed with 20 resin volumes of HaloTag protein purification buffer, and bound proteins were cleaved from the HaloLink resin by adding and mixing with the cleavage solution containing 283 units/ml of HaloTEV protease (Promega) in HaloTag protein purification buffer for 1.5 h at room temperature. Supernatant containing the cleaved proteins were collected by centrifugation at 3200 ***g*** for 5 min at 4°C.

For tertiary purification, the proteins were incubated with Pierce Anti-HA Agarose (Thermo Fisher Scientific) for 3 h at 4°C in batches. After the binding, the agarose resin was loaded onto a column and was washed with 20 column volumes of TBSN buffer. The bound proteins were eluted from the column by competitive elution with two column volumes of 1 mg/ml HA peptide (Thermo Fisher Scientific) in TBSN buffer after incubation at 30°C for 30 min. The eluted proteins from anti-HA agarose were dialyzed against KMEI buffer [50 mM KCl, 1 mM MgCl_2_, 1 mM EGTA, 10 mM imidazole (pH 7.0)] using Slide-A-Lyzer dialysis cassettes with 10 kDa molecular weight cut-off (Thermo Fisher Scientific) overnight at 4°C, flash-frozen on powdered dry ice and stored at −80°C. Eluted peptides at each step of purification were analyzed by running on a 4-12% Bis-Tris NuPAGE gel (Invitrogen) and visualized by staining with Colloidal Blue staining kit (Invitrogen).

### Structure modeling and visualization methods

The amino acid sequences for OOPS-1, isoform 1a (Wormbase: CE43675, NCBI Accession: NP_500868) and SPE-11 (Wormbase: CE10744, NCBI Accession: P54217) were entered into the AlphaFold 3 Server (Abramson et al., 2024) to model a complex with one copy of each protein. Random seeds were used to initiate ten different model complexes. AlphaFold 3 generated five models in each run, and the results for the highest-ranked model from each run were analyzed. AlphaFold 3 calculated the confidence level for the position of each atom in the structure using the pLDDT. These values vary from 0 to 100, with values above 90 having high confidence and those below 50 indicating that the structure was likely incorrect. The pLDDT scores from the B-factor field of the mmCIF files were extracted using R Studio v.2024.12.0+467, and the pLDDT values for the alpha carbons (C_α_) for each amino acid were plotted in a heatmap using geom_tile in ggplot2 (Wickham, 2016).

The models for the complexes containing the full-length proteins were visualized using ChimeraX v.1.9 (Meng et al., 2023). The proteins were colored using the pLDDT values ranging from red (low values) to blue (high values). In each model, the N and C termini of each protein were mostly disordered, but the central part of the complex where the proteins were predicted to interact had a consistent structure in each model with reasonably high-confidence levels (pLDDT>70). In addition, the predicted aligned error in this part of the structure was low, suggesting that there was high confidence in the relative positions of the amino acids in the two proteins in this region. This again suggested that the proteins likely interact in this region. The structural models for the suppressor mutants were visualized using ChimeraX v.1.9 as described above. The AlphaFold models for CHS-1 (https://alphafold.ebi.ac.uk/search/text/G5ECD6) and EGG-3 (https://alphafold.ebi.ac.uk/search/text/Q20402) were used. The AlphaFold 3 Server was used to model the structure of GSP-3 (WormBase:CE14754, NCBI accession O02658) with 2 Mn^2+^ ions because crystal structures of homologous mammalian serine/threonine PP1 phosphatases contained two metal ions in the active site (Egloff et al., 1995; Goldberg et al., 1995) that are required for enzyme activity (Zhang et al., 1996).

### Actin biochemistry

#### Purification of actin and formin

Actin was purified from an acetone powder of frozen chicken breast muscle (Trader Joe's, Minneapolis, MN, USA) by one cycle of polymerization and depolymerization (Spudich and Watt, 1971), followed by gel filtration on Sephacryl S-300 resin in G-Buffer (2 mM Tris-HCl, pH 8.0, 0.5 mM ATP, 0.5 mM DTT and 0.1 mM CaCl_2_). Actin monomers were polymerized by dialyzing in 100 mM KCl, 2 mM MgCl_2_, 25 mM Tris-HCl (pH 7.5) and 0.3 mM ATP, and incubated overnight at 4°C with a 1:10 molar ratio of actin to pyrenyl iodoacetamide (P29, Thermo Fisher Scientific). Labeled F-actin was pelleted by ultracentrifugation at 120,000 ***g***, depolymerized, clarified, and gel-filtered in G-Buffer. We used extinction coefficients of 26,000 M^−1^ cm^−1^ at λ=290 nm for unlabeled actin and 22,000 M^−1^ cm^−1^ at λ=344 nm for pyrene, and the following relation to calculate the concentration of pyrene-labeled actin: [total actin]=[A_290_−(A_344_·0.127)] 26,000 M^−1^ cm^−1^.

A construct encoding the FH1 and FH2 domains of the *Saccharomyces cerevisiae* formin Bni1p (residues 1227-1776) was cloned into a pGEX-4T-3 plasmid (GE Healthcare Life Sciences), which was modified to encode an N-terminal TEV protease recognition sequence and a C-terminal 6xHis tag. The protein was expressed overnight at 16°C in a 1-l culture of BL21(DE3) RP Codon Plus cells (Agilent Technologies). Resuspended cell pellets were lysed by sonication, clarified by centrifugation, and incubated with glutathione-Sepharose resin (Gold Biotechnology). The protein was eluted with 100 mM GSH (pH 8.0) in 50 mM Tris (pH 8.0), 100 mM NaCl and 1 mM DTT and incubated with 2-5 µM MBP-tagged TEV protease overnight at 4°C to remove the GST tag. The purified protein was separated from the TEV protease and cleaved GST by nickel affinity chromatography, concentrated using a 30,000 molecular-weight cutoff spin column (EMD Millipore), dialyzed into KMEI buffer with 1 mM DTT, flash-frozen and stored at −80°C. We used ProtParam (http://web.expasy.org/protparam) to calculate the extinction coefficient (Gasteiger et al., 2005).

#### Co-sedimentation assays

Ca^2+^-actin monomers were converted to Mg^2+^-actin by the addition of 0.05 mM MgCl_2_ and 0.2 mM EGTA. Samples containing 4 µM Mg^2+^-actin monomers were polymerized in KMEI buffer for 1 h at 22°C in the absence or presence of 223 nM OOPS-1–SPE-11 complex. Polymerized samples were centrifuged for 30 min at 100,000 ***g***. Supernatants and pellets were separated and analyzed by western blot. The supernatant fraction was first trichloroacetic acid precipitated as described above and resuspended in LDS sample buffer supplemented with a reducing agent (Invitrogen). Equivalent fractions of the supernatant and pellet fractions were separated using NuPAGE 4-12% Bis-Tris gels (Invitrogen) and visualized after western blotting. Blots were blocked with 5% nonfat dried milk. The primary antibodies used were mouse anti-HA.11 monoclonal 16B12 (ENZ-ABS118, Enzo Life Sciences; 1:30,000) and mouse anti-actin monoclonal clone C4 (0869100, MP Biomedicals; 1:10,000). The secondary antibodies used were peroxidase-conjugated goat anti-mouse (115-035-146, Jackson ImmunoResearch Laboratories; 1:100,000). Detection was performed using SuperSignal West Femto Maximum Sensitivity Substrate and CL-XPosure film (Thermo Fisher Scientific).

#### Pyrene-actin assembly assays

Time courses of actin polymerization were collected by measuring fluorescence emission with a Molecular Devices SpectraMax Gemini EM fluorescence plate reader using Corning 96-well flat-bottom plates. Reactions containing 4 µM actin (20% pyrene labeled) and a range of concentrations of OOPS-1–SPE-11 complex were polymerized in the absence or presence of formin in 10 mM imidazole (pH 7.0), 50 mM KCl, 1 mM MgCl_2_, 1 mM EGTA, 0.17 mM ATP, 0.5 mM DTT, 0.03 mM CaCl_2_ and 0.17 mM Tris-HCl (pH 8.0). Samples were excited at 365 nm and the fluorescence emission intensity was measured every 10 s at 407 nm over a period of 30 min. In reactions containing formin, the fluorescence signal was converted to polymer concentration by normalizing the fluorescence intensity to the final predicted actin polymer concentration, assuming a critical concentration of 0.17 µM. Reactions performed in the absence of formin did not attain equilibrium, thus precluding calculations of polymer concentration. The polymerization rate was calculated from the slope of the change in fluorescence signal at the point where half of the actin was polymerized.

## Supplementary Material



10.1242/develop.204674_sup1 Supplementary information

Table S1. OOPS-1-associated proteins (related to Table 3 and Fig. 2).

Table S2. SPE-11-associated proteins (related to Table 3 and Fig. 2).

Table S4. Mutations identified by whole-genome sequencing (WGS) (related to Table 55).

Table S6. Sequence of oligonucleotides used in this study for generation of plasmids, genome editing, PCR, and sequencing.
