# Supplementary material for: A sperm–oocyte protein partnership required for egg activation in Caenorhabditis elegans
Source: Development. 2025 Jun 25;152(12):dev204674. doi: 10.1242/dev.204674 (PMC12273631; doi:10.1242/dev.204674)
Supplement: Supplementary information [file develop-152-204674-s1.pdf]

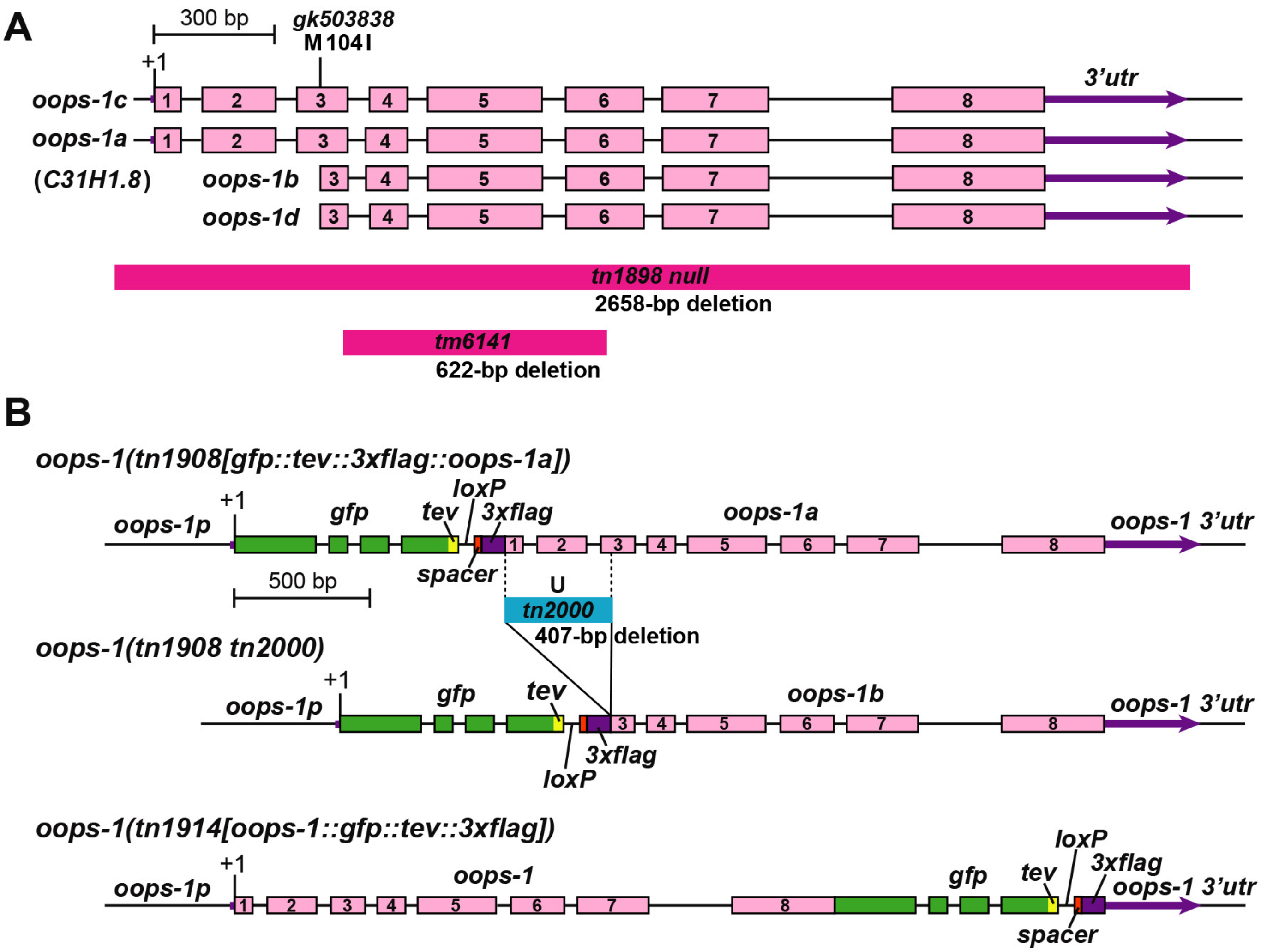

Fig. S1. Mutations and genome edits at the *oops-1* locus. (Related to Fig. 1). (A) Genomic structure of *oops-1* showing several mutant alleles used in this study. (B) N- and C-terminal GFP-tagged alleles of *oops-1* generated by CRISPR-Cas9 genome editing. The *oops-1(tn1908tn2000)* allele removes 407 bp, including exons 1 and 2 and a portion of exon 3, as well as introns 1 and 2, such that only *oops-1b/d* can be expressed from the locus.

Development • Supplementary information

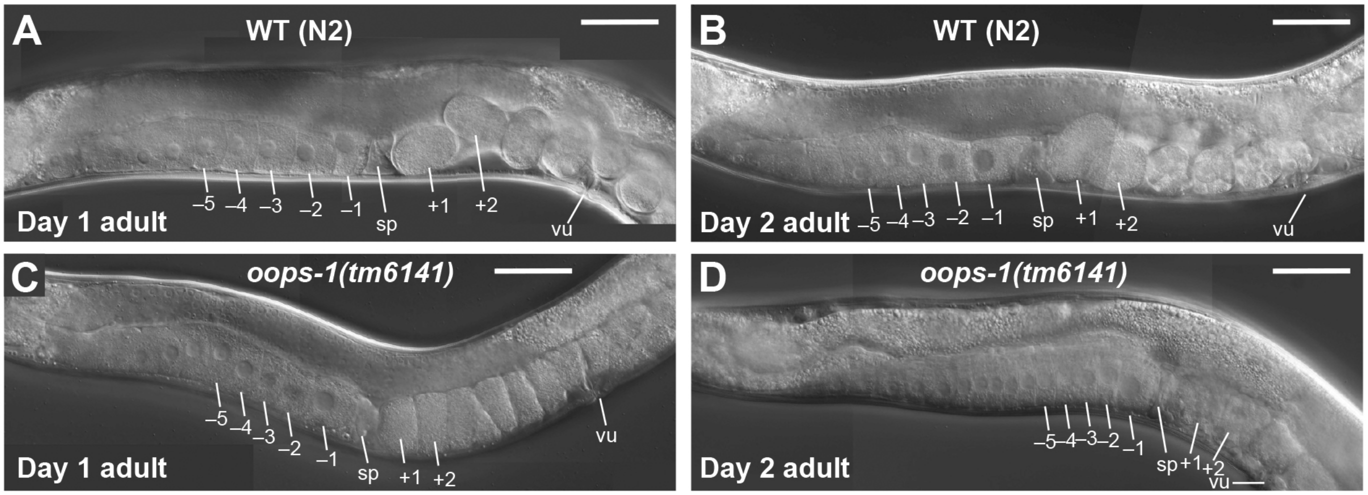

Fig. S2. The one-cell arrest and oocyte-stacking phenotype observed in *oops-1(tm6141)* mutants. (Related to Fig. 1). DIC images of wild-type (A,B) and *oops-1(tm6141)* adult hermaphrodites (C,D) on day 1 (A,C) and day 2 (B,D) of adulthood. Day 1 adult *oops-1(tm6141)* hermaphrodites contain 1-cell arrested embryos in the uterus [e.g., the region between the spermatheca (sp) and the vulva (vu)]. Day 2 adult *oops-1(tm6141)* hermaphrodites contain many arrested oocytes in the gonad arm and fewer embryos in the uterus owing to the depletion of sperm via polyspermy (stacked oocyte phenotype-see Table 1 in the main text). Proximal oocytes (-1 to -5), newly fertilized embryos (+1 and +2), and the vulva (vu) are indicated. Bars, 50 μm.

Development • Supplementary information

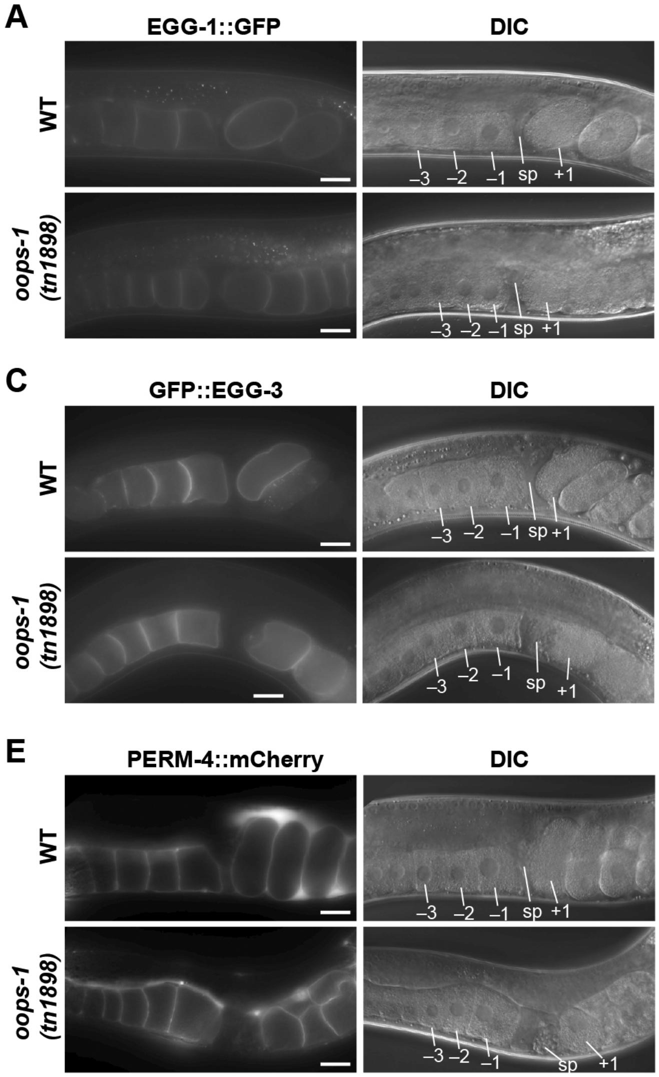

Fig. S3. Normal localization of EGG and PERM complex proteins in *oops-1(tn1898)* null mutant oocytes and embryos. (Related to Fig. 1). (A-E) Day 1 adult hermaphrodites were examined for the expression of EGG-1::GFP (A), EGG-2::GFP (B), GFP::EGG-3 (C), PERM-2::mCherry (D), and PERM-4::mCherry (E) in wild-type and *oops- 1(tn1898)* mutants. Proximal oocytes (-1 to -3), the spermatheca (sp), and newly fertilized embryos (+1) are indicated. Bars, 20µm.

Development • Supplementary information

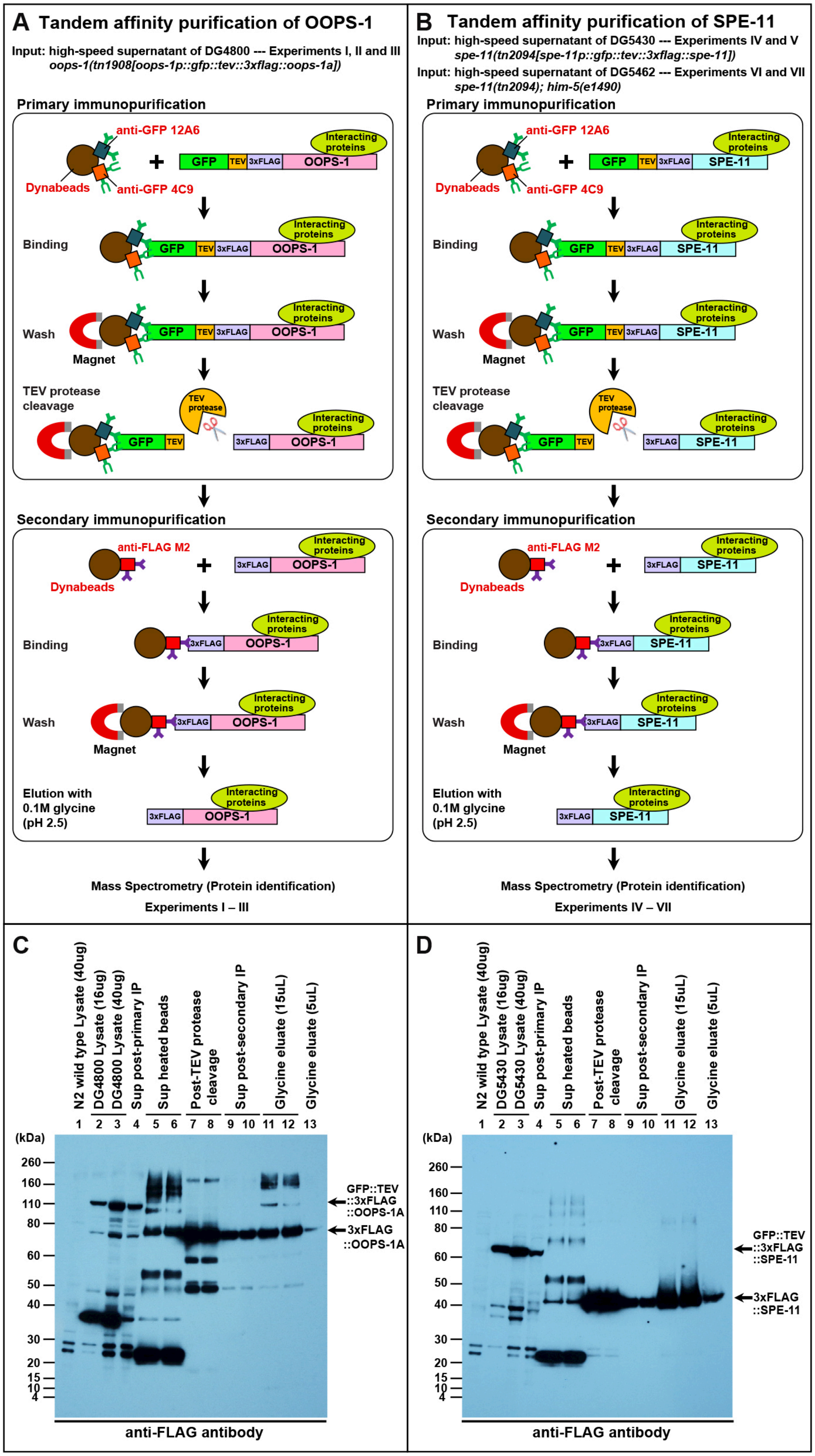

Development • Supplementary information

Fig. S4. Overview and validation of tandem-affinity purification of OOPS-1 and SPE-11. (Related to Table 3, Table S1, and Table S2). Outline of tandem-affinity purification of GFP::TEV::3xFLAG::OOPS-1 (A) and GFP::TEV::3xFLAG::SPE-11 (B). (C, D) Western blots of NuPAGE 4-12% Bis-Tris gels visualized after western blotting in which stages of the GFP::TEV::3xFLAG::OOPS-1 (C) or GFP::TEV::3xFLAG::SPE-11 (D) tandem-affinity purification were monitored. The blots were blocked with 5% nonfat dried milk. Primary antibody used to detect proteins was anti-FLAG monoclonal antibody M2 (1 µg/mL; Sigma-Aldrich). The secondary antibody used for western blot was peroxidase-conjugated goat anti-mouse antibody (1:30,000 dilution; Jackson ImmunoResearch Laboratories). Detection was performed using SuperSignal West Femto Maximum Sensitivity Substrate (Thermo Scientific) with 10 second exposure times. The positions of the full-length proteins in the starting material for the purifications and the resulting products are indicated, as are the positions of the molecular weight markers.

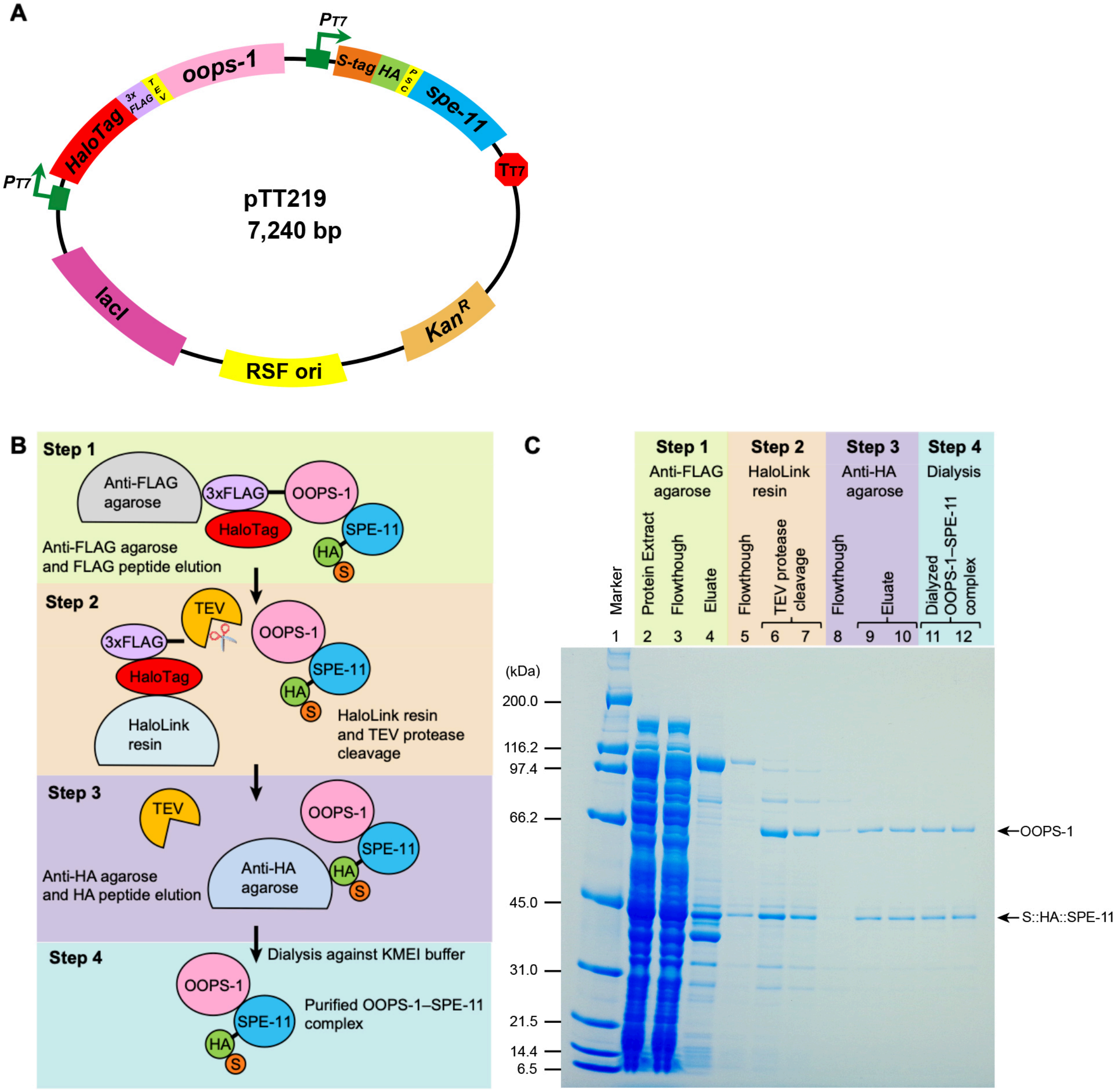

Fig. S5. Interaction of OOPS-1 and SPE-11 in the absence of other *C. elegans* proteins. (Related to Fig. 3). Expression and purification of the OOPS-1-SPE-11 complex. (A) Plasmid for co-expression of tagged versions of OOPS-1 and SPE-11 in *E. coli*. (B) Flow chart of steps used for affinity purification of the complex. (C) Colloidal Coomassie-stained protein gel showing intermediate and final steps of the affinity purification of the complex.

Development • Supplementary information

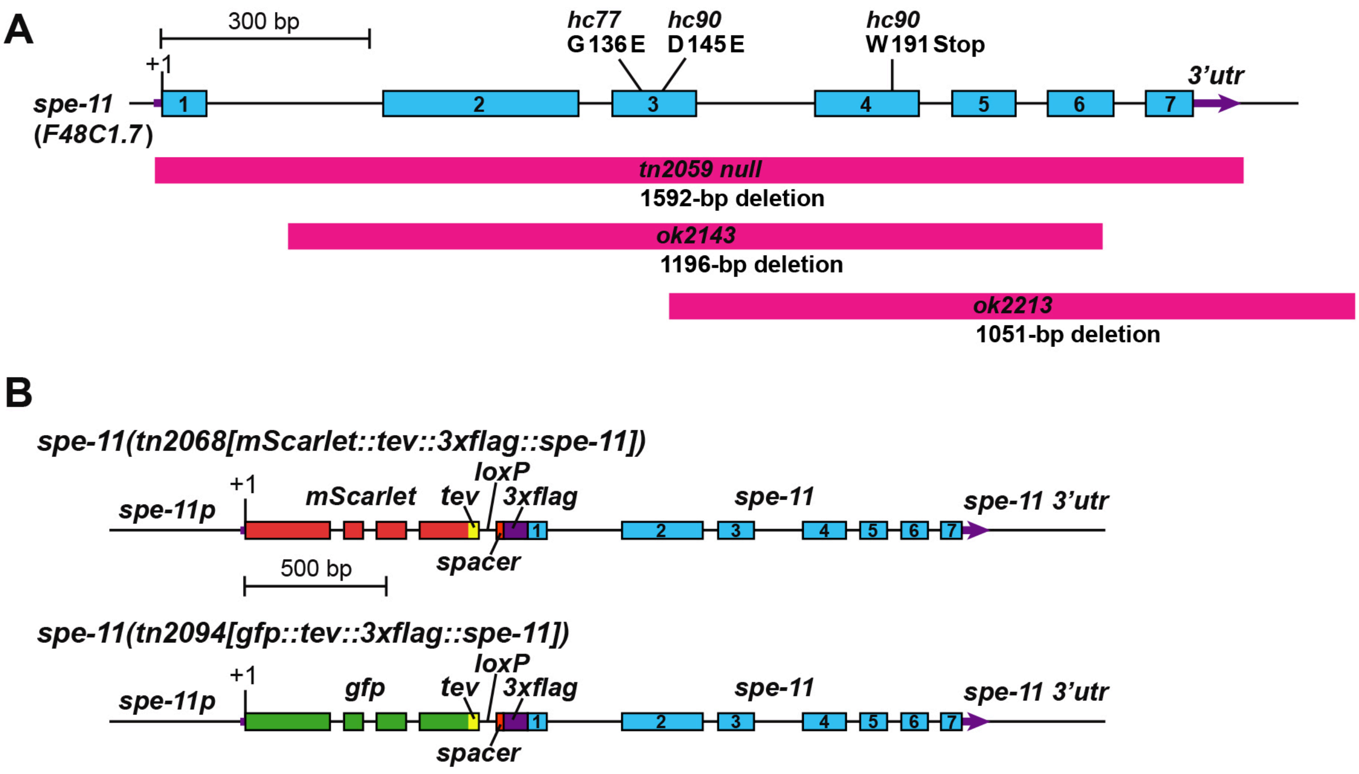

Fig. S6. Mutations and genome edits at the *spe-11* locus. (Related to Figs 2 and 4). (A) Genomic structure of *spe-11* showing several mutant alleles used in this study. (B) Alleles of *spe-11* tagged with mScarlet or GFP generated by CRISPR-Cas9 genome editing.

Development • Supplementary information

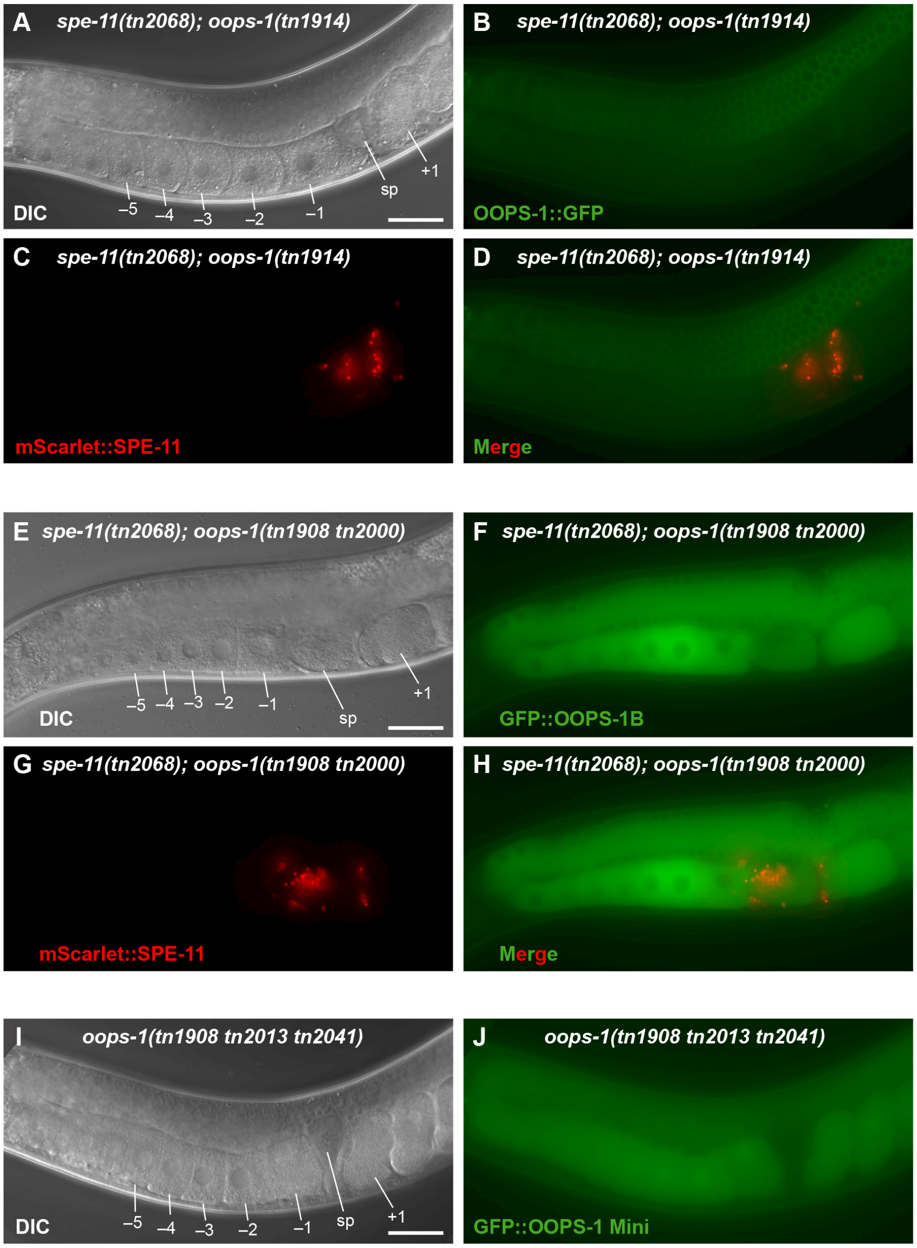

Fig. S7. OOPS-1 and SPE-11 exhibit complementary expression patterns. (Related to Fig. 4). DIC (A) and wide-field fluorescence micrographs (B-D) of a *spe-11(tn2068[mScarlet::tev::3xflag::spe-11]); oops-1(tn1914[oops-1::gfp::tev::3xflag])* adult hermaphrodite (A-D). Tagging at the C-terminus with GFP should label all isoforms. DIC (E) and wide-field fluorescence micrographs (F-H) of a *spe-11(tn2068[mScarlet::tev::3xflag::spe-11]); oops-1(tn1908tn2000[gfp::tev::3xflag::oops-1b/d])* adult hermaphrodite. The deletion in *oops-1(tn1908tn2000)* results in the expression of only OOPS-1B/D isoforms. (I, J) DIC (I) and a wide-field fluorescence micrographs (J) of GFP::OOPS-1(T139-C349) Mini [*oops-l(tnl908tn20l3tn204l)*]. Note that GFP::OOPS-1(T139-C349) Mini exhibits both cytoplasmic and nuclear localization. Identical exposure times were used for the fluorescence images. Proximal oocytes (-1 to -5), spermatheca (sp), and a newly fertilized embryo (+1) are indicated. Bars, 30 μ m.

Development • Supplementary information

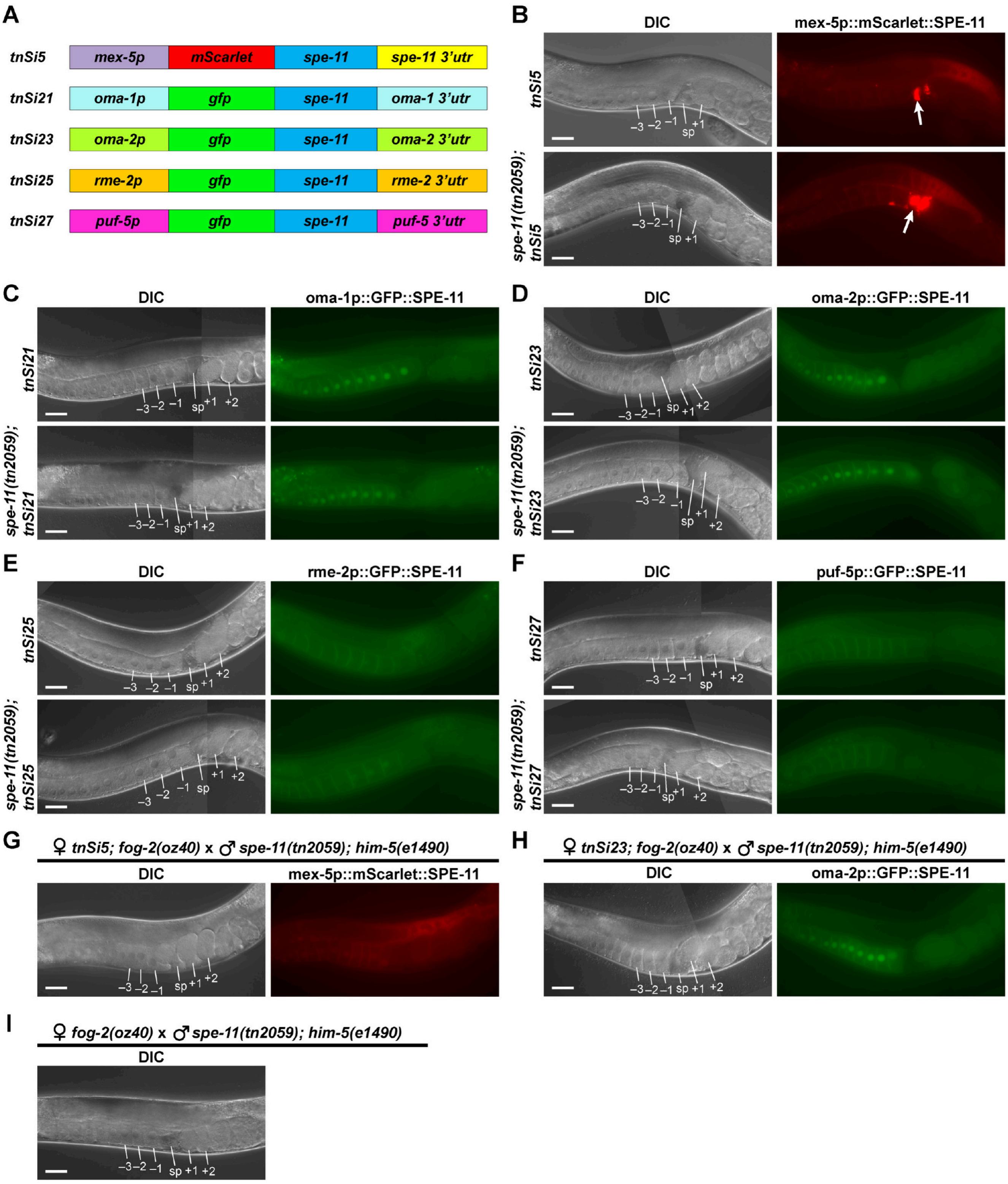

Development • Supplementary information

Fig. S8. Expression of SPE-11 in the female germline complements a *spe-11* null mutation. (Related to Table 4 and Figure 5). Expression of *gfp*::*spe-11* in the female germline rescues *spe-11(tn2059)* null mutants. (A) Single-copy insertions were generated to express mScarlet::SPE-11 or GFP::SPE-11 under control of the indicated promoters and 3'UTR sequences. These promoters and 3'UTR sequences were chosen because they drive expression in the female germline. (B-I) DIC and fluorescence micrographs showing that expression of mScarlet::SPE-11 (B,G) or GFP::SPE-11 (C-F,H) in the female germline can rescue *spe-11(tn2059)* null mutants to fertility. Note, in each case, multicellular embryos are observed in the uterus and the respective hermaphroditic strains are viable and fertile. As a further test, *tnSi5* (the *mex-5* promoter)*; fog-2(oz40)* females (G) or *tnSi23* (the *oma-2* promoter)*; fog-2(oz40)* females (H) were mated with *spe-11(tn2059); him-5(el490)* males and found to be fertile. For, *tnSi5,* in which the *mex-5* promoter and *spe-11* 3'UTR was used, progeny counts are shown in Table 4. Progeny counts were not conducted for *tnSi23*, in which the *oma-2* promoter and 3'UTR were used. Note, when *fog-2(oz40)* females are mated with *spe-11(tn2059); him-5(el490*) males, 1-cell arrested embryos lacking eggshells are observed in the uterus (I). Proximal oocytes (-1 to -3), spermatheca (sp), and a newly fertilized embryo (+1 and +2) are indicated. Arrows indicate sperm in panel B. Bars, 30 µm.

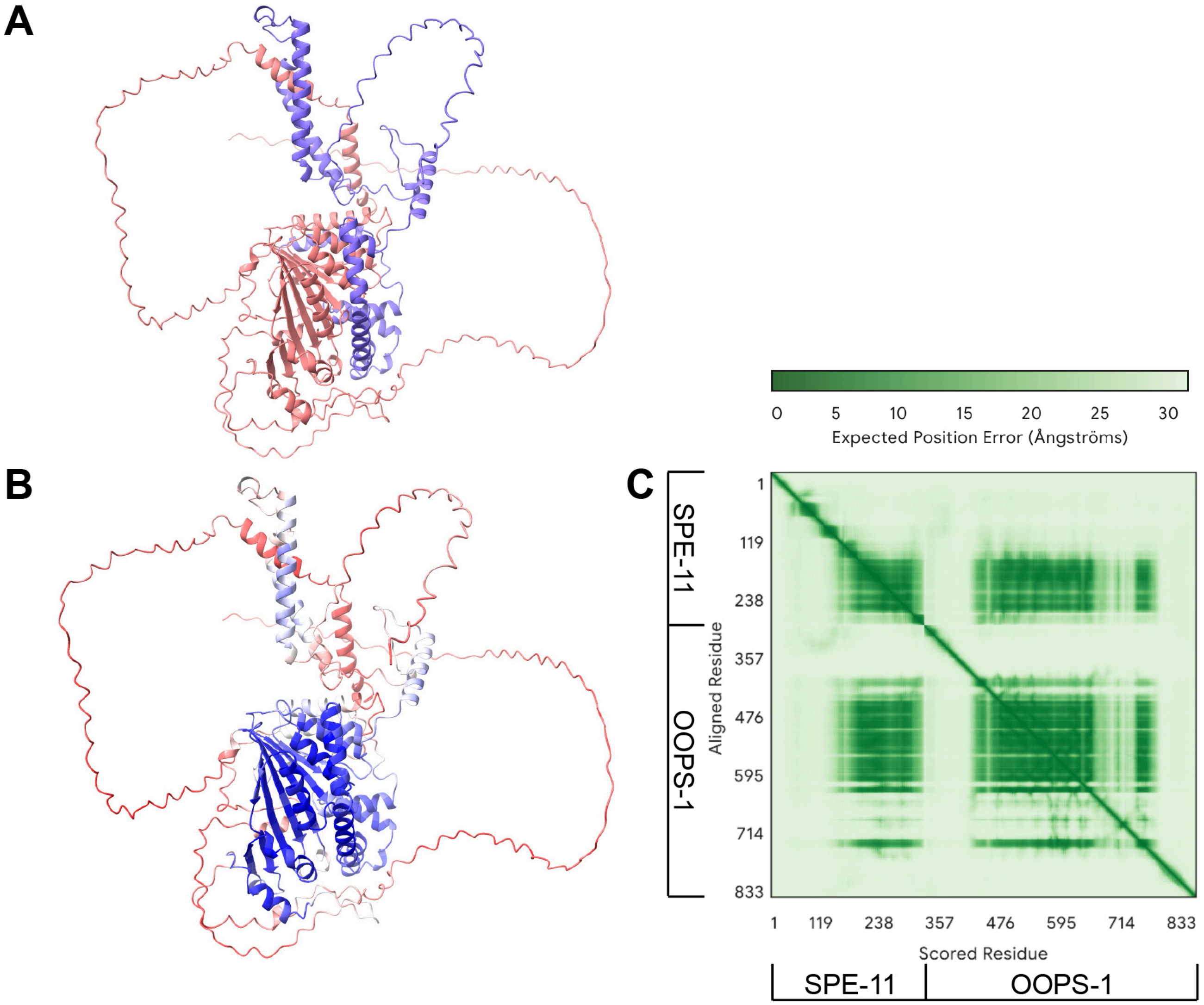

Fig. S9. AlphaFold structural predictions for the OOPS-1–SPE-11 complex. (Related to Fig. 6). Binary structures for the OOPS-1 and SPE-11 complex were modeled using the AlphaFold 3 server. The highest-ranked structure from model 1 is shown and colored based on (A) the protein with OOPS-1 in peach and SPE-11 in purple, and (B) the pLDDT values, with red indicating low values and blue indicating high values. In comparison to the protein structures within the predicted binary complex, Alphafold predicts the individual proteins to be largely unstructured. See https://alphafold.ebi.ac.uk/entry/Q18343 for the predicted OOPS-1 structure and https://alphafold.ebi.ac.uk/entry/P54217 for the predicted SPE-11 structure. (C) Predicted aligned error (PAE) plot for a top-scoring model (model 1) of the OOPS-1-SPE-11 binary complex. PAE is a confidence measure for the relative position of any two residues within the predicted structure.

Development • Supplementary information

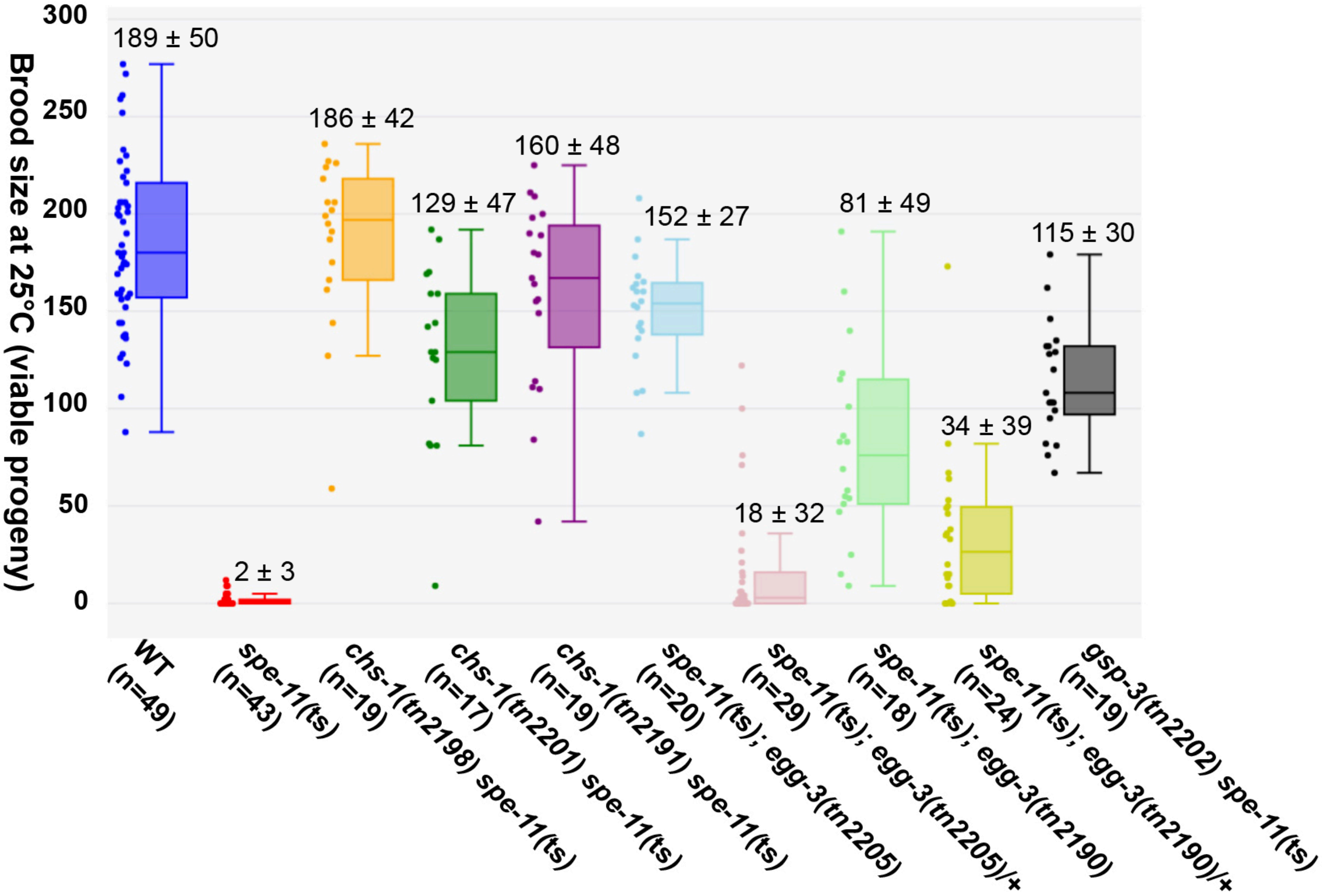

Fig. S10. ***spe-11(*ts*)* suppressor mutations restore fertility.** (Related to Table 5). Brood counts (total viable progeny) of the wild type, *spe-11(*ts*),* and suppressor strains of the genotypes listed. Boxplots, showing means, quartiles calculated using the linear method, and all data points, were constructed using software from http://www.statskingdom.com. The average brood sizes for the suppressed strains were compared to that of *spe-11(*ts*)* using a two-sample t-test with Welch's correction, and in each case p<0.0001, except for *spe-11(ts); egg-3(tn2205)/+*, in which case p=0.0199 (the exact p values are listed in Table S4).

Development • Supplementary information

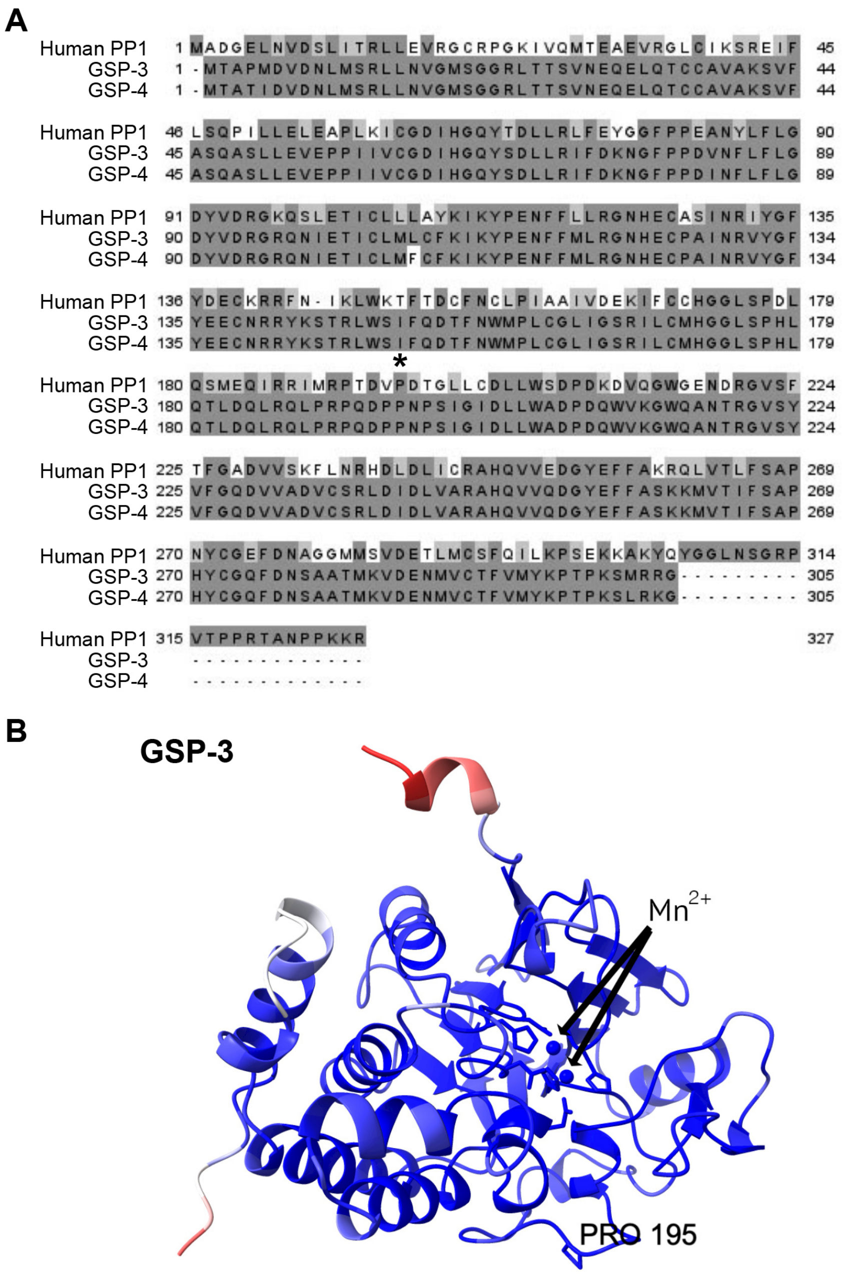

Fig. S11. AlphaFold structural predictions of GSP-3 and the location of a *spe-11*(ts) suppressor mutation. (Related to Table 5). (A) ClustalW alignment of human PP1B and GSP-3 and GSP-4 from *C. elegans*. The position of the P195S mutation in *gsp-3(tn2202)* is indicated by an asterisk. (B) AlphaFold predicted structure of GSP-3 showing the predicted location of P195 and the phosphatase active site and the metals in the phosphatase active site. The coloring is based on pLDDT values.

Development • Supplementary information

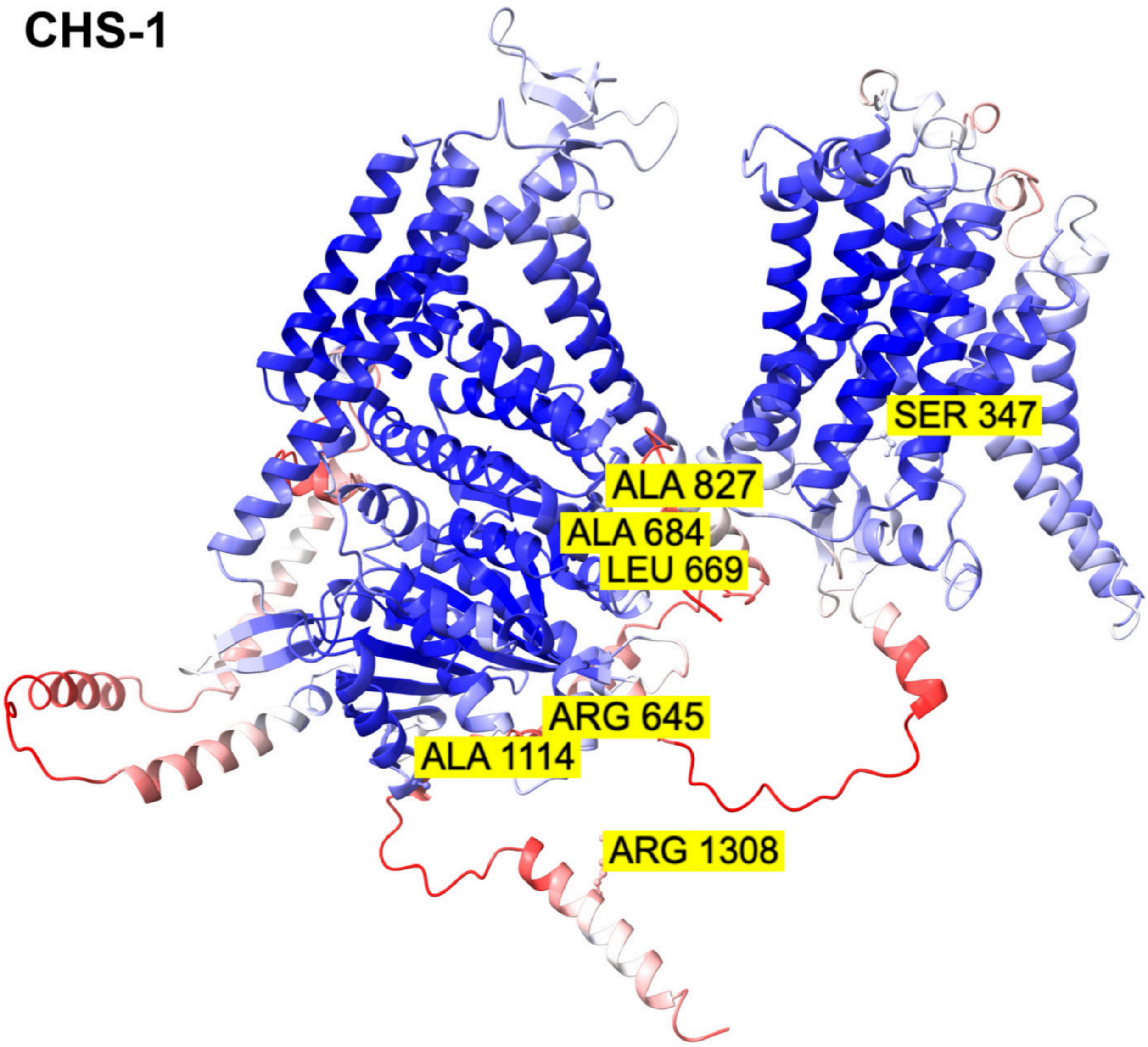

Fig. S12. AlphaFold structural predictions of CHS-1 and the location of *spe-11(*ts*)* suppressor mutations. (Related to Table 5). AlphaFold predicted structure of CHS-1 showing the predicted location of the *spe-11(*ts*)* suppressor mutations. The coloring is based on pLDDT values.

Development • Supplementary information

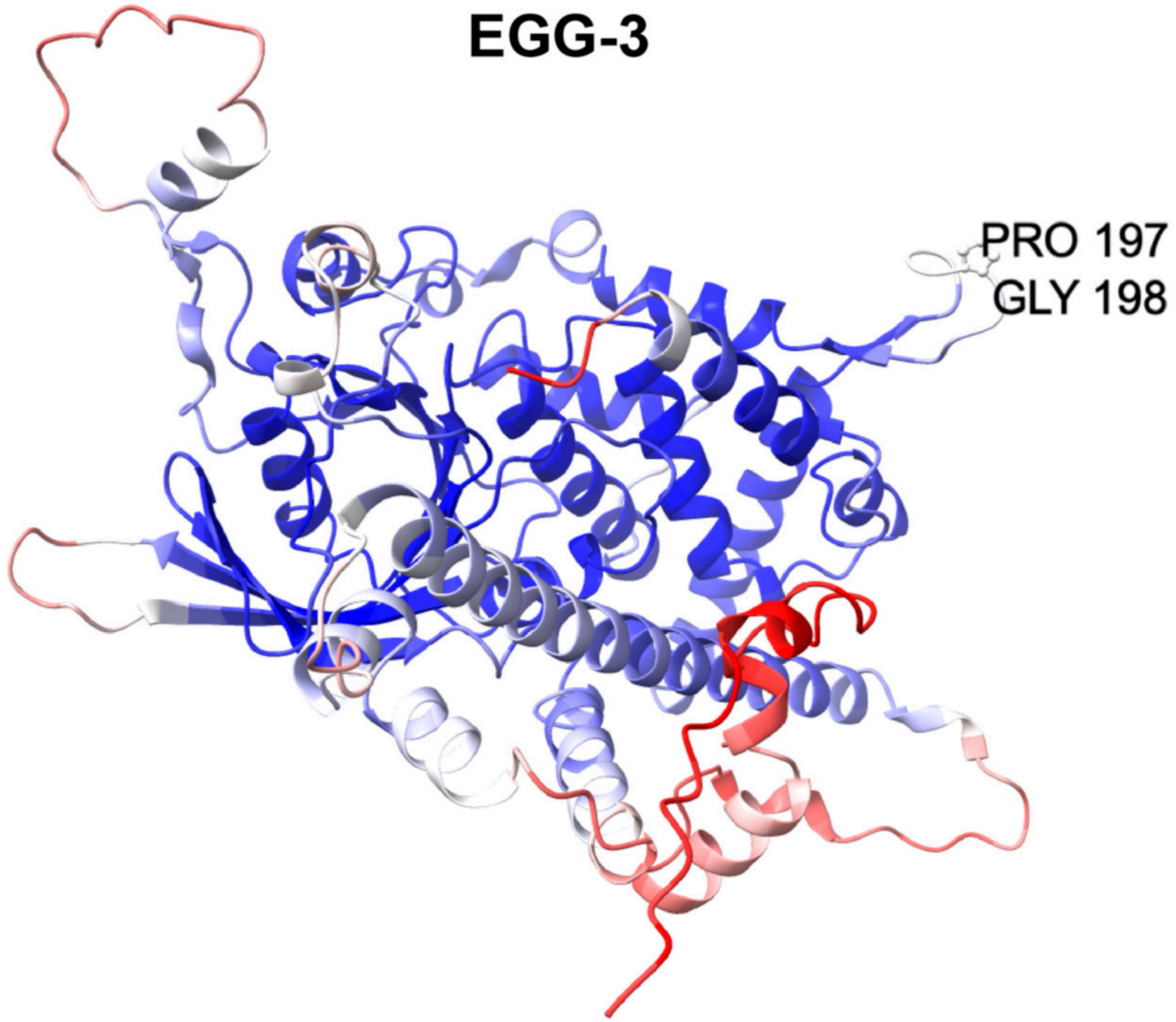

Fig. S13. AlphaFold structural predictions of EGG-3 and the location of *spe-11(*ts*)* suppressor mutations. (Related to Table 5). AlphaFold predicted structure of EGG-3 showing the predicted location of the *spe-11(*ts*)* suppressor mutations. The coloring is based on pLDDT values.

Development • Supplementary information

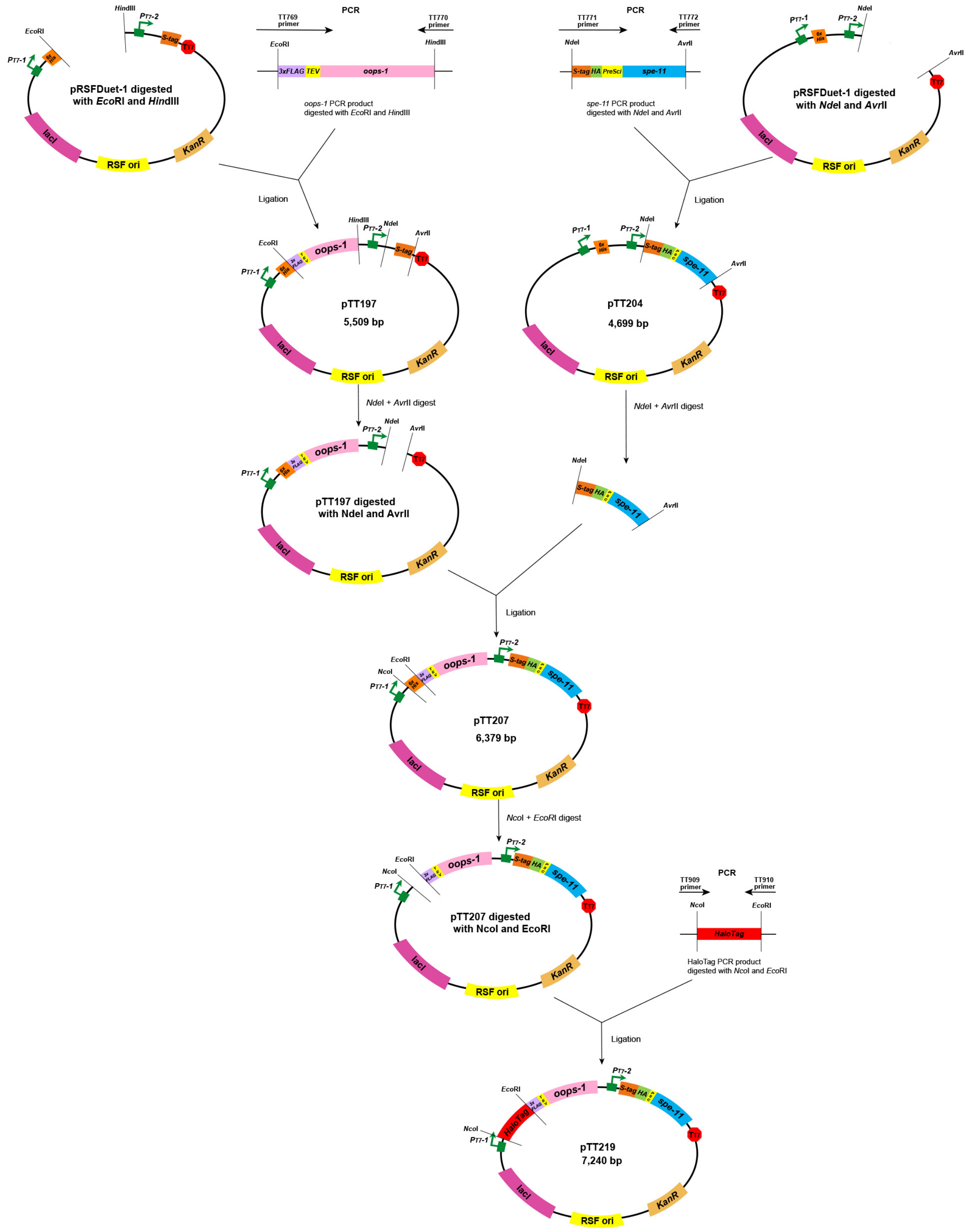

Fig. S14. Overview of plasmid construction for expression of the OOPS-1–SPE-11 complex in *E. coli*. (Related to Fig. 3). Schematic of cloning methods to generate plasmids for expression of tagged versions of OOPS-1 and SPE-11 in *E. coli*.

Development • Supplementary information

Table S1. OOPS-1-associated proteins (related to Table 3 and Fig. 2).

Available for download at
https://journals.biologists.com/dev/article-lookup/doi/10.1242/dev.204674#supplementary-data

Table S2. SPE-11-associated proteins (related to Table 3 and Fig. 2).

Available for download at
https://journals.biologists.com/dev/article-lookup/doi/10.1242/dev.204674#supplementary-data

Table S3. Statistical analysis related to Table 2 using Fisher's exact test

Comparison	Genotypes	N values in phenotypic class	N values in all other classes	Exact *P* value	Odds ratio
		Pre-anaphase I arrest			
1	*oops-1(tn1898)*<br>*spe-11(tn2059)*	13<br>6	28<br>13	1	0.994
2	*oops-1(tn1898)*<br>*spe-11(tn2059); oops-1(tn1898)*	13<br>4	28<br>15	0.5414	0.580
3	*spe-11(tn2059)*<br>*spe-11(tn2059); oops-1(tn1898)*	6<br>4	13<br>15	0.7140	0.586
		Anaphase I arrest			
4	*oops-1(tn1898)*<br>*spe-11(tn2059)*	9<br>2	32<br>17	0.4756	0.424
5	*oops-1(tn1898)*<br>*spe-11(tn2059); oops-1(tn1898*	9<br>7	32<br>12	0.3465	2.047
6	*spe-11(tn2059)*<br>*spe-11(tn2059); oops-1(tn1898)*	2<br>7	17<br>12	0.1245	4.753
		Meiosis II arrest			
7	*oops-1(tn1898)*<br>*spe-11(tn2059)*	3<br>2	38<br>17	0.6477	1.400
8	*oops-1(tn1898)*<br>*spe-11(tn2059); oops-1(tn1898)*	3<br>0	38<br>19	0.5447	0
9	*spe-11(tn2059)*<br>*spe-11(tn2059); oops-1(tn1898)*	2<br>0	17<br>19	0.4864	0
		Meiotic Exit (Completion phenotype—see Fig. 2)			
10	*oops-1(tn1898)*<br>*spe-11(tn2059)*	16<br>9	25<br>10	0.5831	1.400
11	*oops-1(tn1898)*<br>*spe-11(tn2059); oops-1(tn1898)*	16<br>8	25<br>11	1	1.134
12	*spe-11(tn2059)*<br>*spe-11(tn2059); oops-1(tn1898)*	9<br>8	10<br>11	1	0.813

Contingency tables for Fisher's exact test for the data in Table 2 calculated using the R package [R Core Team. (2025). R: A language and environment for statistical computing. R Foundation for Statistical Computing].

Table S4. Mutations identified by whole-genome sequencing (WGS) (related to Table 5).

Available for download at

https://journals.biologists.com/dev/article-lookup/doi/10.1242/dev.204674#supplementary-data

Table S5. *C. elegans* strains used in this study

Strain	Genotype	Comments
N2	Wild type, Bristol isolate	
AD200	*unc-119(ed3)* III*; asIs1[pie-1p::gfp::egg-3 + unc-119(+)]*	
AD226	*egg-3(tm1191)/mIn1[mIs14 dpy-10(e128)]* II	
AG212	*unc-119(ed3)* III*; avIs143[cbd-1p::cbd-1::mCherry::cbd-1 3'utr + unc-119(+)]*	
AJL30	*oops-1(tn1898) ltIs37[pie-1p::mCherry::his-58 + unc-119(+)]/tmC25* IV*; ruIs57[pie-1::gfp::tba-2 + unc-119(+)]*	
AJL32	*spe-11(hc90)/tmC18* I*; ltIs37[pie-1p::mCherry::his-58 + unc-119(+)]* IV*; ruIs57[pie-1::gfp::tba-2 + unc-119(+)]*	
AJL56	*spe-11(tn2059)/tmC18(dpy-5[tmIs1236]) I; him-8 (el489)* IV	
AJL66	*chs-1(ude35[egfp::chs-1])* I	
AJL72	*spe-11(tn2059)/tmC18(dpy-5[tmIs1236])* I*; avIs143[cbd-1p::cbd-1::mCherry + unc-119(+)]*	
AJL79	*chs-1(ude35[egfp::chs-1])* I*; oops-1(tn1898)/tmC25(unc-5[tnIs1241]) IV; him-5(e1490)* V	
AJL120	*unc-119(ed3)* III; *spe-11(tn2059)/tmC18(dpy-5[tmIs1236])* I*; oops-1(tn1898) ltIs37[unc-119(+) pie-1p::mCherry::H2B])/tmC25[unc-5(tmIs1241)] ltIs37[unc-119(+) pie-1p::mCherry::H2B])* IV*; ruIs57[pie-1p::β-tubulin::GFP; unc-119(+)]*	
BA717	*spe-11(hc90); sDp2(I,f)*	Reduction-of-function mutant allele of *spe-11* (D145E and W191Stop)
BS553 [a]	*fog-2(oz40)* V	
CB3855	*plg-1(e2001)* III*; him-5(e1490)* V	
CGC43	*unc-4(e120)/mnC1[dpy-10(e128) unc-52(e444) umnIs32]* II	Balancer chromosome marked with *myo-2::GFP*
DG4746	*oops-1(tm6141)/tmC25[unc-5(tmIs1241)]* IV	Deletion allele of *oops-1*, removes 622 bp (see Fig. S1)
DG4753	*oops-1(tm6141)/tmC25[unc-5(tmIs1241)]* IV*; him-5(e1490)* V	
DG4763 [a]	*oops-1(tm6141)/tmC25[unc-5(tmIs1241)]* IV*; fog-2(oz40)* V	
DG4778	*oops-1(tn1898)/tmC25[unc-5(tmIs1241)]* IV	Entire open reading frame deletion allele of *oops-1*
DG4800	*oops-1(tn1908[gfp::tev::3xflag::oops-1a])* IV	N-terminal GFP fusion of *oops-1a/c*
DG4806	*oops-1(tn1914[oops-1::gfp::tev::3xflag])* IV	C-terminal GFP fusion of *oops-1*
DG4826	*lin-41(n2914)* I/*hT2[qIs48]* (I; III)*; oops-1(tn1908[gfp::tev::3xflag::oops-1a])* IV	
DG4833	*oops-1(tn1898)/tmC25[unc-5(tmIs1241)]* IV*; asIs3[egg-1::gfp + unc-119(+)]*	
DG4835	*oops-1(tn1898)/tmC25[unc-5(tmIs1241)]* IV*; asIs4[egg-2::gfp + unc-119(+)]*	
DG4837	*oops-1(tn1898)/tmC25[unc-5(tmIs1241)]* IV*; asIs1[pie-1p::gfp::egg-3 + unc-119(+)]*	
DG4839	*oops-1(tn1898)/tmC25(unc-5[tnIs1241])* IV*; axIs1140[pie-1p::gfp::mbk-2 + unc-119(+)]*	
DG4873	*pmnSi5[perm-4p::perm-4::mCherry + unc-119(+)]* II	

Development • Supplementary information

DG4875	*oops-1(tn1898)/tmC25(unc-5[tnIs1241])* IV*; avIs143 [cbd-1p::cbd-1::mCherry + unc-119(+)]*	
DG4877	*pmnSi1[perm-2p::perm-2::mCherry + unc-119(+)]* II*; oops-1(tn1898)/tmC25[unc-5(tmIs1241)]* IV	
DG4879	*pmnSi5[perm-4p::perm-4::mCherry + unc-119(+)]* II*; oops-1(tn1898)/tmC25[unc-5(tmIs1241)]* IV	
DG4883	*his-72(uge30[gfp::his-72])* III*; oops-1(tn1898)/tmC25[unc-5(tmIs1241)]* IV	
DG4890	*oops-1(tn1927[gfp::tev::3xflag::oops-1a]) oma-1(zu405te33)* IV	
DG4904	*oops-1(tn1927[gfp::tev::3xflag::oops-1a]) oma-1(zu405te33)/nT1[qIs51]* IV*; oma-2(te51)/nT1[qIs51]* V	
DG4912 [a]	*oops-1(tn1908[gfp::tev::3xflag::oops-1a])* IV*; fog-2(oz40)* V	
DG4915 [a]	*his-72(uge30[gfp::his-72])* III*; fog-2(oz40)* V	
DG4917	*lin-41(n2914)/tmC18[dpy-5(tmIs1236)]* I*; oops-1(tn1927[gfp::tev::3xflag::oops-1a]) oma-1(zu405te33)/nT1[qIs51]* IV*; oma-2(te51)/nT1[qIs51]* V	
DG4954	*oops-1(tn1898)/tmC25[unc-5(tmIs1241)]* IV*; him-5(e1490)* V	
DG4956 [a]	*his-72(uge30[gfp::his-72])* III*; oops-1(tn1898)/tmC25[unc-5(tmIs1241)]* IV*; fog-2(oz40)* V	
DG4984	*spe-11(ok2143)/tmC18[dpy-5[tmIs1236)]* I	Strong loss-of-function *spe-11* allele, 1x backcrossed
DG4985	*spe-11(hc90)/tmC18[dpy-5[tmIs1236)]* I	
DG4993	*oops-1(tn1908[gfp::tev::3xflag::oops-1a] tn1935)* IV	Deletion A (GFP::OOPS-1 Δ1–102 bp *3'UTR*) in Fig. 6A
DG4997	*oops-1(tn1908[gfp::tev::3xflag::oops-1a] tn1938)* IV	Deletion B (GFP::OOPS-1 Δ103–203 bp *3'UTR*) in Fig. 6A
DG4999	*oops-1(tn1908[gfp::tev::3xflag::oops-1a] tn1940)* IV	Deletion C (GFP::OOPS-1 Δ204–358 bp *3'UTR*) in Fig. 6A
DG5005	*oops-1(tn1908[gfp::tev::3xflag::oops-1a] tn1944)* IV	Deletion D (GFP::OOPS-1 Δ1–203 bp *3'UTR*) in Fig. 6A
DG5009	*oops-1(tn1908[gfp::tev::3xflag::oops-1a] tn1947)* IV	Deletion E (GFP::OOPS-1 Δ103–358 bp *3'UTR*) in Fig. 6A
DG5013	*oops-1(tn1908[gfp::tev::3xflag::oops-1a])* IV*; him-5(e1490)* V	
DG5017	*oops-1(tn1908[gfp::tev::3xflag::oops-1a] tn1949*ts*)* IV	Deletion F (GFP::OOPS-1 Δ1–358 bp *3'UTR*) in Fig. 6A
DG5033	*oops-1(tn1908[gfp::tev::3xflag::oops-1a] tn1954)* IV	Deletion G (GFP::OOPS-1 ΔM1–V28) in Fig. 6A
DG5037	*oops-1(tn1908[gfp::tev::3xflag::oops-1a] tn1957)* IV	Deletion H (GFP::OOPS-1 ΔK29–N65) in Fig. 6A
DG5045	*oops-1(tn1908[gfp::tev::3xflag::oops-1a] tn1961)* IV	Deletion I (GFP::OOPS-1 ΔG66–S109) in Fig. 6A
DG5047	*oops-1(tn1908[gfp::tev::3xflag::oops-1a] tn1963)* IV	Deletion J (GFP::OOPS-1 ΔG110–I138) in Fig. 6A
DG5049	*oops-1(tn1908[gfp::tev::3xflag::oops-1a] tn1965)/tmC25[unc-5(tmIs1241)]* IV	Deletion K (GFP::OOPS-1 ΔT139–M201) in Fig. 6A

Development • Supplementary information

DG5051	*oops-1(tn1908[gfp::tev::3xflag::oops-1a] tn1967)/tmC25[unc-5(tmIs1241)]* IV	Deletion L (GFP::OOPS-1 ΔI202–S209) in Fig. 6A
DG5053	*oops-1(tn1908[gfp::tev::3xflag::oops-1a] tn1969)/tmC25[unc-5(tmIs1241)]* IV	Deletion M (GFP::OOPS-1 ΔR210–F238) in Fig. 6A
DG5055	*oops-1(tn1908[gfp::tev::3xflag::oops-1a] tn1971)/tmC25[unc-5(tmIs1241)]* IV	Deletion N (GFP::OOPS-1 ΔK239–Y284) in Fig. 6A
DG5065	*oops-1(tn1908[gfp::tev::3xflag::oops-1a] tn1975)/tmC25[unc-5(tmIs1241)]* IV	Deletion O (GFP::OOPS-1 ΔV285–V328) in Fig. 6A
DG5067	*oops-1(tn1908[gfp::tev::3xflag::oops-1a] tn1977)* IV	Deletion P (GFP::OOPS-1 ΔT329–P374) in Fig. 6A
DG5097	*oops-1(tn1908[gfp::tev::3xflag::oops-1a] tn1983)* IV	Deletion Q (GFP::OOPS-1 ΔL375–P416) in Fig. 6A
DG5099	*oops-1(tn1908[gfp::tev::3xflag::oops-1a] tn1985)* IV	Deletion R (GFP::OOPS-1 ΔL417–S474) in Fig. 6A
DG5101	*oops-1(tn1908[gfp::tev::3xflag::oops-1a] tn1987)* IV	Deletion S (GFP::OOPS-1 ΔL417–D443) in Fig. 6A
DG5103	*oops-1(tn1908[gfp::tev::3xflag::oops-1a] tn1989)* IV	Deletion T (GFP::OOPS-1 ΔG444–S474) in Fig. 6A
DG5105	*oops-1(tn1908[gfp::tev::3xflag::oops-1a] tn1991)/tmC25[unc-5(tmIs1241)]* IV	Deletion V (GFP::OOPS-1 ΔT139–G156) in Fig. 6A
DG5107	*oops-1(tn1908[gfp::tev::3xflag::oops-1a] tn1993)/tmC25[unc-5(tmIs1241)]* IV	Deletion W (GFP::OOPS-1 ΔP157–M201) in Fig. 6A
DG5137	*oops-1(tn1908[gfp::tev::3xflag::oops-1a] tn1995)* IV	Deletion X (GFP::OOPS-1 ΔV475–A498) in Fig. 6A
DG5139	*oops-1(tn1908[gfp::tev::3xflag::oops-1a] tn1997)* IV	Deletion Y (GFP::OOPS-1 ΔA499–L534) in Fig. 6A
DG5142	*oops-1(tn1908[gfp::tev::3xflag::oops-1a] tn2000)* IV	Deletion U (GFP::OOPS-1 ΔM1–R103) in Fig. 6A
DG5147	*oops-1(tn1908[gfp::tev::3xflag::oops-1a] tn2003)/tmC25[unc-5(tmIs1241)]* IV	Deletion Z (GFP::OOPS-1 ΔM1–L534) in Fig. 6A
DG5157	*smg-1(r861); unc-54(r293)* I; *oops-1(tn1908 tn1949ts)* IV	
DG5172	*oops-1(tn1908[gfp::tev::3xflag::oops-1a] tn2013)* IV	Deletion GJ (GFP::OOPS-1 ΔM1–I138) in Fig. 6A
DG5177	*oops-1(tn1908[gfp::tev::3xflag::oops-1a] tn2017)/tmC25[unc-5(tmIs1241)]* IV	Deletion P1Y (GFP::OOPS-1 ΔT329–L534) in Fig. 6A
DG5187	*oops-1(tn1908[gfp::tev::3xflag::oops-1a] tn2023)* IV	Deletion QY (GFP::OOPS-1 ΔL375–L534) in Fig. 6A
DG5189	*oops-1(tn1908[gfp::tev::3xflag::oops-1a] tn2025)/tmC25[unc-5(tmIs1241)]* IV	Deletion VO (GFP::OOPS-1 ΔT139–V328) in Fig. 6A
DG5195	*oops-1(tn1908[gfp::tev::3xflag::oops-1a] tn2013 tn2027)* IV	Deletion GJ+QY (GFP::OOPS-1 ΔM1–I138 and ΔL375–L534) in Fig. 6A

DG5217	*oops-1(tn1908[gfp::tev::3xflag::oops-1a] tn2013 tn2037)/tmC25[unc-5(tmIs1241)]* IV	Deletion GJ+P1Y (GFP::OOPS-1 ΔM1–I138 and ΔT329–L534) in Fig. 6A
DG5219	*oops-1(tn1908[gfp::tev::3xflag::oops-1a] tn2039)* IV	Deletion P2Y (GFP::OOPS-1 ΔL350–L534) in Fig. 6A
DG5221	*oops-1(tn1908[gfp::tev::3xflag::oops-1a] tn2013 tn2041)* IV	Deletion GJ+P2Y (GFP::OOPS-1 ΔM1–I138 and ΔL350–L534) in Fig. 6A. Expresses GFP::OOPS-1 Mini (T139-C349). Expression shown in Fig. S7
DG5223	*oops-1(tn1908[gfp::tev::3xflag::oops-1a] tn2043)* IV	Deletion P1 (GFP::OOPS-1 ΔT329–C349) in Fig. 6A
DG5225	*oops-1(tn1908[gfp::tev::3xflag::oops-1a] tn2045)* IV	Deletion P2 (GFP::OOPS-1 ΔL350–P374) in Fig. 6A
DG5227	*oops-1(tn1908[gfp::tev::3xflag::oops-1a] tn2047)/tmC25[unc-5(tmIs1241)]* IV	Deletion OP1 (GFP::OOPS-1 ΔV285–C349) in Fig. 6A
DG5237	*oops-1(tn1908[gfp::tev::3xflag::oops-1a] tn2051)/tmC25[unc-5(tmIs1241)]* IV	Deletion VP1 (GFP::OOPS-1 ΔT139–C349) in Fig. 6A
DG5266	*spe-11(tn2059)/tmC18[dpy-5[tmIs1236)]* I	Entire open reading frame deletion allele of *spe-11*
DG5275	*spe-11(ok2213)/tmC18[dpy-5[tmIs1236)]* I	Strong loss-of-function *spe-11* allele, 1x backcrossed
DG5295	*spe-11(tn2068[mScarlet::tev::3xflag::spe-11])* I	N-terminal mScarlet fusion of *spe-11*
DG5305	*spe-11(tn2068[mScarlet::tev::3xflag::spe-11])* I*; oops-1(tn1908[gfp::tev::3xflag::oops-1a])* IV	Complementary expression strain of mScarlet::SPE-11 and GFP::OOPS-1A in Fig. 4
DG5307	*spe-11(tn2068[mScarlet::tev::3xflag::spe-11])* I*; oops-1(tn1914[oops-1::gfp::tev::3xflag])* IV	Expresses OOPS-1::GFP (tagged at the C-terminus) and mScarlet::SPE-11. Expression shown in Fig. S7
DG5321	*spe-11(tn2059)/tmC18[dpy-5(tmIs1236)]* I*; him-5(e1490)* V	
DG5327	*tnSi5(Cbr-unc-119(+) + mex-5p::mScarlet::3xflag::spe-11::spe-11 3'utr (65 bp) + 318-bp downstream)* II	Ectopic expression of mScarlet:: SPE-11 in oocytes using the *mex-5* promoter and the *spe-11 3'utr*
DG5328	*tnSi6(Cbr-unc-119(+) + mex-5p::mScarlet::3xflag::spe-11::spe-11 3'utr (65 bp) + 318-bp downstream)* II	Ectopic expression of mScarlet:: SPE-11 in oocytes using the *mex-5* promoter and the *spe-11 3'utr*
DG5358 [a]	*oops-1(tn1898)/tmC25[unc-5(tmIs1241)]* IV*; fog-2(oz40)* V	
DG5368	*spe-11(tn2059)* I*; tnSi5 (Cbr-unc-119(+) + mex-5p::mScarlet::3xflag::spe-11::spe-11 3'utr)* II	
DG5370	*tnSi5(Cbr-unc-119(+) + mex-5p::mScarlet::3xflag::spe-11::spe-11 3'utr)* II*; oops-1(tn1898)/tmC25[unc-5(tmIs1241)]* IV	

DG5372	*tnSi5(Cbr-unc-119(+) + mex-5p::mScarlet::3xflag::spe-11::spe-11 3'utr)* II; *oops-1(tn1908[gfp::tev::3xflag::oops-1a])* IV	
DG5376 [a]	*tnSi5(Cbr-unc-119(+) + mex-5p::mScarlet::3xflag::spe-11::spe-11 3'utr)* II; *fog-2(oz40)* V	
DG5382	*spe-11(tn2059)* I; *tnSi6 (Cbr-unc-119(+) + mex-5p::mScarlet::3xflag::spe-11::spe-11 3'utr)* II	
DG5390 [a]	*tnSi6(Cbr-unc-119(+) + mex-5p::mScarlet::3xflag::spe-11::spe-11 3'utr)* II; *fog-2(oz40)* V	
DG5430	*spe-11(tn2094[gfp::tev::3xflag::spe-11])* I	N-terminal GFP fusion of *spe-11*
DG5462	*spe-11(tn2094[gfp::tev::3xflag::spe-11])* I; *him-5(e1490)* V	
DG5479	*tnSi10(Cbr-unc-119p::unc-119(+) + spe-11p::gfp::3xflag::oops-1a::spe-11 3'utr + 318-bp downstream)* II	Ectopic expression of GFP::OOPS-1A in sperm using the *spe-11* promoter and *3'utr*
DG5495	*tnSi10(Cbr-unc-119p::unc-119(+) + spe-11p::gfp::3xflag::oops-1a::spe-11 3'utr + 318-bp downstream)* II; *oops-1(tn1898)/tmC25[unc-5(tmIs1241)]* IV	
DG5518	*tnSi10(Cbr-unc-119p::unc-119(+) + spe-11p::gfp::3xflag::oops-1a::spe-11 3'utr + 318-bp downstream)* II; *him-5(e1490)* V	
DG5544	*tnSi12(Cbr-unc-119p::unc-119(+) + peel-1p::gfp::3xflag::oops-1a::peel-1 3'utr + 682-bp downstream)* II	Ectopic expression of GFP::OOPS-1A in sperm using the *peel-1* promoter and *3'utr*
DG5546	*tnSi12(Cbr-unc-119p::unc-119(+) + peel-1p::gfp::3xflag::oops-1a::peel-1 3'utr + 682-bp downstream)* II; *oops-1(tn1898)/tmC25[unc-5(tmIs1241)]* IV	
DG5550	*tnSi12(Cbr-unc-119p::unc-119(+) + peel-1p::gfp::3xflag::oops-1a::peel-1 3'utr + 682-bp downstream)* II; *him-5(e1490)* V	
DG5554	*tnSi14(Cbr-unc-119p::unc-119(+) + trp-3p::gfp::3xflag::oops-1a::trp-3 3'utr + 1590-bp downstream)* II	Ectopic expression of GFP::OOPS-1A in sperm using the *trp-3* promoter and *3'utr*
DG5556	*tnSi14(Cbr-unc-119p::unc-119(+) + trp-3p::gfp::3xflag::oops-1a::trp-3 3'utr + 1590-bp downstream)* II; *oops-1(tn1898)/tmC25[unc-5(tmIs1241)]* IV	
DG5560	*tnSi14(Cbr-unc-119p::unc-119(+) + trp-3p::gfp::3xflag::oops-1a::trp-3 3'utr + 1590-bp downstream)* II; *him-5(e1490)* V	
DG5594	*spe-11(tn2068[mScarlet::tev::3xflag::spe-11])* I; *oops-1 (tn1908 tn2000)* IV	Expresses GFP::OOPS-1B and mScarlet::SPE-11. Expression shown in Fig. S7
DG5610	*spe-11(tn2059)/tmC18* I; *ltIs37[pie-1p::mCherry::his-58 + unc-119(+)]* IV; *ruIs57[pie-1::gfp::tba-2 + unc-119(+)]*	
DG5611	*tnSi16(Cbr-unc-119p::unc-119(+) + spe-11p::gfp::3xflag::oops-1b::spe-11 3'utr + 318-bp downstream)* II	Ectopic expression of GFP::OOPS-1B in sperm using the *spe-11* promoter and *3'utr*
DG5613	*tnSi16(Cbr-unc-119p::unc-119(+) + spe-11p::gfp::3xflag::oops-1b::spe-11 3'utr + 318-bp downstream)* II; *oops-1(tn1898)/tmC25[unc-5(tmIs1241)]* IV	
DG5617	*tnSi16(Cbr-unc-119p::unc-119(+) + spe-11p::gfp::3xflag::oops-1b::spe-11 3'utr + 318-bp downstream)* II; *him-5(e1490)* V	
DG5627	*spe-11(hc90)/tmC18[dpy-5(tmIs1236)]* I; *oops-1(tn1898)/tmC25[unc-*	

	5(tmIs1241)] IV; *ruIs57; ltIs37*	
DG5643	*spe-11(tn2094[gfp::tev::3xflag::spe-11] tn2139)* I	Deletion A (GFP:: SPE-11 ΔM1–N14) in Fig. 6D
DG5645	*spe-11(tn2094[gfp::tev::3xflag::spe-11] tn2141)* I	Deletion B (GFP:: SPE-11 ΔK15–S29) in Fig. 6D
DG5647	*spe-11(tn2094[gfp::tev::3xflag::spe-11] tn2143)* I	Deletion C (GFP:: SPE-11 ΔQ30–Y43) in Fig. 6D
DG5649	*spe-11(tn2094[gfp::tev::3xflag::spe-11] tn2145*ts*)* I	Deletion D (GFP:: SPE-11 ΔP44–S57) in Fig. 6D
DG5651	*spe-11(tn2094[gfp::tev::3xflag::spe-11] tn2146)* I	Deletion E (GFP:: SPE-11 ΔD58–K72) in Fig. 6D
DG5653	*spe-11(tn2094[gfp::tev::3xflag::spe-11] tn2147)* I	Deletion F (GFP:: SPE-11 ΔK73–D87) in Fig. 6D
DG5655	*spe-11(tn2094[gfp::tev::3xflag::spe-11] tn2148)/tmC18[dpy-5[tmIs1236)]* I	Deletion G (GFP:: SPE-11 ΔM88–F101) in Fig. 6D
DG5657	*spe-11(tn2094[gfp::tev::3xflag::spe-11] tn2150)/tmC18[dpy-5[tmIs1236)]* I	Deletion H (GFP:: SPE-11 ΔL102–A115) in Fig. 6D
DG5659	*spe-11(tn2094[gfp::tev::3xflag::spe-11] tn2152)/tmC18[dpy-5[tmIs1236)]* I	Deletion I (GFP:: SPE-11 ΔR116–G129) in Fig. 6D
DG5661	*spe-11(tn2094[gfp::tev::3xflag::spe-11] tn2154)/tmC18[dpy-5[tmIs1236)]* I	Deletion J (GFP:: SPE-11 ΔF130–N144) in Fig. 6D
DG5663	*spe-11(tn2094[gfp::tev::3xflag::spe-11] tn2156)/tmC18[dpy-5[tmIs1236)]* I	Deletion K (GFP:: SPE-11 ΔD145–A158) in Fig. 6D
DG5685	*spe-11(tn2094[gfp::tev::3xflag::spe-11] tn2160)/tmC18[dpy-5[tmIs1236)]* I	Deletion L (GFP:: SPE-11 ΔT159–M172) in Fig. 6D
DG5687	*spe-11(tn2094[gfp::tev::3xflag::spe-11] tn2161)/tmC18[dpy-5[tmIs1236)]* I	Deletion M (GFP:: SPE-11 ΔE173–A185) in Fig. 6D
DG5689	*spe-11(tn2094[gfp::tev::3xflag::spe-11] tn2163)/tmC18[dpy-5[tmIs1236)]* I	Deletion N (GFP:: SPE-11 ΔR186–L200) in Fig. 6D
DG5718	*spe-11(tn2059)/tmC18[dpy-5[tmIs1236)]* I; *oops-1(tn1898)/tmC25[unc-5(tmIs1241)]* IV	
DG5723	*spe-11(tn2094[gfp::tev::3xflag::spe-11] tn2169)/tmC18[dpy-5[tmIs1236)]* I	Deletion O (GFP:: SPE-11 ΔA201–D215) in Fig. 6D
DG5725	*spe-11(tn2094[gfp::tev::3xflag::spe-11] tn2171)/tmC18[dpy-5[tmIs1236)]* I	Deletion P (GFP:: SPE-11 ΔA216–E230) in Fig. 6D
DG5738	*spe-11(tn2094[gfp::tev::3xflag::spe-11] tn2175)/tmC18[dpy-5[tmIs1236)]* I	Deletion R (GFP:: SPE-11 ΔL246–K258) in Fig. 6D
DG5743	*spe-11(tn2094[gfp::tev::3xflag::spe-11] tn2178)/tmC18[dpy-5[tmIs1236)]* I	Deletion T (GFP:: SPE-11 ΔD272–R285) in Fig. 6D
DG5745	*spe-11(tn2094[gfp::tev::3xflag::spe-11] tn2180)/tmC18[dpy-5[tmIs1236)]* I	Deletion U (GFP:: SPE-11 ΔN286–K299) in Fig. 6D
DG5747	*spe-11(tn2094[gfp::tev::3xflag::spe-11] tn2181)/tmC18[dpy-5[tmIs1236)]* I	Deletion AF (GFP:: SPE-11

Development • Supplementary information

		ΔM1–D87) in Fig. 6D
DG5749	*spe-11(tn2094[gfp::tev::3xflag::spe-11] tn2183)* I	Deletion W (GFP:: SPE-11 ΔR295–K299) in Fig. 6D
DG5759	*spe-11(tn2094[gfp::tev::3xflag::spe-11] tn2184)/tmC18[dpy-5[tmIs1236)]* I	Deletion AE (GFP:: SPE-11 ΔM1–K72) in Fig. 6D
DG5762	*spe-11(tn2094[gfp::tev::3xflag::spe-11] tn2185)/tmC18[dpy-5[tmIs1236)]* I	Deletion Q (GFP:: SPE-11 ΔL231–W245) in Fig. 6D
DG5764	*spe-11(tn2094[gfp::tev::3xflag::spe-11] tn2187)/tmC18[dpy-5[tmIs1236)]* I	Deletion S (GFP:: SPE-11 ΔF259–E271) in Fig. 6D
DG5780	*chs-1(tn2195) spe-11(tn2094[gfp::tev::3xflag::spe-11] tn2145*ts*)* I	1x outcrossed using *tmC18/+* males
DG5783	*chs-1(tn2192) spe-11(tn2094[gfp::tev::3xflag::spe-11] tn2145*ts*)* I	1x outcrossed using *tmC18/+* males
DG5829	*chs-1(tn2201) spe-11(tn2094[gfp::tev::3xflag::spe-11] tn2145*ts*)* I	7x outcrossed using *tmC18/+* males
DG5830	*chs-1(tn2198) spe-11(tn2094[gfp::tev::3xflag::spe-11] tn2145*ts*)* I	7x outcrossed using *tmC18/+* males
DG5833	*chs-1(tn2210) spe-11(tn2094[gfp::tev::3xflag::spe-11] tn2145*ts*)* I	7x outcrossed using *tmC18/+* males
DG5837	*chs-1(tn2191) spe-11(tn2094[gfp::tev::3xflag::spe-11] tn2145*ts*)* I	7x outcrossed using *tmC18/+* males
DG5842	*gsp-3(tn2202) spe-11(tn2094[gfp::tev::3xflag::spe-11] tn2145*ts*)* I	8x outcrossed using *tmC18/+* males
DG5843	*spe-11(tn2094[gfp::tev::3xflag::spe-11] tn2145*ts*)* I; *egg-3(tn2205)* II	7x outcrossed using *tmC18/+* males
DG5845	*chs-1(tn2189) spe-11(tn2094[gfp::tev::3xflag::spe-11] tn2145*ts*)* I	7x outcrossed using *tmC18/+* males
DG5847	*chs-1(tn2210) spe-11(tn2094[gfp::tev::3xflag::spe-11] tn2145*ts*)* I	3x outcrossed using *tmC18/+* males
DG5855	*tnEx265[str-1p::gfp]*	
DG5866	*tnSi21(Cbr-unc-119p::unc-119(+) + oma-1p::gfp::3xflag::spe-11::oma-1 3'utr)* II	Ectopic expression of GFP:: SPE-11 in oocytes using the *oma-1* promoter and *3'utr*
DG5868	*tnSi23(Cbr-unc-119p::unc-119(+) + oma-2p::gfp::3xflag::spe-11::oma-2 3'utr)* II	Ectopic expression of GFP:: SPE-11 in oocytes using the *oma-2* promoter and *3'utr*
DG5870	*tnSi25(Cbr-unc-119p::unc-119(+) + rme-2p::gfp::3xflag::spe-11::rme-2 3'utr)* II	Ectopic expression of GFP:: SPE-11 in oocytes using the *rme-2* promoter and *3'utr*
DG5894	*tnSi27(Cbr-unc-119p::unc-119(+) + puf-5p::gfp::3xflag::spe-11::puf-5 3'utr)* II	Ectopic expression of GFP:: SPE-11 in oocytes using the *puf-5* promoter and *3'utr*
DG5913	*chs-1(ok1120)/tmC18[dpy-5[tmIs1236)]* I*; oops-1(tn1908[gfp::tev::3xflag::oops-*	

Development • Supplementary information

	1a]) IV	
DG5915	*egg-3(tm1191)/mnC1[dpy-10(e128) unc-52(e444) umnIs32]* II*; oops-1(tn1908[gfp::tev::3xflag::oops-1a])* IV	
DG5917	*egg-3(ok3651)/mnC1[dpy-10(e128) unc-52(e444) umnIs32]* II*; oops-1(tn1908[gfp::tev::3xflag::oops-1a])* IV	
DG5927	*oops-1(gk503838)* IV	1x backcrossed
DG5933	*chs-1(tn2298) spe-11(tn2094[gfp::tev::3xflag::spe-11] tn2145*ts*)* I	1x backcrossed
DG5936	*spe-11(tn2059)* I*; tnSi21(Cbr-unc-119p::unc-119(+) + oma-1p::gfp::3xflag::spe-11::oma-1 3'utr)* II	
DG5938	*spe-11(tn2059)* I*; tnSi23(Cbr-unc-119p::unc-119(+) + oma-2p::gfp::3xflag::spe-11::oma-2 3'utr)* II	
DG5942	*spe-11(tn2059)* I*; tnSi25(Cbr-unc-119p::unc-119(+) + rme-2p::gfp::3xflag::spe-11::rme-2 3'utr)* II	
DG5959	*spe-11(tn2059)/tmC18[dpy-5[tmIs1236)]* I*; tnSi5/mnC1[dpy-10(e128) unc-52(e444) umnIs32]* II	
DG5970	*spe-11(tn2059)* I*; tnSi5(Cbr-unc-119(+) + mex-5p::mScarlet::3xflag::spe-11::spe-11 3'utr)* II	
DG6013	*chs-1(tn2242) spe-11(tn2094[gfp::tev::3xflag::spe-11] tn2145*ts*)* I	7x outcrossed using *tmC18/+* males
DG6032	*spe-11(tn2094[gfp::tev::3xflag::spe-11] tn2145*ts*)* I; *egg-3(tn2190)* II	7x outcrossed using *tmC18/+* males
DG6035	*spe-11(tn2059)* I*; tnSi27(Cbr-unc-119p::unc-119(+) + puf-5p::gfp::3xflag::spe-11::puf-5 3'utr)* II	
DG6047	*oops-1(tn1898) ltIs37[pie-1p::mCherry::his-58 + unc-119(+)]/tmC25 ltIs37[pie-1p::mCherry::his-58 + unc-119(+)]* IV*; ruIs57[pie-1::gfp::tba-2 + unc-119(+)]*	
DG6052 [a]	*tnSi23(Cbr-unc-119(+) + oma-2p::gfp::3xflag::spe-11::oma-2 3'utr)* II*; fog-2(oz40)* V	
EG6699	*ttTi5605* II*; unc-119(ed3)* III*; oxEx1578[eft-3p::gfp + Cbr-unc-119(+)]*	
FAS46	*his-72(uge30[gfp::his-72])* II	
FM125	*unc-119(ed3)* III*; ruIs57[pAZ147:pie-1::β-tubulin::GFP; unc-119(+)]; ltIs37 [unc-119(+) pie-1::mCherry::H2B])* IV	
FX01031 [b]	*rog-1(tm1031)/+* II	
FX01600 [b]	*Y54F10AR.1(tm1600)/+* III	
FX02902 [b]	*Y60A9.3(tm2902)/+* X	
FX02920 [b]	*F54D5.5(tm2920)/+* II	
FX03459 [b]	*E02H1.5(tm3459)/+* II	
FX03823 [b]	*tmed-4(tm3823)/+* V	
FX04390 [b]	*R148.3(tm4390)/+* III	
FX05077 [b]	*fbxl-2(tm5077)/+* III	
FX05958 [b]	*tmem-184(tm5958)/+* X	
FX06117 [b]	*tmem-131(tm6117)/+* III	
FX06123 [b]	*C55A6.11(tm6123)/+* V	
FX06315 [b]	*H18N23.2(tm6315)/+* X	

Development • Supplementary information

FX06697 [b]	*B0416.5(tm6697)/+* X	
FX06735 [b]	*B0395.3(tm6735)/+* X	
FX07138 [b]	*garr-1(tm7138)/+* IV	
FX14551 [b]	*atx-2(tm3562)* III/*hT2[bli-4(e937) let-?(q782) qIs48]* (I; III)	
FX14556 [b]	*atx-2(tm4373)* III/*hT2[bli-4(e937) let-?(q782) qIs48]* (I; III)	
FX14680 [b]	*ept-1(tm3093)* I/*hT2[bli-4(e937) let-?(q782) qIs48]* (I; III)	
FX14731 [b]	*pygl-1(tm5211)* V/*nT1[qIs51]* (IV; V)	
FX14783 [b]	*F26E4.7(tm3127)* I/*hT2[bli-4(e937) let-?(q782) qIs48]* (I; III)	
FX14825 [b]	*E02D9.1(tm5862)* I/*hT2[bli-4(e937) let-?(q782) qIs48]* (I; III)	
FX14836 [b]	*oops-1(tm6141)* IV/*nT1[qIs51]* (IV; V)	Deletion allele of *oops-1,* removes 622 bp (see Fig. S1)
FX14861 [b]	*pigm-1(tm3400)/mIn1[mIs14 dpy-10(e128)]* II	
FX14877 [b]	*T05H10.1(tm5233)/mIn1[mIs14 dpy-10(e128)]* II	
FX14889 [b]	*R186.3(tm5639)* V/*nT1[qIs51]* (IV; V)	
FX15076 [b]	*pigg-1(tm2377)/dpy-10(e128)* II	
FX16511 [b]	*W04D2.4(tm586)* V/*nT1[qIs51]* (IV; V)	
FX16640 [b]	*C06A5.3(tm5259)* I/*hT2[bli-4(e937) let-?(q782) qIs48]* (I; III)	
FX16751 [b]	*rbmx-2(tm6741)/mIn1[mIs14 dpy-10(e128)]* II	
FX16752 [b]	*metl-13(tm6756)* IV/*nT1[qIs51]* (IV; V)	
FX16798 [b]	*algn-9(tm3297)/+* II	
FX16946 [b]	*efr-3(tm862)/mIn1[mIs14 dpy-10(e128)]* II	
FX17004 [b]	*T26C5.3(tm1317)/unc-4(e120)* II	
FX17310 [b]	*B0025.4(tm6909)* I/*hT2[bli-4(e937) let-?(q782) qIs48]* (I; III)	
FX17625 [b]	*znf-706(tm4820)* IV/*nT1[qIs51]* (IV; V)	
FX17944 [b]	*E02D9.1(tm6625)* I/*hT2[bli-4(e937) let-?(q782) qIs48]* (I; III)	
FX17951 [b]	*Y110A7A.7(tm6680)* I/*hT2[bli-4(e937) let-?(q782) qIs48]* (I; III)	
FX17983 [b]	*rfk-1(tm7119)* IV/*nT1[qIs51]* (IV; V)	
FX18183 [b]	*nfs-1(tm3516)* I/*hT2[bli-4(e937) let-?(q782) qIs48]* (I; III)	
FX18190 [b]	*mjl-1(tm1651)* I/*hT2[bli-4(e937) let-?(q782) qIs48]* (I; III).	
FX18202 [b]	*R186.3(tm5542)* V/*nT1[qIs51]* (IV; V)	
FX18296 [b]	*F19B6.1(tm2376)* IV/*nT1[qIs51]* (IV; V)	
FX18303 [b]	*C50C3.1(tm4185)* III/*hT2[bli-4(e937) let-?(q782) qIs48]* (I; III)	
FX18488 [b]	*Y57A10A.31(tm2349)/mnC1[dpy-10(e128) unc52(e444) nIs190 let-?]* II	
FX18582 [b]	*C09D4.4(tm4773)* I/*hT2[bli-4(e937) let-?(q782) qIs48]* (I; III)	
FX19059 [b]	*sec-61.B(tm1986)/tmIn3* IV	
FX19152 [b]	*F56C11.3(tm6013)* I/*hT2[bli-4(e937) let-?(q782) qIs48]* (I; III)	
FX19197 [b]	*F56C11.3(tm5845)* I/*hT2[bli-4(e937) let-?(q782) qIs48]* (I; III)	
FX19220 [b]	*B0334.5(tm4576)/mIn1[mIs14 dpy-10(e128)]* II	
FX19232 [b]	*B0334.5(tm4759)/mIn1[mIs14 dpy-10(e128)]* II	
FX19256 [b]	*C32D5.8(tm5559)/mIn1[mIs14 dpy-10(e128)]* II	
FX19279 [b]	*C18E9.2(tm2236)/mIn1[mIs14 dpy-10(e128)]* II	
FX19298 [b]	*Y53C12B.1(tm2353)/mIn1[mIs14 dpy-10(e128)]* II	
FX19347 [b]	*ZK546.5(tm769)/mIn1[mIs14 dpy-10(e128)]* II	

FX19350 [b]	*F56D1.1(tm814)/mIn1[mIs14 dpy-10(e128)]* II	
FX19441 [b]	*F39H2.3(tm2016)* I/*hT2[bli-4(e937) let-?(q782) qIs48]* (I; III)	
FX20937 [b]	*tm10937*(I)*; wrn-1(tm764)* II*; polq-1(tm9609) exo-1(tm1842)* III*; C42C1.8 C42C1.12 (tm10938)/+* IV	
FX21247 [b]	*wrn-1(tm764)* II*; polq-1(tm9609) F25B5.6 F25B5.3 F25B5.7 F25B5.2 F25B5.9 etc (tm11247)/+ exo-1(tm1842)* III*; tm11249/tmC5[F36H1.3(tmIs1220) tm7167]* IV	
FX30168	*tmC18[dpy-5(tmIs1236)]* I	Balancer chromosome marked with *myo-2p::mCherry*
FX30203	*tmC25[unc-5(tmIs1241)]* IV	Balancer chromosome marked with *myo-2p::Venus*
FX31218 [b]	*D2013.8 D2013.9 F42A8.1 F42A8.2 F42A8.3 C06C3.1(tm8373)/mIn1[mIs14 dpy-10(e128)]* II	
FX31257 [b]	*C55B6.4 C55B6.7 C55B6.2 C55B6.1 C55B6.5 ZK867.2 ZK867.3 ZK867.1 F46H5.3 (tm10686)/tmC30[ubc-17(tmIs1247)]* X	
FX31302 [b]	*ZK418.4 ZK418.5 ZK418.6 ZK418.7 ZK418.8 ZK418.9 ZK418.2 ZK418.1 B0280.5 B0280.6 B0280.4 (tm11024)* III	
FX31303 [b]	*K11D2.2 K11D2.3 K11D2.5 K11D2.4 K11D2.6 (tm11283)* I/*hT2[bli-4(e937) let-?(q782) qIs48]* (I; III)	
FX31304 [b]	*F35E12.8 F20G2.1 F20G2.9 F20G2.10 F20G2.2 F20G2.7 F20G2.6 F20G2.3 F20G2.4 F20G2.5 (tm11108)* V/*nT1[qIs51]* (IV; V)	
FX31307 [b]	*F32H2.9 F32H2.6 F36F2.7 F36F2.11 F36F2.4 F36F2.6 F36F2.3 F36F2.8 F36F2.2 F36F2.1 (tm11208)* I/*hT2[bli-4(e937) let-?(q782) qIs48]* (I; III).	
FX31308 [b]	*K08D10.9 K08D10.8 K08D10.7 K08D10.18 K08D10.5 K08D10.13 K08D10.4 K08D10.3 K08D10.2 K08D10.12 K08D10.1 K08D10.11 K06B9.3 K06B9.4 (tm11365)* IV/*nT1[qIs51]* (IV; V)	
FX31314 [b]	*C25A1.18 sup-46 C25A1.19 C25A1.17 C25A1.5 C25A1.16 C25A1.6 C25A1.7 C25A1.8 C25A1.9 C25A1.10 C25A1.12 C25A1.13 C25A1.11 C25A1.15 Y106G6E.1 (tm11402)* I/*hT2[bli-4(e937) let-?(q782) qIs48]* (I; III)	
FX31316 [b]	*B0350.2 B0350.74 B0350.83 C46G7.5 C46G7.111 C46G7.110 C46G7.2 C46G7.109 C46G7.1 (tm11129)* IV/*nT1[qIs51]* (IV; V)	
FX31331 [b]	*F12F6.1 F40F11.4 F40F11.3 F40F11.6 F40F11.2 F40F11.5 F40F11.1 Y24F12A.2 Y24F12A.1 tm11220/+ tm11221/tmC5 [F36H1.3(tmIs1220)]* IV	
FX31350 [b]	*tm10671/+ K10G6.3 Y14H12B.1 Y14H12B.2 (tm10672)/+ wrn-1(tm764)/+* II	
FX31351 [b]	*T23B7.3 F11G11.7 F11G11.5 F11G11.8 F11G11.4 F11G11.9 F11G11.14 F11G11.10 F11G11.11 F11G11.12 F11G11.13 F11G11.3 F11G11.2 F11G11.1 R05F9.7 (tm10689)/+ wrn-1(tm764)/+* II	
FX31352 [b]	*W08F4.16 W08F4.18 W08F4.17 W08F4.7 W08F4.8 W08F4.14 W08F4.3 W08F4.15 W08F4.9 W08F4.12 W08F4.2 W08F4.10 W08F4.11 W08F4.1 K07E8.6 K07E8.7 K07E8.5 K07E8.8 (tm11164)/+* II	
FX31354 [b]	*T02H6.1 Y39F10B.1 C08G5.1 C08G5.2 C08G5.3 C08G5.4 (tm10816)/+ wrn-1(tm764)/+* II	
FX31355 [b]	*tm10935/+ F57G8.6 W08G11.5 W08G11.1 W08G11.4 W08G11.3 W08G11.6 (tm10936)/+* V	

FX31359 [b]	wrn-1(tm764)/+ K09E4.3 algn-3 (tm10700)/+ II
FX31367 [b]	Y54G2A.16(tm11276)/+ IV
FX31368 [b]	Y41D4B.24 Y41D4B.28 K08D12.5 K08D12.4 K08D12.1 K08D12.3 K08D12.2 (tm11185)/+ IV
FX31369 [b]	F42H10.5 F42H10.6 F42H10.14 F42H10.3 F42H10.7 F42H10.2 F42H10.16 F42H10.15 C04D8.1 F42H10.11 (tm8850)/+ III
FX31375 [b]	B0212.1 Y37E11B.2 Y37E11B.1 Y37E11B.3 Y37E11B.4 Y37E11B.t1 Y37E11B.t2 (tm10723)/+ IV
FX31376 [b]	Y71A12C.1 Y71A12C.4 Y71A12C.3 Y71A12C.2 F47G4.7 F47G4.8 F47G4.1 F47G4.9 F47G4.6 F47G4.5 F47G4.4 (tm11071)/+ I
FX31382 [b]	W08G11.1 W08G11.4 W08G11.3 W08G11.6 (tm11313)/+ V
JH1576	unc-119(ed3) III; axIs1140 [pie1p::GFP::mbk-2 + unc-119(+)]
POM1	pmnSi1[perm-2p::perm-2::mCherry + unc-119(+)] II; unc-119(ed3) III
RB1189	chs-1(ok1120) I/hT2[bli-4(e937) let-?(q782) qIs48] (I; III)
RT495	unc-119(ed3) III; asIs4[egg-2::gfp + unc-119(+)]
RT497	unc-119(ed3) III; asIs3[egg-1::gfp + unc-119(+)]
VC1128 [b]	mis-12 Y47G6A.25(ok1536)/szT1[lon-2(e678)] I; +/szT1 X
VC1135 [b]	R166.3(gk541)/mIn1[mIs14 dpy-10(e128)] II
VC1259 [b]	K05C4.2 K05C4.11(ok1713)/hIn1[unc-101(sy241)] I
VC1825 [b]	F44E2.8 F44E2.9(ok2134) III/hT2[bli-4(e937) let-?(q782) qIs48] (I; III)
VC2135 [b]	lelo-2(ok2740)/sC1[dpy-1(s2170)] III
VC2238 [b]	nfs-1(ok2890) I/hT2 [bli-4(e937) let-?(q782) qIs48] (I; III)
VC2735 [b]	+/mT1 II; M142.5(ok3554)/mT1[dpy-10(e128)] III
VC2840 [b]	C24D10.4(ok3613) IV/nT1[qIs51] (IV; V)
VC2876	egg-3(ok3651)/mIn1[mIs14 dpy-10(e128)] II
VC2972 [b]	R148.3(ok3525)/qC1[dpy-19(e1259) glp-1(q339)] III
VC40188	oops-1(gk503838) IV

[a] Male-female strain.

[b] Deletion mutant strains annotated as sterile or lethal, which affect uncharacterized genes whose mRNA transcripts are associated with OMA-1 and/or LIN-41 (Tsukamoto et al., 2017). In strains containing multiple deletions, the genes that were identified as encoding OMA-1 and/or LIN-41-associated transcripts are underlined.

Table S6. Sequence of oligonucleotides used in this study for generation of plasmids, genome editing, PCR, and sequencing.

Available for download at
https://journals.biologists.com/dev/article-lookup/doi/10.1242/dev.204674#supplementary-data

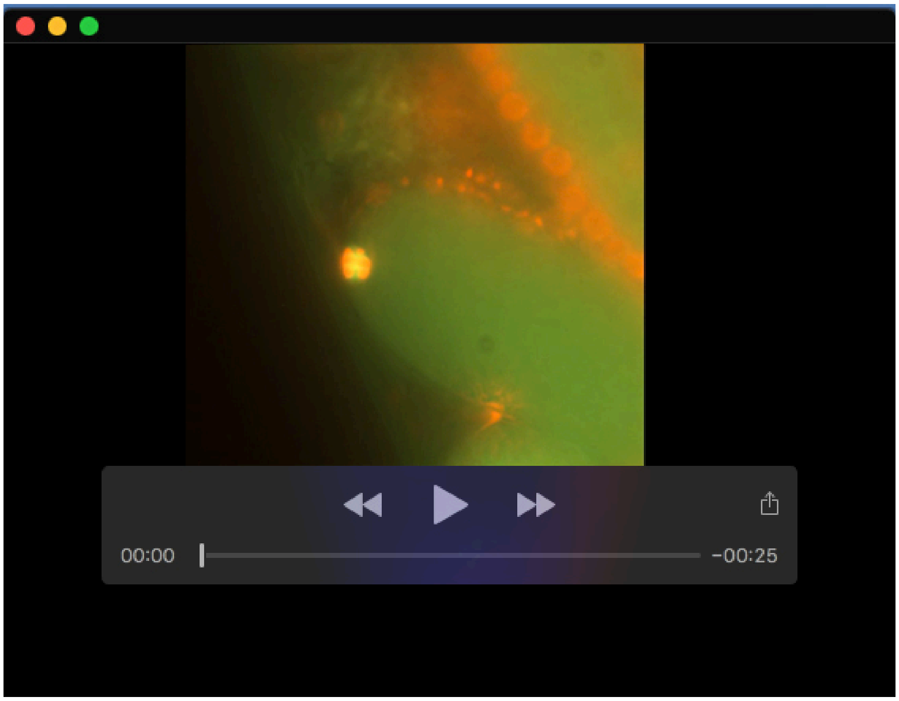

Movie 1. Time-lapse imaging of oocyte meiosis in the wild type. Chromatin is labelled in red, and microtubules are labelled in green. Time-lapse taken at 30 sec intervals for 49.5 min (100 frames).

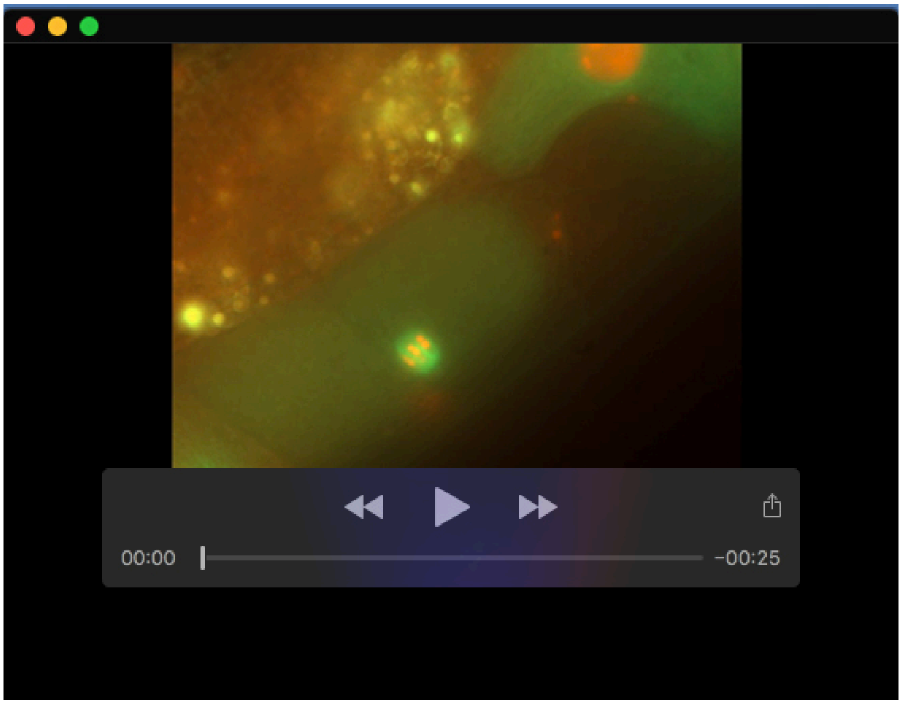

Movie 2. Time-lapse imaging of oocyte meiosis in an *oops-l(tnl898)* null mutant. Segregation of chromosomes at meiosis I occurs but the first polar body fails to form, and the chromosomes collapse back into the oocyte. The meiosis II segregation event occurs with double the number of chromosomes, and the second polar body fails to form. This mutant phenotype is referred to as the "completion phenotype" in the main text. Chromatin is labelled in red, and microtubules are labelled in green. Time-lapse taken at 30 sec intervals for 41.5 min (84 frames).

Development • Supplementary information

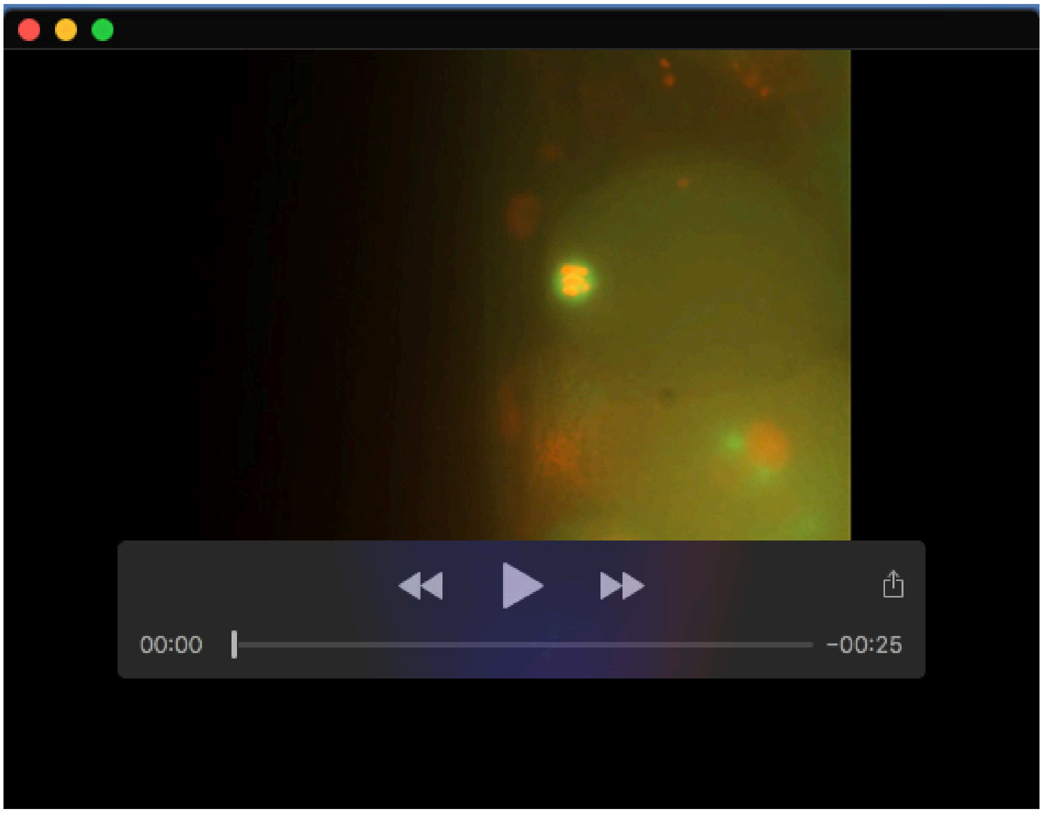

Movie 3. Time-lapse imaging of oocyte meiosis in an *oops-l(tnl898)* null mutant showing an example of the meiotic arrest phenotype. Segregation of chromosomes at meiosis I occurs but the first polar body fails to form, and the chromosomes collapse back into the oocyte. Chromosome segregation during meiosis II does not occur and the meiosis II spindle that encircles the chromosomes appears to fall apart. Chromatin is labelled in red, and microtubules are labelled in green. Time-lapse taken at 30 sec intervals for 64.5 min (130 frames).

Development • Supplementary information

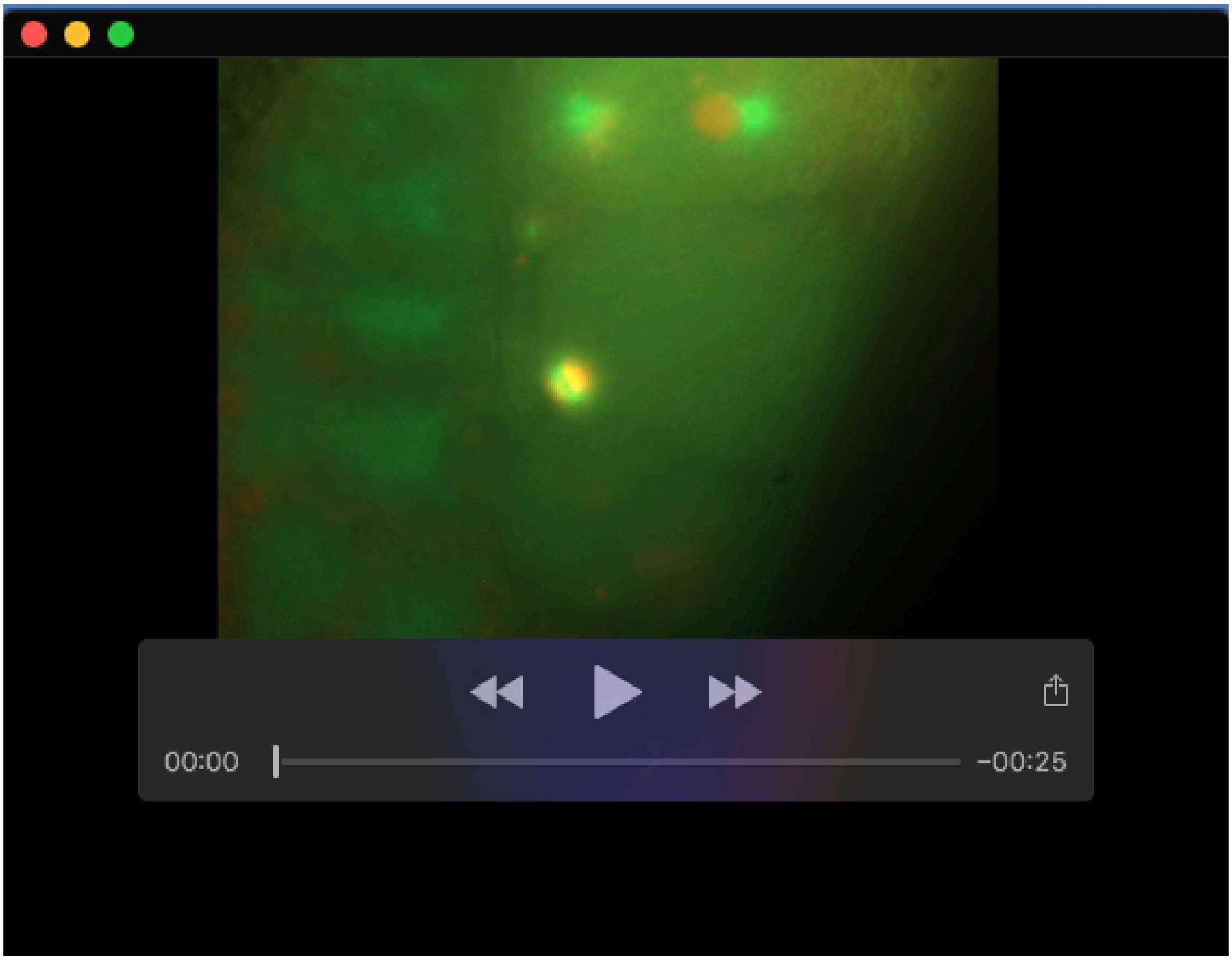

Movie 4. Time-lapse imaging of oocyte meiosis in an *oops-1(tn1898)* null mutant showing an example of polyspermy. Note two centrosomes duplicate to form a tetrapolar spindle with three pronuclei, which are then joined by a fourth nucleus of meiotic products. Chromatin is labelled in red, and microtubules are labelled in green. Time-lapse taken at 30 sec intervals for 26 min (53 frames).

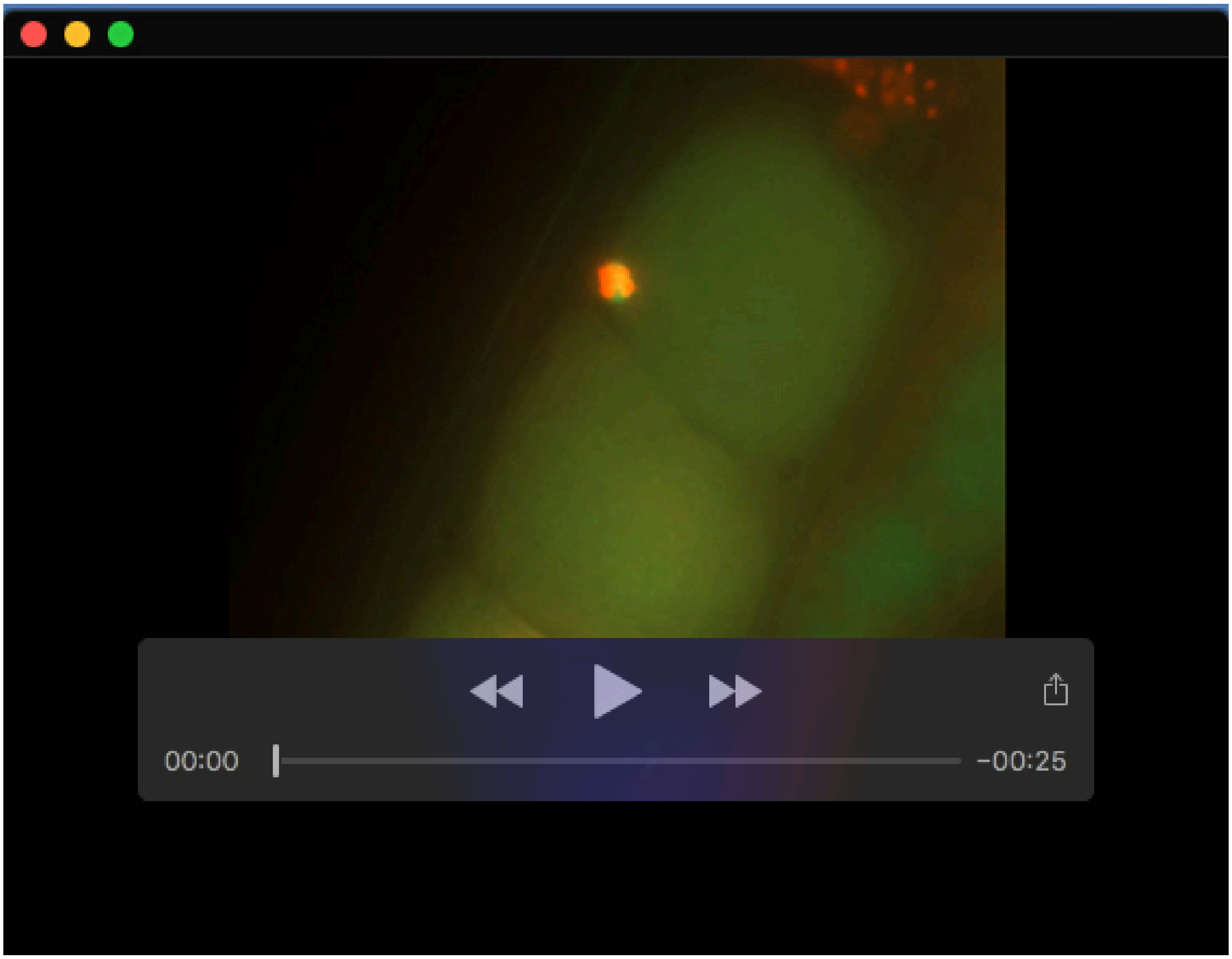

Movie 5. Time-lapse imaging of oocyte meiosis in a *spe-11(hc90)* mutant showing an example of the completion phenotype. Chromatin is labelled in red, and microtubules are labelled in green. Time-lapse taken at 30 sec intervals for 54.5 min (110 frames).

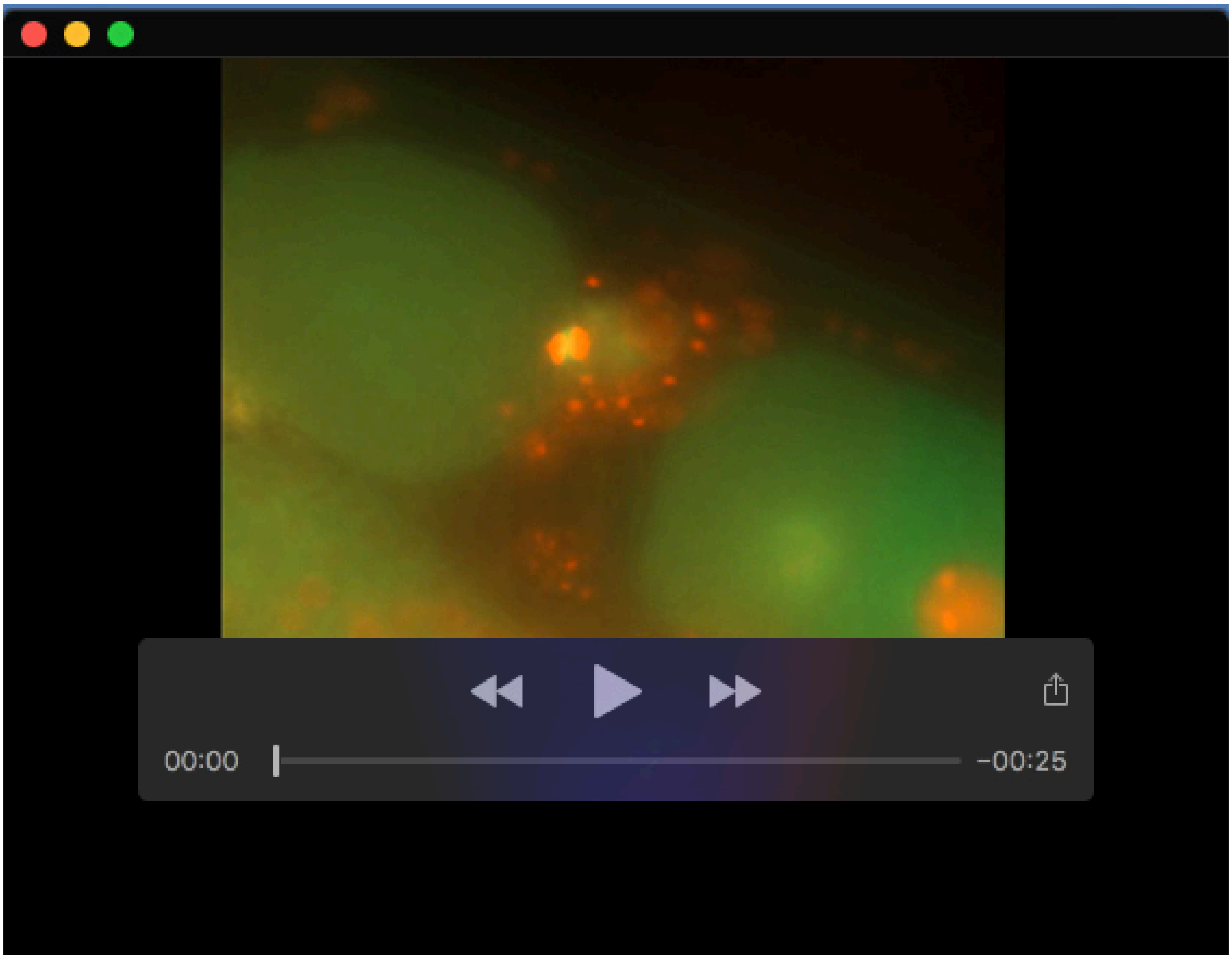

Movie 6. Time-lapse imaging of oocyte meiosis in a *spe-11(tn2059)* null mutant showing an example of the completion phenotype. Chromatin is labelled in red, and microtubules are labelled in green. Time-lapse taken at 30 sec intervals for 39.5 min (80 frames).

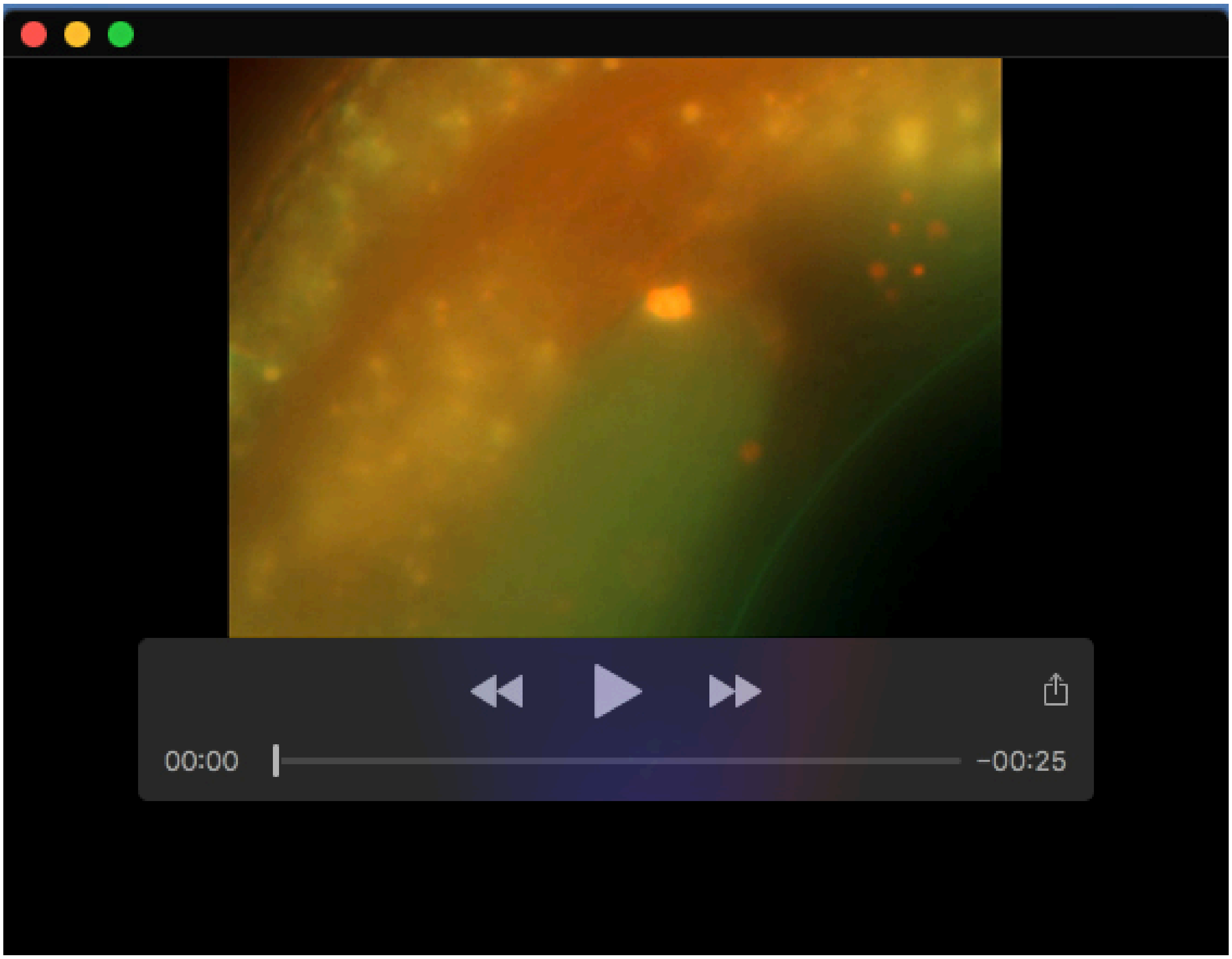

Movie 7. Time-lapse imaging of oocyte meiosis in a *spe-11(tn2059)* null mutant showing an example of the meiotic arrest phenotype. Homologous chromosome segregation during meiosis I fails and the spindle fibers that encircle the chromosomes appear to fall apart. Chromatin is labelled in red, and microtubules are labelled in green. Time-lapse taken at 30 sec intervals for 47 min (95 frames).
