# Supplementary material for: A sperm–oocyte protein partnership required for egg activation in Caenorhabditis elegans
Source: Development. 2025 Jun 25;152(12):dev204674. doi: 10.1242/dev.204674 (PMC12273631; doi:10.1242/dev.204674)
Supplement: Supplementary file 1 [file develop-152-204674_review_history.pdf]

A sperm-oocyte protein partnership required for egg activation in Caenorhabditis elegans

Tatsuya Tsukamoto, Ji Kent Kwah, Mark E. Zweifel, Naomi Courtemanche, Micah D. Gearhart, Katherine M. Walstrom, Aimee Jaramillo-Lambert and David Greenstein
DOI: 10.1242/dev.204674

Editor: Swathi Arur

Review timeline

Original submission:	24 January 2025
Editorial decision:	25 March 2025
First revision received:	1 May 2025
Editorial decision:	14 May 2025
Second revision received:	14 May 2025
Accepted:	15 May 2025

Original submission

First decision letter

MS ID#: dev.204674

MS TITLE: A sperm-oocyte protein partnership required for egg activation in Caenorhabditis elegans

AUTHORS: Tatsuya Tsukamoto; Ji Kent Kwah; Mark E. Zweifel; Naomi Courtemanche; Micah D. Gearhart; Katherine M. Walstrom; Aimee Jaramillo-Lambert; David Greenstein

Dear David,

I have now received all the referees' reports on the above manuscript, and have reached a decision. The referees' comments are appended below, or you can access them online: please go to:

The referees are both generally enthusiastic about the work, but they have slightly different views on the clarity of the manuscript. Both reviewers agree on the depth and breadth of the study. However, Reviewer 2 has raised concerns about the dense nature of the writing and suggests revisions that are primarily textual, though some may involve reanalysis of the data and changes to the presentation. If you are able to revise the manuscript based on these suggestions, I would be happy to receive the revised version. The revised paper will be re-reviewed by one or more of the original referees.

Reviewer 1

SUMMARY OF THE ADVANCE MADE IN THIS PAPER AND ITS POTENTIAL SIGNIFICANCE TO THE FIELD
Tsukamoto et al. A sperm-oocyte protein partnership required for egg activation in Caenorhabditis elegans

Summary and recommendation
The union of sperm and egg is a dividing line between oogenesis and embryonic development. Early embryonic development is heavily influenced by maternal factors, but paternal factors are much less well understood. The identification of paternal effect mechanisms is rarely defined by mutation. This paper moves the field forward in a significant way by identifying new a new factor

© 2025. Published by The Company of Biologists under the terms of the Creative Commons Attribution License (https://creativecommons.org/licenses/by/4.0/).

(OOPS-1) that interact with a paternally provided gene product (SPE-11) to regulate the completion of meiosis and the oocyte-to-embryo transition. This paper is a rigorous, methodical, and complete report of this work. Arguably, this very data rich paper could have been split into at least two publishable units. I fully support the publication of this paper in Development in its current form. After my summary of the experimental approaches below, I only have a few minor suggestions that can be at the authors discretion to address.

Experimental approach
The authors identified a deletion allele of a gene (oops-1) regulated by the RNA binding proteins OMA-1 and LIN-41. Loss of function results in inviable progeny due to an oocyte specific defect. They also exhibited defects in the block to polyspermy and eggshell formation. The authors observed the completion of meiosis and observed defects. Using biochemical methods (OOPS-1 TAP and mass spec) the authors were able to identify SPE-11 as an interactor. The reverse experiment with SPE-11 TAP MS recovered OOPS-1. GSP-3/4 was also found to be a consistent interactor. Although the requirement for spe-11 and oops-1 on in different gametes, they both result in an embryonic lethal phenotype. Examination of different alleles of spe-11 including a new null allele of the gene showed essentially identical mutant phenotypes with oops-1 and double mutants do not exhibit any new phenotypes. An GFP::OOPS-1 is expressed in the adult oogenic germline and different isoform expression was analyzed. It was concluded that SPE-11 and OOPS-1 unite at fertilization. The authors reconfirmed previous work that expression of SPE-11 in oocytes is sufficient to rescue embryonic lethality. Expression of mScarlet::SPE-11 is dependent on OOPS-1 expression and likely indicates that the two proteins co-localize in vivo. It does not mean that they directly interact in vivo (line 252). Unlike SPE-11 function when supplied to either gamete. Ectopic expression of OOPS-1 in sperm does not rescue the loss of function in oocytes. A structure function analysis dissected regions of the protein sufficient of OOPS-1 function as well as regions in both proteins that could be important for their protein-protein interactions with each other. The authors also conducted a screen for suppressors of a temperature sensitive allele of spe-11. Numerous loci were identified including gene encoding components of the EGG complex. Taking advantage of their mass spec data, the authors found evidence of OOPS-1 and SPE-11 phosphorylation. They also found that the OOPS-1-SPE-11 complex can bind to F-actin and decrease F-actin decreases the concentration of F-actin in vitro. Further in vitro experiments suggested that the OOPS-1-SPE-11 complex inhibits actin filament nucleation.

SUGGESTIONS TO AUTHORS
Major revisions
None required

Minor revisions suggestions at the discretion of the authors
Introduction line 108. Inscrutable means impossible to understand or interpret. Perhaps a better word is unknown. "….molecular interactions and activities have been unknown".

Was polyspermy ever observed in oops-1 mutants without mat-1 RNAi?

Figure 1C not mentioned in results section?

Reviewer 2

The manuscript describes the characterization of OOPS-1, an oocyte protein that is found to work in conjunction with a paternal-effect protein SPE-11. The authors show that SPE-11 and OOPS-1 form a complex and are required for early embryo viability, particularly in terms of oocyte meiotic progression and cytokinesis. They identify functional domains in OOPS-1 and also show that OOPS-1 and SPE-11 biochemically interact with the protein phosphatase 1 homology GSP-3/4, as well as genetically influenced by embryo cortical proteins CHS-1 and EGG complex members. Overall, there is an impressively broad set of experiments that show both genetic, biochemical, and functional interactions that expand our understanding of how paternal and maternal factors partner to co-regulate events in the early embryo. As such, it represents an important advance in our understanding of how paternal factors can work with maternal factors to induce meiotic progression in early embryos that may be applicable more broadly and represents a unique molecular example of paternal/maternal partnership.

© 2025. Published by The Company of Biologists under the terms of the Creative Commons Attribution License (https://creativecommons.org/licenses/by/4.0/).

The paper as written requires major revision mostly in terms of writing and presentation. The depth and scope of the experiments is strong and the authors do a mostly good job of drawing reasonable conclusions based on the limitations of experimental techniques. The major issue is a lack in clarity of the presentation of the figures and how they are specifically described and called out in the text. The authors should be mindful that non-expert and non-C. elegans readers will appreciate their findings with more explanations and careful labeling of figures that are less jargon heavy (less strain names).

One main question: the authors have developed fluorescently tagged OOPS-1 and SPE-11 proteins but do not show or say anything about their localization immediately after fertilization. It would be most meaningful to be able to see, if possible, the dynamics of SPE-11 and OOPS-1 protein localization in newly fertilized embryos. If the authors tried this, any description of what happens would be very informative to the conclusions they draw later, especially about polar body exclusion, etc. Were there technical issues that prevented them from analyzing this? Are protein levels too low to detect this? Descriptions about this would be good to include and may be informative about their dynamics if technical issues are not the main issue (and even if they were, a description in the methods about it would also be helpful). In general, the colocalization experiments (in the germline) were also not very clear and the authors had a strong conclusion that they supported 'direct interaction in vivo." It would strengthen the work to show localization of OOPS-1 and SPE-11 more clearly with zoomed in images and more clear labeling of cellular and intracellular features.

Below is a list of specific comments below to address.

Specific comments:

The Abstract should be rewritten:
-The authors state: "…oops-1 and spe-11 exhibit identical null phenotypes, displaying defects in meiotic progression and cytokinesis."
However, they will show that they do not exhibit identical null phenotypes. Please reword.
-"Thus, the OOPS-1-SPE-11 complex may function to promote F-actin-mediated meiotic cytokinesis." This conclusion is stated too strongly given the supporting data.
-"…GSP-3/4, which appear to promote OOPS-1-SPE-11 function." This is statement does not accurately reflect the data.
-the main highlight of the paper is finding an maternal factor that directly binds a paternal factor to facilitate meiotic progression. That is not well highlighted in the abstract as written.

Page 7 Line 128-129 and Figure 1: Please reorder the figures in order of how they are referred to in the text. Figure 1C is the first figure to be discussed. I find it difficult when figures are consistently presented in a fashion that do not reflect the logical progression in the text.

Page 7 Line 131 and Figure S1. Figure S1C shows the wildtype only on day 1 while the oops mutant is shown for days 1 and 2. Please show wildtype on day 2 so there can be a direct comparison with the mutant. Also, please refer to Figure S1A, B, and C separately in the text as they are discussed.

Page 7 Line 131: The authors state "Since oops-1 null mutants produce apparently normal numbers of sperm, we reasoned that sperm depletion might be caused by polyspermy."

The logic of this paragraph is confusing. There is a logical leap about stacking of oocytes, oops-1 mutants having normal numbers of sperm, and suspecting polyspermy. Did the authors count the number of sperm? How did they know they had normal numbers? Also, why did they reason that sperm depletion might be caused by polyspermy? They should explain why, with citations, if necessary and define polyspermy. There could be other reasons for sperm loss, not just polyspermy.

Page 8 Lines 144-149. The authors list several proteins of which they show the localization in Figures 1 and Figure S2. It is unclear why - they do not explain why they are showing all of them and they do not explain what they can conclude from the normal localization. What is a PERM complex? Why would you show MBK-2? Or EGG complex?

© 2025. Published by The Company of Biologists under the terms of the Creative Commons Attribution License (https://creativecommons.org/licenses/by/4.0/).

Pages 8-9 Lines 152-165. It is confusing to look at Figure 2 the way it is ordered with spe-11 data included when it is not referred to in the text at this point.

Page 8 Lines 157-159: The authors state that approximately half of the mutants exhibit polar body extrusion and the other half show meiotic arrest. But Table 2 is confusing - Polar body extrusion is 19/41 (which is about half) but 13+9+3=25, which if totaled with 19 is 44, not 41. Likewise, spe-11(tn2059) has 6+2+2=10 embryos that are arrested and 9 that make it through meiotic exit - so how is it that there are 11 that show polar body extrusion failure? Are some of the arrested ones extruding polar bodies?

Page 9 Lines 172-176. There is a sentence "In contrast to oops-1, spe-11…." That has nothing to do with the rest of the paragraph. It is not clear what point the authors are making by including this information here.

Page 10 Lines 198-200: The authors state that "spe-11(tn2059); oops-1(tn1898) double null mutant behaves similarly to the individual single mutants" - but the individual mutants have different phenotypes. Can this be explained better? The double doesn't undergo meiosis II arrest (like the oops-1 mutant) and seems to have different percentages of classes. Also, Table 2 has spe-11(tn2059); oops-1(tn1898) but Figure 2 does not show this double mutant. Figure 2B is not specifically referred to in the text and does not have the double mutant included. This is not required necessarily, but if they authors have this, they could include it to better show similarities.

Figures 3 and File S3. I find that TAP purification to be more compelling to show association of OOPS-1 and SPE-11. I would recommend including Figure S3C in Figure 3.

Page 10 Lines 203-204, Methods. The authors state "We generated viable and fertile N- and C-terminal fusions of OOPS-1 to GFP." It was not clear from the text, Figure S1, or the Methods if these were generated by CRISPR at the endogenous locus or through MosSCI (why are some overexpressed?). It should be clarified in the text, Figure S1, and the legends. In the methods, it would be helpful if the authors stated more explicitly how each strain was made. The title of "Genome Editing" is not very clear to understand exactly how each construct was used to create which strains that were used for each experiment shown in the figures. Including strain names in the figure is accurate but makes it hard to follow what constructs are being analyzed, especially as the strain names are so similar. Descriptor names could be useful to help the reader follow better.

Figure 4. What is E and F showing? Again, having specific call outs for each panel in the text would be helpful to the reader.

Page 11 Line 225: The authors state "Thus, the OOPS-1-SPE-11 complex likely forms and functions upon fertilization." Did the authors look in newly fertilized embryos for the dynamics of SPE-11 and OOPS-1? If not, why? If so, what did they see?

Page 12 Lines 245-248: Is this data not shown? It is not shown in Table 4. Are the levels of rescue the same? It is not required to show the data, but it is confusing to mention it and not show the data or say it is data not shown. Any differences in levels of rescue should also be noted if there are any. It also may be confusing to non-experts why these promoters were chosen.

Page 12 Lines 251-252. The authors state "mScarlet::SPE-11 exhibits nuclear localization (Fig. 5, D and E)." It is difficult to see nuclear localization in these panels. Is it suppose to be nuclear in the germline nuclei and oocytes? This is not evident in the data shown. Also, the conclusion that "OOPS-1 and SPE-11 directly interact in vivo" is not possible to draw from this data. Co-localization is not evidence of direct interaction in vivo. It suggest they may, if they were truly shown to colocalize. Panels B and C show co-localization in germline nuclei but not early oocytes.

Page 13 Lines 258. The call out for Fig. 5 F and G - the authors do not describe what localization they observe for the GFP::OOPS-1A expressed in sperm. It is also hard to tell from the image. It the image depicting that the male sperm was transferred? What is the staining pattern of the GFP::OOPS-1A in sperm? It is hard to tell from the image presented and not discussed in the text.

Page 13 Line 264 - I could not find where IDP was originally defined.

© 2025. Published by The Company of Biologists under the terms of the Creative Commons Attribution License (https://creativecommons.org/licenses/by/4.0/).

Page 14 Line 277 - the authors state "suggesting these regions might mediate their interactions." Having high pLDDT values do not support that these regions mediate interactions. It just suggests that they have higher confidence in predication scores thus more structured.

Figure 9 and Discussion:
A summary figure that has more insight into how the complex would function in the roles discussed in the paper would be helpful.
The figure as is looks like it indicates that OOPS-1 and SPE-11 associate with CHS-1 directly to regulate eggshell formation. However, the only direct interactor found was GSP-3/4, which are not included in the figure. Why is CHS-1 shown as the direct interactor? Another interaction not included is actin and the idea of the OOPS-1-SPE-11 having a role in actin regulation that would somehow promote cytokinesis, which the authors stated was a function in the abstract twice. The authors promote that the complex function in cytokinesis - what is the model for how OOPS-1-SPE-11 promote this locally to function in cytokinesis and polar body extrusion?

Are there any other examples in other organisms of such a paternal/maternal partnership? It would strengthen and broaden the work to discuss this - point out the novelty or commonalities that might be known and in what contexts.

First revision

Author response to reviewers' comments

Response to Reviewers
We thank the reviewers and the editor for the constructive suggestions and have addressed all points raised. We have included a pdf file of the manuscript in which changes in response to the reviewers' comments are highlighted.

Reviewer 1
1. Introduction line 108. Inscrutable means impossible to understand or interpret. Perhaps a better word is unknown. "molecular interactions and activities have been unknown".
Thanks! We have made the change to "unknown" (page 5, line 92).

2. Was polyspermy ever observed in oops-1 mutants without mat-1 RNAi?
Thanks for bringing this up. Yes. Polyspermy was observed in *oops-1* null mutants by live-cell imaging. We have added a movie to the Results section (now Movie 4). Polyspermy has been observed in *spe-11* mutants in the absence of *mat-1(RNAi)* (Johnston et al., 2010; Sato and Sato 2011). We have added the Sato and Sato (2011) reference for completeness.

3. Figure 1C not mentioned in results section?
We have reorganized Figure 1 to cite the panels in order, as suggested by Reviewer 2 below.

Reviewer 2
1. The authors should be mindful that non-expert and non-C. elegans readers will appreciate their findings with more explanations and careful labeling of figures that are less jargon heavy (less strain names).
Thanks! This was an important suggestion, and we have removed the strain names from the figure legends. We have also done our best to reduce the usage of jargon.

2. One main question: the authors have developed fluorescently tagged OOPS-1 and SPE-11 proteins but do not show or say anything about their localization immediately after fertilization. It would be most meaningful to be able to see, if possible, the dynamics of SPE-11 and OOPS-1 protein localization in newly fertilized embryos. If the authors tried this, any description of what happens would be very informative to the conclusions they draw later, especially about polar body exclusion, etc. Were there technical issues that prevented them from analyzing this? Are protein

© 2025. Published by The Company of Biologists under the terms of the Creative Commons Attribution License (https://creativecommons.org/licenses/by/4.0/).

levels too low to detect this? Descriptions about this would be good to include and may be informative about their dynamics if technical issues are not the main issue (and even if they were, a description in the methods about it would also be helpful).
Browning and Strome (1996) used anti-SPE-11 antibodies and immunofluorescence to examine SPE-11 after fertilization and concluded it was below the level of detection. In general, immunofluorescence is more sensitive than fluorescent protein detection because of signal amplification and quantum yield effects. Given that the SPE-11 protein faces a 1/10,000 dilution after fertilization, their observations make sense. Our own observations of fertilized embryos from strains carrying fluorescent protein tags (e.g., GFP and mScarlet) on endogenous SPE-11 using the microscopic imaging techniques reported in Materials and Methods (wide-field microscopy and confocal microscopy) are consistent with the immunofluorescence results of Browning and Strome (1996). Thus, we will focus future efforts on developing single-molecule methods for imaging SPE-11 and OOPS-1 after fertilization using HaloTag and/or SNAP-Tag constructs. We have addressed this point in the Results section (pages 11-12, lines 228-237), as follows:

"We were unable to visualize the predicted formation of the complex after fertilization, consistent with the results of Browning and Strome (1996), who used immunofluorescence to examine SPE-11 localization after fertilization and concluded it was below the level of detection. Immunofluorescence is more sensitive than fluorescent protein detection because of signal amplification and quantum yield effects. Nevertheless, genetic analysis demonstrates that OOPS-1 and SPE-11 are required and expressed in female and male gametes, respectively. The biochemical analysis shows the two proteins form a complex that can be isolated from *C. elegans* and after co-expression in *E. coli*. Given that the *oops-1* and *spe-11* null mutant phenotypes are indistinguishable in their impact on early post-fertilization development, the inescapable inference is that the proteins must interact soon after fertilization."

In keeping with the new editorial policies of *Development*, we have added a "Limitations of this study" section to the Discussion (see page 23, lines 481-495), as follows:

"Limitations

The sexual reproduction of all animals depends on fertilization. Here we define a molecular interaction between two intracellular proteins, the oocyte protein OOPS-1 and the sperm protein SPE-11, that is required for multiple aspects of early post-fertilization development, including the completion of meiosis and eggshell formation. Yet, several questions remain about the dynamics of protein complex formation and the mechanistic aspects of complex function *in vivo*. Using standard microscopic methods (e.g., widefield and confocal microscopy) and endogenously tagged fluorescent proteins, we were unable to visualize SPE-11 post-fertilization. Examination of the dynamics of the complex after fertilization will require methods with single-molecule sensitivity to image SPE-11 from the single sperm that fertilizes the much larger oocyte. Our biochemical studies show that the OOPS-1–SPE-11 complex binds actin and inhibits actin nucleation *in vitro*. How this biochemical activity contributes at a mechanistic level to the completion of meiosis in the wild type, and its failure in *oops-1* and *spe-11* mutants, will require dynamic studies of the actin cytoskeleton *in utero* during the period in which the eggshell is being synthesized and the newly fertilized embryos are osmotically sensitive."

3. In general, the colocalization experiments (in the germline) were also not very clear and the authors had a strong conclusion that they supported 'direct interaction in vivo." It would strengthen the work to show localization of OOPS-1 and SPE-11 more clearly with zoomed in images and clearer labeling of cellular and intracellular features.
Thanks! We have revised Figure 5, accordingly. We have included insets to more clearly demonstrate localization in different regions of the gonad and to highlight co-localization. We state the conclusion of the ectopic expression experiment as, "This observation is consistent with our biochemical analyses and suggests that OOPS-1 and SPE-11 can interact *in vivo*." (page 12, line 249-250). The inference that the interaction must be direct comes from the bacterial expression and purification experiment. Please also note that in response to comment #17 below, we added Figure S8, which shows GFP::SPE-11 expressed under control of the *oma-1*, *oma-2*, *rme-2*, and *puf-5* promoters and their respective *3'UTR* sequences. In each case, we observe rescue of a *spe-11* null mutation and see cortical localization of GFP::SPE-11 in oocytes where OOPS-1 is known to be.

© 2025. Published by The Company of Biologists under the terms of the Creative Commons Attribution License (https://creativecommons.org/licenses/by/4.0/).

4. The Abstract should be rewritten:
Thanks! We have tightened up the Abstract.

5. Page 7 Line 128-129 and Figure 1: Please reorder the figures in order of how they are referred to in the text. Figure 1C is the first figure to be discussed. I find it difficult when figures are consistently presented in a fashion that do not reflect the logical progression in the text.
Thanks! We have reorganized Figure 1.

6. Page 7 Line 131 and Figure S1. Figure S1C shows the wildtype only on day 1 while the oops mutant is shown for days 1 and 2. Please show wildtype on day 2 so there can be a direct comparison with the mutant. Also, please refer to Figure S1A, B, and C separately in the text as they are discussed.
Good suggestion! We included an image of a day 2 wild-type hermaphrodite in what is now Figure S2.

7. Page 7 Line 131: The authors state "Since oops-1 null mutants produce apparently normal numbers of sperm, we reasoned that sperm depletion might be caused by polyspermy."

The logic of this paragraph is confusing. There is a logical leap about stacking of oocytes, oops-1 mutants having normal numbers of sperm, and suspecting polyspermy. Did the authors count the number of sperm? How did they know they had normal numbers? Also, why did they reason that sperm depletion might be caused by polyspermy? They should explain why, with citations, if necessary and define polyspermy. There could be other reasons for sperm loss, not just polyspermy.
Thanks! We have added the requested clarification with citations. The text accurately reflects our thought processes and the ensuing experiments we conducted, which yielded conclusive results. The text has been modified to (pages 6-7, lines 115-122):

"Oocytes in *oops-1* mutant hermaphrodites stack up in the gonad arm on the second day of adulthood (Fig. S2)—a phenotype associated with the depletion of the sperm-provided meiotic maturation signal (McCarter et al., 1999; Miller et al., 2001). Although there are several ways this phenotype can arise, hermaphrodites with mutations that affect fertilization or sperm activation exhibit nearly normal rates of meiotic maturation on the second day of adulthood and do not display the oocyte stacking phenotype (Kosinski et al., 2005). Thus, we investigated whether sperm might be consumed at a higher rate in *oops-1(tn1898)* mutants owing to polyspermy in which more than one sperm fertilizes an oocyte."

8. Page 8 Lines 144-149. The authors list several proteins of which they show the localization in Figures 1 and Figure S2. It is unclear why - they do not explain why they are showing all of them and they do not explain what they can conclude from the normal localization. What is a PERM complex? Why would you show MBK-2? Or EGG complex?
We have provided the requested clarification as follows (pages 7-8, lines 134-145):

"The chitin layer of the eggshell is synthesized by chitin synthase (CHS-1), which is a component of the EGG complex that contains proteins needed for fertilization and egg activation, including EGG-1-5 and the MBK-2 kinase (Maruyama et al., 2007; Parry et al., 2009). Similarly, the PERM complex is required for the proper formation of the outer layer of the eggshell (González et al., 2018). The chitin-binding protein, CBD-1, serves to anchor and organize the EGG and PERM complexes (Johnston et al., 2010; González et al., 2018). Therefore, we tested the possibility that the *oops-1(tn1898)* mutation interferes with eggshell formation by causing mislocalization of EGG or PERM complex proteins in oocytes or early embryos. We observed that CHS-1, and the EGG complex components EGG-1-3, and MBK-2, as well as the PERM complex components CBD-1, PERM-2, and PERM-4 (González et al., 2018), localize normally in *oops-1(tn1898)* null mutants (Figs 1D-F,S3). These results, taken together with genetic results described below, suggest that OOPS-1 promotes EGG complex activity independent of localization."

9. Pages 8-9 Lines 152-165. It is confusing to look at Figure 2 the way it is ordered with spe-11 data included when it is not referred to in the text at this point.
Good suggestion! We reorganized the figure as recommended.

© 2025. Published by The Company of Biologists under the terms of the Creative Commons Attribution License (https://creativecommons.org/licenses/by/4.0/).

10. Page 8 Lines 157-159: The authors state that approximately half of the mutants exhibit polar body extrusion and the other half show meiotic arrest. But Table 2 is confusing - Polar body extrusion is 19/41 (which is about half) but 13+9+3=25, which if totaled with 19 is 44, not 41. Likewise, spe-11(tn2059) has 6+2+2=10 embryos that are arrested and 9 that make it through meiotic exit - so how is it that there are 11 that show polar body extrusion failure? Are some of the arrested ones extruding polar bodies?
Thank you for the comment! In our attempts to be very precise, we ended up being confusing, sorry! The origin of the confusions stems from the heading, “Polar body extrusion failure” of the original Table 2. Because embryos that exhibit pre-anaphase arrest or anaphase I arrest can’t properly be said to “fail” polar body extrusion (they don’t progress to the point in the cell cycle, in which polar bodies are extruded), we said in footnote b of the originally submitted Table 2 that, “The embryos exhibiting a failure to extrude polar bodies correspond to those exhibiting the “completion” phenotype (Meiotic exit) plus those embryos that arrest in meiosis II.” We agree this is confusing. Thus, we changed the last header to “Polar body extrusion” because the point is that *oops-1* and *spe-11* null mutant embryos [and *spe-11(hc90*) for that matter] have never been observed to produce polar bodies. This should clarify the text.

11. Page 9 Lines 172-176. There is a sentence "In contrast to oops-1, spe-11…." That has nothing to do with the rest of the paragraph. It is not clear what point the authors are making by including this information here.
We have rewritten the sentence to say, “*spe-11* is one of the few known strict paternal-effect lethal mutations (L’Hernault et al., 1988)” (page 9, lines 168-169). Our point is to introduce *spe-11*.

12. Page 10 Lines 198-200: The authors state that "spe-11(tn2059); oops-1(tn1898) double null mutant behaves similarly to the individual single mutants" - but the individual mutants have different phenotypes. Can this be explained better? The double doesn't undergo meiosis II arrest (like the oops-1 mutant) and seems to have different percentages of classes. Also, Table 2 has spe-11(tn2059); oops-1(tn1898) but Figure 2 does not show this double mutant. Figure 2B is not specifically referred to in the text and does not have the double mutant included. This is not required necessarily, but if they authors have this, they could include it to better show similarities.
Thanks for bringing this up (and please see our response to point 9 above). We conducted statistical analysis of the data in Table 2 using Fisher’s exact test (see our new Table S1 also revised text on page 10, lines 192-196). The results clearly show that *oops-1(tn1898* null*)*, *spe-11(tn2059* null*)*, and the double mutant exhibit a distribution of phenotypes that are not statistically different ($p>0.1$). The contingency tables supporting this analysis are presented in Table S1. Because we did not observe meiosis II arrest in our double mutant dataset, we have added the sentence (page 10, lines 196–199),

“However, instances of meiosis I arrest, but not meiosis II arrest, were observed in *spe-11(tn2059); oops-1(tn1898)* double mutants (Table 2). Thus, we cannot exclude the possibility that OOPS-1 and SPE-11 can each exhibit residual activity in the absence of one another.” Because we did not observe instances of meiosis II arrest in the double mutant in our dataset, we haven’t included the double mutant in Figure 2B.

13. Figures 3 and File S3. I find that TAP purification to be more compelling to show association of OOPS-1 and SPE-11. I would recommend including Figure S3C in Figure 3.
We appreciate that Reviewer 2 places a higher emphasis on TAP purification than the *E. coli* expression. We have therefore added a new supplementary figure diagraming the TAP purification strategy and validating intermediate steps using western blotting (Fig. S4). We also note that Reviewer 1 correctly pointed out that the TAP purification results cannot be interpreted to mean the two proteins directly interact. However, the *E. coli* expression and purification results show that the two proteins interact in the absence of any other *C. elegans* proteins, and thus the interaction is likely to be direct. The approach taken, shown in Figure 3B involves very stringent reciprocal affinity purifications. Based on these considerations, we have not altered Figure 3.

14. Page 10 Lines 203-204, Methods. The authors state "We generated viable and fertile N- and C-terminal fusions of OOPS-1 to GFP." It was not clear from the text, Figure S1, or the Methods if these were generated by CRISPR at the endogenous locus or through MosSCI (why are some overexpressed?). It should be clarified in the text, Figure S1, and the legends. In the methods, it would be helpful if the authors stated more explicitly how each strain was made. The title of

© 2025. Published by The Company of Biologists under the terms of the Creative Commons Attribution License (https://creativecommons.org/licenses/by/4.0/).

"Genome Editing" is not very clear to understand exactly how each construct was used to create which strains that were used for each experiment shown in the figures. Including strain names in the figure is accurate but makes it hard to follow what constructs are being analyzed, especially as the strain names are so similar. Descriptor names could be useful to help the reader follow better.
All protein fusions and genomic deletions made in this study were done using CRISPR-Cas9 genome editing. The single-copy insertions used a variant of the MosSCI approach and were used only for ectopically expressing SPE-11 in the female germline and OOPS-1 in sperm (Figs 5, S8). We have clarified this in the Materials and Methods section, the text, and the figure legends. File S6 provides the details of the single-guide RNAs and the repair templates that were used for genome editing. With regards to why GFP::OOPS-1B is "over-expressed," it is likely because the deleted region of the protein is destabilizing. Please note, in our original submission and the revision, we avoided the use of "over-expressed," as in the reviewer's usage, because it connotes that the "over-expression" is due to elevated copy number or some other artificial means. Because we used genome editing approaches, the higher level of expression of mutant proteins likely indicate that the deleted regions are destabilizing at least in the fluorescence protein fusion context.

15. Figure 4. What is E and F showing? Again, having specific call outs for each panel in the text would be helpful to the reader.
Thanks! We have clarified this point (page 10, lines 207-208):

"The decline in OOPS-1 levels during late oogenesis is most apparent from the analysis of single confocal sections (Fig. 4E,F)."

16. Page 11 Line 225: The authors state "Thus, the OOPS-1-SPE-11 complex likely forms and functions upon fertilization." Did the authors look in newly fertilized embryos for the dynamics of SPE-11 and OOPS-1? If not, why? If so, what did they see?
Please see our response to point # 2 above.

17. Page 12 Lines 245-248: Is this data not shown? It is not shown in Table 4. Are the levels of rescue the same? It is not required to show the data, but it is confusing to mention it and not show the data or say it is data not shown. Any differences in levels of rescue should also be noted if there are any. It also may be confusing to non-experts why these promoters were chosen.
We have added a new supplemental figure (Fig. S8) to provide this confirmatory data. This information also further substantiates the finding that SPE-11 is cortically localized when ectopically expressed in oocytes. We added accompanying text (page 13, lines 260–278).

18. Page 12 Lines 251-252. The authors state "mScarlet::SPE-11 exhibits nuclear localization (Fig. 5, D and E)." It is difficult to see nuclear localization in these panels. Is it suppose to be nuclear in the germline nuclei and oocytes? This is not evident in the data shown. Also, the conclusion that "OOPS-1 and SPE-11 directly interact in vivo" is not possible to draw from this data. Co-localization is not evidence of direct interaction in vivo. It suggest they may, if they were truly shown to colocalize. Panels B and C show co-localization in germline nuclei but not early oocytes.
We have improved Figure 5 as suggested. We have included insets in Figure 5, as recommended. It is not surprising that the colocalization is more readily apparent in the distal germline because that is where OOPS-1 is expressed at higher levels. The inset images in our new Figure 5 make the point that mScarlet::SPE-11 localizes cortically in oocytes when expressed there, albeit at levels that are less than in the distal germline. Importantly, this cortical localization is dependent on OOPS-1.

We have also added a supplemental figure (Fig. S8), showing the expression of GFP::SPE-11 under control of the *oma-1*, *oma-2*, *rme-2*, and *puf-5* promoters and their respective 3'UTRs, as the reviewer asked for in point #17 above. In each of these instances, we observe rescue and cortical localization of GFP::SPE-11 in oocytes where OOPS-1 is established to localize. In these instances, we don't see much GFP::SPE-11 in the distal germline, likely because the endogenous proteins (e.g., OMA-1, OMA-2, RME-2, and PUF-5) are not abundantly expressed there. We point out that when GFP::SPE-11 is made to be very abundant in oocytes (e.g., using the *oma-1* or *oma-2* promoters and their 3'UTRs), we also see nuclear localization. In these instances, we think it likely that binding to OOPS-1 has been saturated. That SPE-11 can localize to the nucleus of oocytes in *oops-1* mutants or when expressed at high levels in the female germline is not surprising. Browning and Strome (1996) expressed SPE-11 in embryos using the heat shock promoter and observed nuclear localization in multicellular embryos.

© 2025. Published by The Company of Biologists under the terms of the Creative Commons Attribution License (https://creativecommons.org/licenses/by/4.0/).

19. Page 13 Lines 258. The call out for Fig. 5 F and G - the authors do not describe what localization they observe for the GFP::OOPS-1A expressed in sperm. It is also hard to tell from the image. It the image depicting that the male sperm was transferred? What is the staining pattern of the GFP::OOPS-1A in sperm? It is hard to tell from the image presented and not discussed in the text.
This is hermaphrodite sperm. We have not examined subcellular localization, as we do not think it is relevant to our conclusions. As mentioned above, we improved the quality of Figure 5 and included insets and additional labels to make our points clearer. Please see Figure 5, panels G and H.

20. Page 13 Line 264 - I could not find where IDP was originally defined.
Line 164, page 8. For additional clarity, we defined it again in the subsequent text (page 14, line 299).

21. Page 14 Line 277 - the authors state "suggesting these regions might mediate their interactions." Having high pLDDT values do not support that these regions mediate interactions. It just suggests that they have higher confidence in predication scores thus more structured.
The AlphaFold 3 models predict the proteins interact. We have clarified the text to (pages14-15, lines 300-305):

"We used the AlphaFold 3 server to generate a binary model complex; each protein in the complex is predicted to exhibit structurally ordered regions with high per residue measures of local confidence using the predicted local distance difference test (pLDDT; Fig. S9). Remarkably, the regions of high pLDDT values in the complex, which occur where AlphaFold 3 predicts the two proteins to interact, correspond well to the essential regions of OOPS-1 and SPE-11 (Fig. 6B,E)."

22. Figure 9 and Discussion:
A summary figure that has more insight into how the complex would function in the roles discussed in the paper would be helpful. The figure as is looks like it indicates that OOPS-1 and SPE-11 associate with CHS-1 directly to regulate eggshell formation. However, the only direct interactor found was GSP-3/4, which are not included in the figure. Why is CHS-1 shown as the direct interactor? Another interaction not included is actin and the idea of the OOPS-1-SPE-11 having a role in actin regulation that would somehow promote cytokinesis, which the authors stated was a function in the abstract twice. The authors promote that the complex function in cytokinesis - what is the model for how OOPS-1-SPE-11 promote this locally to function in cytokinesis and polar body extrusion?
We have included a new model figure as suggested.

23. Are there any other examples in other organisms of such a paternal/maternal partnership? It would strengthen and broaden the work to discuss this - point out the novelty or commonalities that might be known and in what contexts.
The majority of sperm-oocyte protein partnerships described relate to gametic interactions required for fertilization. There are few examples of intracellular interactions of sperm and oocyte proteins that are required for egg activation. We have modified the Discussion (page 19, lines 390-394) to:

"The majority of sperm-oocyte protein partnerships defined thus far mediate gamete recognition, binding, and fusion reactions required for fertilization (reviewed by Deneke and Pauli, 2021)."

Other textual changes

To accommodate the journal's request that the text be no longer than 7000 words, we shortened the Introduction by only discussing the roles of actin regulation in oocyte meiosis in the Discussion, after the biochemical data indicating that the OOPS-1-SPE-11 complex is an actin regulator has been presented.

Second decision letter

© 2025. Published by The Company of Biologists under the terms of the Creative Commons Attribution License (https://creativecommons.org/licenses/by/4.0/).

MS ID#: dev.204674R1

MS TITLE: A sperm-oocyte protein partnership required for egg activation in Caenorhabditis elegans

AUTHORS: Tatsuya Tsukamoto; Ji Kent Kwah; Mark E. Zweifel; Naomi Courtemanche; Micah D. Gearhart; Katherine M. Walstrom; Aimee Jaramillo-Lambert; David Greenstein

Dear Dr Greenstein,

I have now received all the referees reports on the above manuscript, and have reached a decision. The referees' comments are appended below, or you can access them online: please go to .

The overall evaluation is positive and we would like to publish a revised manuscript in Development. However, before I can move forward with accepting the manuscript, we address the suggestion made by Reviewer 2, : Page 12: Please remove the phrase: "...the inescapable inference is that the proteins must interact soon after fertilization." Suggested change: "Together, these data support that OOPS-1 and SPE-11 interact and function after fertilization." I agree with the reviewer that this change will greatly improve the clarity of the phrasing. Please attend to all of the reviewers' comments in your revised manuscript and detail them in your point-by-point response. If you do not agree with any of their criticisms or suggestions explain clearly why this is so.

Reviewer 1

SUMMARY OF THE ADVANCE MADE IN THIS PAPER AND ITS POTENTIAL SIGNIFICANCE TO THE FIELD

Tsukamoto et al. A sperm-oocyte protein partnership required for egg activation in Caenorhabditis elegans

Summary and recommendation
The union of sperm and egg is a dividing line between oogenesis and embryonic development. Early embryonic development is heavily influenced by maternal factors, but paternal factors are much less well understood. The identification of paternal effect mechanisms is rarely defined by mutation. This paper moves the field forward in a significant way by identifying new a new factor (OOPS-1) that interact with a paternally provided gene product (SPE-11) to regulate the completion of meiosis and the oocyte-to-embryo transition. This paper is a rigorous, methodical, and complete report of this work. Arguably, this very data rich paper could have been split into at least two publishable units. I fully support the publication of this paper in Development in its current form.

SUGGESTIONS TO AUTHORS

The authors addressed all my questions and I do not require any changes.

Reviewer 2

SUMMARY OF THE ADVANCE MADE IN THIS PAPER AND ITS POTENTIAL SIGNIFICANCE TO THE FIELD

Suggestions and comments were well-addressed by the authors. The manuscript is improved.

SUGGESTIONS TO AUTHORS

Page 12: Please remove the phrase: "...the inescapable inference is that the proteins must interact soon after fertilization." Suggested change: "Together, these data support that OOPS-1 and SPE-11 interact and function after fertilization."

Second revision

© 2025. Published by The Company of Biologists under the terms of the Creative Commons Attribution License (https://creativecommons.org/licenses/by/4.0/).

Author response to reviewers' comments

Response to Reviewers

We thank the reviewers and the editor for the constructive suggestions and have addressed all points raised.

Reviewer 1

SUMMARY OF THE ADVANCE MADE IN THIS PAPER AND ITS POTENTIAL SIGNIFICANCE TO THE FIELD

Tsukamoto et al. A sperm-oocyte protein partnership required for egg activation in Caenorhabditis elegans

Summary and recommendation
The union of sperm and egg is a dividing line between oogenesis and embryonic development. Early embryonic development is heavily influenced by maternal factors, but paternal factors are much less well understood. The identification of paternal effect mechanisms is rarely defined by mutation. This paper moves the field forward in a significant way by identifying new a new factor (OOPS-1) that interact with a paternally provided gene product (SPE-11) to regulate the completion of meiosis and the oocyte-to-embryo transition. This paper is a rigorous, methodical, and complete report of this work. Arguably, this very data rich paper could have been split into at least two publishable units. I fully support the publication of this paper in Development in its current form.

SUGGESTIONS TO AUTHORS

The authors addressed all my questions and I do not require any changes.

We appreciate the positive assessment! Thank you for reviewing our manuscript.

Reviewer 2

SUMMARY OF THE ADVANCE MADE IN THIS PAPER AND ITS POTENTIAL SIGNIFICANCE TO THE FIELD

Suggestions and comments were well-addressed by the authors. The manuscript is improved.

Thank you for the positive assessment! Your prior comments were very helpful in revising our manuscript.

SUGGESTIONS TO AUTHORS

Page 12: Please remove the phrase: "...the inescapable inference is that the proteins must interact soon after fertilization." Suggested change: "Together, these data support that OOPS-1 and SPE-11 interact and function after fertilization."

We changed the text to, “Together, these data suggest that OOPS-1 and SPE-11 interact and function after fertilization.”

Third decision letter

MS ID#: dev.204674R2

MS TITLE: A sperm-oocyte protein partnership required for egg activation in Caenorhabditis elegans

© 2025. Published by The Company of Biologists under the terms of the Creative Commons Attribution License (https://creativecommons.org/licenses/by/4.0/).

AUTHORS: Tatsuya Tsukamoto; Ji Kent Kwah; Mark E. Zweifel; Naomi Courtemanche; Micah D. Gearhart; Katherine M. Walstrom; Aimee Jaramillo-Lambert; David Greenstein
ARTICLE TYPE: Research Article

Dear Dr Greenstein,

I am happy to tell you that your manuscript has been accepted for publication in Development, pending our standard publication integrity checks.

© 2025. Published by The Company of Biologists under the terms of the Creative Commons Attribution License (https://creativecommons.org/licenses/by/4.0/).
